# National-, institutional-, and individual-level determinants of pharmacology and pharmacy research excellence: an analysis of stanford–elsevier lists of the top 2% scholars (2017–2023)

**DOI:** 10.1007/s00210-025-04949-4

**Published:** 2026-01-30

**Authors:** Abanoub Riad, Lenka Součková, Jitka Rychlíčková, Michal Koščík

**Affiliations:** 1 Masaryk Centre for Global Health (MCGH), Department of Public Health, Faculty of Medicine, Masaryk University, Brno, Czech Republic; 2 Department of Public Health, Faculty of Medicine, Masaryk University, Brno, Czech Republic; 3 Centre of Excellence CREATIC, Faculty of Medicine, Masaryk University, Brno, Czech Republic; 4 Department of Pharmacology, Faculty of Medicine, Masaryk University, Brno, Czech Republic

**Keywords:** Academies and institutes, Bibliometrics, Macroeconomics, Meta-research, Pharmaceutical research, Research personnel

## Abstract

*Research* *excellence* in pharmacology and pharmacy remains insufficiently studied compared with broader analyses of productivity, despite its centrality to advancing drug discovery and health outcomes. This study investigated the multilevel determinants of pharmacology and pharmacy *research excellence*, defined by membership in the Stanford–Elsevier Top 2% Scientists Lists (SEL). We analyzed 56,358 records in SELs between 2017 and 2023 using a multilevel socio-ecological model. Independent variables spanned national determinants (e.g., health system capacity, human development, gender inequality, R&D investment), institutional factors (global and field-specific rankings), and individual characteristics (gender and academic age). Core bibliometric outcomes included citation counts excluding self-citations, modified *H*-index, and composite scores. Excellence was disproportionately concentrated in high-income countries and a limited number of elite institutions, although contributions from middle-income countries expanded over time. National R&D investment, Universal Health Coverage, and Human Development Index values consistently predicted higher bibliometric outcomes, while gender inequality was negatively associated with excellence. Disease burdens mirrored these inequities: conditions common in high-income settings (e.g., cancers, COPD) correlated positively with excellence, whereas major global burdens (e.g., HIV/AIDS, diabetes) correlated negatively. Institutional concentration illustrated the *Matthew effect*, with the top 20 institutions accounting for over one-tenth of global excellence. At the individual level, female scholars remained underrepresented (16.8% in career-long, 25.0% in single-year lists) and showed lower bibliometric performance and younger academic ages than men. Academic age emerged as the strongest positive predictor of research impact, with consistent gains across both datasets. Pharmacology and pharmacy *research excellence* is conditioned by systemic disparities in resources, prestige, and representation. Policy interventions must target these entrenched capacity-based advantages by strengthening research capacity in underrepresented countries and institutions and implementing frameworks for gender-equitable career progression.

**Supplementary Information:**

The online version contains supplementary material available at 10.1007/s00210-025-04949-4.

## Introduction

Pharmacology and pharmacy constitute a unique scientific domain shaped by regulatory, industrial, and translational structures that set it apart from other research fields (Gudi et al. 2022; Lau and Seifert 2025; Mubarak et al. 2024; Rasmussen et al. 2018; European Federation of Pharmaceutical Industries and Associations (EFPIA) 2024; Fowler et al. 2024; Grootendorst et al. 2014; Lesko and Graaf 2025). Research in this area is embedded within stringent regulatory frameworks governed by agencies such as the EMA and FDA, which influence evidence standards, data transparency, and translational pathways (Gudi et al. 2022; Lau and Seifert 2013). Moreover, this field is characterized by an unusually high degree of interdependence between academia, public agencies, and the pharmaceutical industry, producing research networks that differ from those observed in broader biomedicine (Mubarak et al. 2024; Rasmussen et al. 2018). Its R&D intensity is among the highest across high-technology sectors, generating substantial cross-national and cross-institutional concentration of scientific capacity (European Federation of Pharmaceutical Industries and Associations (EFPIA) 2024; Fowler et al. 2024). Intellectual property regimes, including patent systems and exclusivity provisions, shape research agendas and the diffusion of knowledge (Grootendorst et al. 2014). Rapid technological advances in biologics, gene therapies, and mRNA platforms additionally contribute to distinctive temporal dynamics in scholarly influence (Lesko and Graaf 2025). These structural features collectively imply that pharmacology and pharmacy may display patterns of *research excellence* that differ from those observed in other scientific fields. Therefore, a field-specific analysis is warranted to understand how national, institutional, and individual factors interact within this research ecosystem to shape the distribution and intensity of excellence.

Existing bibliometric analyses displayed that pharmacology and pharmacy scholarship are unevenly distributed across the globe (Bibliometric analysis of pharmacology 2025; Wang et al. 2022). Research output and citation impact are predominantly concentrated within high-income and English-speaking countries, where stronger research infrastructures, mature funding systems, and greater integration into global scholarly networks facilitate disproportionate scientific visibility (Bibliometric analysis of pharmacology 2025; Wang et al. 2022). Linguistic dynamics further reinforce these inequalities, as the dominance of English-language publishing advantages scholars embedded in Anglophone environments while constraining the international reach of non-Anglophone researchers (Di Bitetti and Ferreras 2016). At the institutional level, high-impact contributions are similarly clustered within a limited group of prestigious universities that possess long-standing scientific capital, extensive collaborative linkages, and enhanced access to competitive funding streams (Thompson 2019). These institutional asymmetries mirror broader stratification patterns within the biomedical sciences (Kozlowski et al. 2024). Individual-level disparities also shape scholarly influence: gender gaps persist in authorship, leadership roles, and cumulative citation indicators, with women often exhibiting systematically lower long-term metrics despite comparable scientific merit (Sketris et al. 2022). Academic age serves as an additional determinant of research impact, as citation influence typically accrues over extended career trajectories through sustained productivity, network consolidation, and cumulative advantage (Abramo et al. 2011; Zaorsky et al. 2020). Taken together, these gradients illustrate the uneven patterns of research production and citation influence in pharmacology and pharmacy and highlight the need to understand how multilevel factors shape these outcomes.

Notwithstanding these insights about production patterns of pharmacology and pharmacy scholarship, there is a paucity of evidence about *research excellence* in this field. *Research excellence* emphasizes the quality, originality, rigor, and broader influence of scientific work, offering a more meaningful indicator of disciplinary advancement than publication counts alone (Pontika et al. 2022; Ofir and Schwandt 2012). National and international research frameworks increasingly prioritize excellence-based evaluation, as seen in the UK Research Excellence Framework and the European Union’s Horizon programs, which reward high-quality and field-shaping scholarship rather than sheer output volume (Kelly 2016; European Research Council (ERC) 2025;). As governments and funding bodies move towards excellence-centered assessment, understanding how excellence emerges and is distributed becomes essential for informing strategic investment, strengthening research capacity, and guiding development across this discipline (Tijssen and Kraemer-Mbula 2018; Ferretti et al. 2018).

Existing bibliometric studies of pharmacology and pharmacy rely on productivity indicators or unadjusted citation counts (Bibliometric analysis of pharmacology 2025). They do not assess *research excellence* which may require rigorous, field-normalized, and co-authorship-adjusted metrics (Chen et al. 2021). Current excellence-oriented bibliometric work is not disaggregated by discipline (Kuc-Czarnecka and Saltelli 2025). As a result, nothing is known about excellence patterns unique to pharmacology and pharmacy, despite the highly distinctive regulatory, industrial, and translational profile of this field. Although the economics of science literature documents broad inequalities at national and institutional levels, the magnitude and structure of these disparities within pharmacology and pharmacy remain empirically unexamined (Amano et al. 2025). Moreover, no existing study examines how national, institutional, and individual determinants jointly shape excellence in this discipline.

To mitigate these shortcomings of citation-based metrics, which are vulnerable to manipulation through practices such as excessive self-citation, salami publications, gift authorship, and inflated multi-authorship (Szomszor et al. 2021; Ioannidis and Boyack 2020; Ioannidis and Maniadis 2023), the “science-wide author databases of standardized citation indicators”, commonly known as the Stanford–Elsevier Lists (SEL), were developed as a robust, transparent, and frequently updated framework for assessing global scientific excellence (Ioannidis et al. 2019, 2020). The SEL identify the top 2% of scientists worldwide based on Scopus data, using a composite score (*C*-score) that combines multiple citation metrics, e.g., total citations, modified *H*-index, authorship position- and co-author-count-adjusted indices, and the exclusion of self-citations, thereby providing a more objective and reproducible measure of research impact (Ioannidis et al. 2019, 2020).

A key epistemological feature of the SEL is its use of the Science-Metrix classification, which categorizes research outputs into five domains (e.g., health sciences), 20 fields (e.g., clinical medicine), and 174 subfields (e.g., pharmacology and pharmacy) (Science-Metrix. 2025). The SEL assign each scholar a primary and secondary subfield based on publication frequency, enabling field-normalized comparisons (Ioannidis et al. 2019, 2020). *Pharmacology and pharmacy* constitutes one of these subfields, allowing the SEL to identify scholars who have made the most influential and field-adjusted contributions to this discipline (Science-Metrix. 2025).

The present study examines the determinants of *research excellence* in pharmacology and pharmacy based on scholar records identified in the SEL. Adopting a socio-ecological framework, it evaluates how national, institutional, and individual factors jointly shape the distribution of excellent pharmacology and pharmacy scholars (EPPS) and their key performance indicators, including the modified *H*-index, composite score (*C*-score), and citation counts excluding self-citations.

## Materials and methods

### Study design

**Fig. 1:**
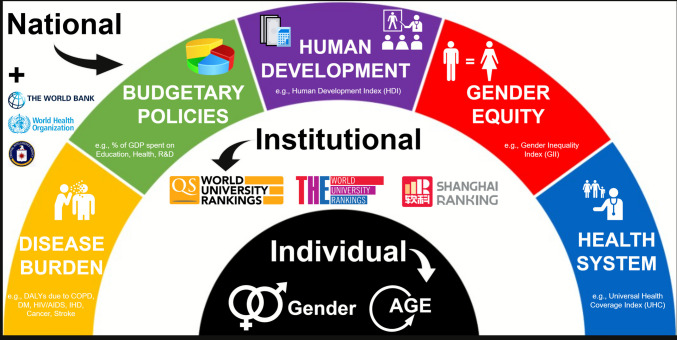
Theoretical framework of multilevel determinants shaping pharmacology and pharmacy research excellence: individual-, institutional-, and national-level predictors

The study adopted a bibliometric approach based on an ecological framework with three distinct levels of determinants of pharmacology and pharmacy *research excellence*. The national level comprised system-level factors, including health system characteristics, gender equity indicators, human development, budgetary policies, and disease burden. The institutional level was evaluated through overall and field-specific academic rankings. The individual level addressed gender and academic age. The conceptual representation of these levels is provided in Fig. 1. All elements of design and reporting conformed to the C*hecklist for Bibliometric Reviews of the Biomedical Literature* (BIBLIO) (Montazeri et al. 2023).

### Data sources

The primary data source consisted of the *science-wide author databases of standardized citation indicators*, better known as the Stanford–Elsevier Lists (SEL) (Ioannidis et al. 2019, 2020). For this study, seven annual updates covering the years 2017 through 2023 were included, encompassing both *career-long* and *single-year* datasets. All files were obtained from the Elsevier Data Repository®. All data were extracted in August 2025 (Ioannidis 2024).

Secondary sources were incorporated to provide national- and institutional-level determinants, including:

- Health system variables, including the Universal Health Coverage Index (UHC), provided by the World Health Organization (WHO) data repository (World Health Organization (WHO) 2024).
- Gender equity indicators, including the Gender Inequality Index (GII) and its components, available from the United Nations Development Programme (UNDP) Human Development Reports (United Nations (UN) 2025).
- Human development indicators, including the Human Development Index (HDI) and its components, from the UNDP Human Development Reports (United Nations Development Programme (UNDP) 2025).
- Budgetary policy measures, including gross domestic product (GDP) expenditure shares on education, health, and R&D, obtained from the World Bank Open Data repository (The World Bank 2025).
- Measures of disease burden, exemplified by disability-adjusted life years (DALYs) attributed to diabetes mellitus, ischemic heart disease (IHD), and stroke, sourced from the Global Burden of Disease (GBD) 2021 database (Institute for Health Metrics and Evaluation (IHME) 2025).
- Official language data were sourced from the CIA World Factbook; when multiple existed, the most widely spoken official language was selected for each country (Central Intelligence Agency (CIA) 2025).
- University ranking indicators retrieved from QS World University Rankings, Times Higher Education (THE), and the Academic Ranking of World Universities (ARWU) by ShanghaiRanking (Quacquarelli Symonds (QS) 2025; Times Higher Education (THE) 2025; Shanghai Ranking Consultancy 2025).

### Data cleaning and pre-processing

At the outset, the *career-long* and *single-year* SEL datasets were restricted to scholars whose disciplinary designation was *pharmacology and pharmacy* in either subfield 1 or subfield 2. Entries outside this scope were excluded.

Subsequent gender prediction was performed using *Genderize.io*, a platform developed by Demografix ApS (Roskilde, Denmark) (Demografix ApS 2025). The tool employs large-scale probabilistic modelling of over 900 million first-name associations, considering country-specific context and drawing primarily from social media data (Demografix ApS 2025). In the extracted datasets, 56,358 records were analyzed. Gender assignment was not possible in 4904 cases (8.7%), mainly due to the presence of one-letter initials or names with extremely rare frequency, in accordance with the 99% certainty threshold.

Institutional data underwent a final phase of manual verification. This included harmonization of name variants across different languages and abbreviation systems, correction of transliterations, and consolidation of duplicate institutional entries.

### Independent variables

National determinants (*n* = 29) were structured into five thematic categories:

1. Health system attributes included the Universal Health Coverage Index (UHC), the general government expenditure on health (%), and the number of pharmacists per 100,000 population, together reflecting accessibility and workforce capacity in healthcare.
2. Gender equity variables included the Gender Inequality Index (GII), maternal mortality per 100,000 live births, adolescent birth rate (per 1000 women aged 15–19), education gap in the 25 + age group (male − female), employment gap in the 15 + age group (male − female), and female share of parliamentary seats (%), providing multiple indicators of gender differences.
3. Human development was captured by the Human Development Index (HDI), life expectancy at birth (years), expected years of schooling, mean years of schooling, and gross national income per capita (USD).
4. Budgetary indicators included % GDP spent on research and development, % GDP spent on health, and % GDP spent on education.
5. Disease burden measures comprised DALYs attributed to COPD, diabetes mellitus, HIV/AIDS, ischemic heart disease, neoplasms, and stroke.

In addition, the World Bank level, the WHO region, and the official language were considered.

Institutional determinants (*n* = 12) were derived from global rankings: QS (pharmacy (overall score, academic reputation), general (overall score, academic reputation)), THE (medicine (overall score, research quality), general (overall score, research quality)), and ARWU (pharmacy (overall score, research impact), general (overall score, per capita performance)).

Individual determinants (*n* = 2) included gender, inferred using the *Genderize.io* algorithm, and academic age, defined as the years elapsed between the first and most recent Scopus-indexed publication of each scholar.

### Dependent variables

The key outcome was the number of excellent pharmacology and pharmacy scholars (EPPS) identified per country and institution, based on both the *career-long* and *single-year* SEL.

To operationalize *research excellence*, four core bibliometric indicators were utilized: (a) citation count excluding self-citations; (b) modified *H*-index, defined as an adjusted *H*-index that controls for the number of co-authors per publication and excludes self-citations; (c) composite score (*C*-score), a proprietary SEL metric that integrates six citation-based indicators with adjustments for disciplinary field and authorship position, excluding self-citations; and (d) percentage of self-citations.

Furthermore, authorship role-specific secondary indicators were used to complement analyses linked to academic age. These included the number and citation counts of (a) single-authored publications, (b) single- and first-authored publications, and (c) single-, first-, and last-authored publications.

### Statistical analyses

The analytical strategy commenced with descriptive statistics. Categorical variables (e.g., WHO region) and ordinal variables (e.g., World Bank level) were tabulated as frequencies (*n*) and percentages (*%*). Numerical variables, such as citation counts, were summarized by medians and interquartile ranges (IQR). The Shapiro–Wilk test was employed to test the assumption of normal distribution, with *p*-values < 0.05 indicating non-normality. Univariable analyses were then undertaken. Depending on the data type, Chi-squared tests, Fisher’s exact tests, Mann–Whitney *U* tests, and Spearman’s rho correlations were applied, with significance defined as < 0.05.

Regression analyses were subsequently applied to address several objectives: (a) simple logistic regression was used to model the probability of female gender membership; (b) linear regression models were run with academic age as the sole predictor of bibliometric outcomes; (c) linear regression models were estimated for core bibliometric outcomes with national-level determinants as predictors while adjusting for individual-level determinants (gender and academic age); and (d) multivariable linear regression models for core bibliometric outcomes were constructed to incorporate individual-level and thematic national-level determinants simultaneously, enabling evaluation of their independent effects.

## Results

A total of 56,358 EPPS records were identified, consisting of 31,060 (55.1%) from the *career-long* SEL and 25,298 (44.9%) from the *single-year* SEL. Data completeness was high, with country affiliation available for 98.3% of EPPS, institutional affiliation for 98.5%, gender for 91.3%, and academic age for 96.3%. The number of EPPS rose consistently across the SEL annual releases, from 3036 in 2017 to 5645 in 2023 in the *career-long* SEL and from 2089 to 5195 in the *single-year* SEL over the same interval.

### National-level analyses of pharmacology and pharmacy research excellence

High-income countries represented the majority of EPPS, accounting for 93.0% in the *career-long* SEL and 77.5% in the *single-year* SEL, though the latter proportion reflects an increasing diversity of contributing regions. Upper-middle-income countries expanded their share from 1.8% and 4.5% in 2017 to 5.9% and 17.2% in 2023, while lower-middle-income countries increased from 0.7% and 2.4% to 3.7% and 11.7% over the same interval. At the regional level, EURO predominated (41.2% and 35.4%), followed by AMRO (39.7% and 29.8%) and WPRO (14.8% and 19.5%). English-speaking countries remained the largest linguistic group, comprising 53.7% of EPPS in the *career-long* SEL and 40.5% in the *single-year* SEL. This dominance declined most sharply in the *single-year* SEL, where the contribution dropped from 49.2 to 35.6% (Table 1). At the country level, the US contributed 34.9% and 25.4% of EPPS, with the UK second (11.4% and 7.9%) (Tables S1–S2).

**Table 1:** National-level analysis: distribution of pharmacologic scholars and their citation counts in the *career-long* and *single-year* Stanford–Elsevier Lists (SEL) of top scientists worldwide (2017–2023), stratified by World Bank classification (FY 2024) and official language (CIA World Factbook)

*Career-long* SEL											
	**Variable**	**Outcome**	**SEL 2017**	**SEL 2018**		**SEL 2019**	**SEL 2020**	**SEL 2021**	**SEL 2022**	**SEL 2023**	**Total ▼**
Scholars *N* (%)	World Bank	High	2511 (97.55%)	2706 (97.34%)		3698 (94.60%)	4678 (92.73%)	4780 (92.17%)	4902 (91.25%)	5069 (90.45%)	28,344 (93.02%)
		Upper-middle	46 (1.79%)	49 (1.76%)		141 (3.61%)	232 (4.60%)	257 (4.96%)	284 (5.29%)	328 (5.85%)	1337 (4.39%)
		Lower-middle	17 (0.66%)	24 (0.86%)		67 (1.71%)	134 (2.66%)	147 (2.83%)	184 (3.43%)	207 (3.69%)	780 (2.56%)
		Low	0 (0.00%)	1 (0.04%)		3 (0.08%)	1 (0.02%)	2 (0.04%)	2 (0.04%)	0 (0.00%)	9 (0.03%)
	WHO region	EURO	1105 (42.93%)	1180 (42.45%)		1603 (41.01%)	2084 (41.31%)	2130 (41.07%)	2175 (40.49%)	2270 (40.51%)	12,547 (41.18%)
		AMRO	1145 (44.48%)	1241 (44.64%)		1620 (41.44%)	1964 (38.93%)	2011 (38.78%)	2031 (37.81%)	2089 (37.28%)	12,101 (39.71%)
		WPRO	291 (11.31%)	322 (11.58%)		570 (14.58%)	778 (15.42%)	797 (15.37%)	861 (16.03%)	891 (15.90%)	4510 (14.80%)
		EMRO	15 (0.58%)	12 (0.43%)		52 (1.33%)	105 (2.08%)	119 (2.29%)	145 (2.70%)	168 (3.00%)	616 (2.02%)
		SEARO	14 (0.54%)	19 (0.68%)		53 (1.36%)	99 (1.96%)	114 (2.20%)	141 (2.62%)	162 (2.89%)	602 (1.98%)
		AFRO	4 (0.16%)	6 (0.22%)		11 (0.28%)	15 (0.30%)	15 (0.29%)	19 (0.35%)	24 (0.43%)	94 (0.31%)
	Official language	English	1598 (52.64%)	1719 (61.72%)		2226 (56.63%)	2715 (53.57%)	2757 (52.98%)	2797 (51.88%)	2878 (50.98%)	16,690 (53.73%)
		German	224 (7.38%)	229 (8.22%)		336 (8.55%)	421 (8.31%)	424 (8.15%)	431 (7.99%)	450 (7.97%)	2515 (8.10%)
		Japanese	129 (4.25%)	147 (5.28%)		247 (6.28%)	304 (6.00%)	306 (5.88%)	332 (6.16%)	327 (5.79%)	1792 (5.77%)
		Italian	107 (3.52%)	127 (4.56%)		159 (4.04%)	222 (4.38%)	220 (4.23%)	226 (4.19%)	235 (4.16%)	1296 (4.17%)
		Chinese	36 (1.19%)	42 (1.51%)		88 (2.24%)	137 (2.70%)	144 (2.77%)	159 (2.95%)	178 (3.15%)	784 (2.52%)
		Other	942 (31.03%)	521 (18.71%)		875 (22.26%)	1269 (25.04%)	1353 (26.00%)	1446 (26.82%)	1577 (27.94%)	7983 (25.70%)
Citations *median* (IQR)	World Bank	High	6634 (4248–10,781)	7378 (4845–11,917)		5852 (3600–9698)	5694 (3388–9682)	5788 (3431–9889)	6212 (3642–10,470)	6390 (3763–10,819)	6203 (3741–10,416)
		Upper-middle	5880 (3509–8222)	5915 (3881–9815)		5236 (3357–8720)	5259 (3107–8301)	5375 (3201–8457)	6092 (3444–9931)	6751 (3788–10,698)	5893 (3436–9451)
		Lower-middle	5803 (4532–8414)	6350 (4740–10103)		3467 (2464–6071)	3416 (2228–5151)	3369 (2344–5434)	3866 (2556–6018)	3925 (2590–6481)	3778 (2465–6075)
		Low	NA	4081 (4081–4081)		2830 (2216–3518)	2062 (2062–2062)	2678 (2401–2954)	7212 (5049–9376)	NA	2886 (2124–4081)
	WHO region	EURO	6749 (4406–10410)	7570 (5090–11550)		6080 (3652–9906)	5996 (3506–9952)	6060 (3502–10211)	6512 (3732–10,834)	6725 (3908–11330)	6458 (3840–10623)
		AMRO	6498 (4147–11,172)	7251 (4639–12,464)		5677 (3522–9665)	5593 (3254–9581)	5647 (3308–9776)	6028 (3472–10,440)	6221 (3609–10806)	6056 (3620–10459)
		WPRO	6634 (4414–9944)	7220 (4818–11,107)		5824 (3797–9285)	5557 (3552–8936)	5749 (3671–9476)	6198 (4003–9985)	6582 (4169–10,468)	6093 (3914–9790)
		EMRO	5154 (3263–6806)	5588 (4180–7794)		2988 (2188–4650)	3052 (2014–4979)	3287 (2118–5172)	3590 (2282–6358)	3695 (2517–6314)	3454 (2315–5749)
		SEARO	5666 (4549–7180)	6336 (5218–9332)		4348 (3084–6477)	4267 (2680–5708)	4394 (2716–5815)	5020 (3040–6414)	5313 (2967–7334)	4754 (2906–6548)
		AFRO	2348 (2066–2665)	3001 (2593–3038)		2800 (2136–3187)	2533 (1962–3412)	2933 (2080–3540)	2886 (2364–3816)	3264 (2400–4083)	2910 (2238–3603)
	Official language	English	6304 (4140–10720)	7088 (4592–11,801)		5698 (3495–9558)	5529 (3248–9517)	5628 (3297–9792)	5946 (3472–10,371)	6142 (3605–10742)	5976 (3608–10291)
		German	6811 (4612–10,777)	7537 (5332–11,422)		6026 (3468–9439)	5907 (3146–9646)	5974 (3089–9958)	6633 (3292–10,554)	6819 (3449–11,039)	6512 (3576–10,338)
		Japanese	7734 (4930–11741)	8165 (5376–12,103)		6228 (4083–9735)	5738 (3848–8921)	5803 (3806–9136)	6295 (4238–9790)	6158 (4066–9765)	6298 (4152–9881)
		Italian	7413 (5394–11,226)	8250 (5632–12,978)		6760 (4438–10,408)	7530 (5032–11878)	7398 (4748–12,088)	8034 (5190–12892)	8259 (5366–12,760)	7632 (5070–12041)
		Mandarin	6047 (4436–9407)	6085 (4770–9714)		6417 (4622–9097)	6816 (4608–10111)	7045 (4596–10,642)	7991 (5288–11,312)	8782 (5868–12,172)	7416 (4840–10784)
		Other	6164 (3964–9866)	7837 (5005–11914)		5560 (3352–9580)	5136 (3111–9104)	5248 (3118–9109)	5646 (3313–9626)	5903 (3449–9924)	5710 (3444–9680)
***Single-year* SEL**											
	**Variable**	**Outcome**	**SEL 2017**		**SEL 2019**	**SEL 2020**	**SEL 2021**	**SEL 2022**	**SEL 2023**		**Total ▼**
Scholars *N* (%)	World Bank	High	1660 (93.05%)		2941 (83.55%)	3663 (78.44%)	3698 (77.23%)	3679 (74.01%)	3673 (70.91%)		19,314 (77.53%)
		Upper-middle	81 (4.54%)		391 (11.11%)	639 (13.68%)	679 (14.18%)	780 (15.69%)	892 (17.22%)		3462 (13.90%)
		Lower-middle	43 (2.41%)		187 (5.31%)	366 (7.84%)	407 (8.50%)	507 (10.20%)	608 (11.74%)		2118 (8.50%)
		Low	0 (0.00%)		1 (0.03%)	2 (0.04%)	4 (0.08%)	5 (0.10%)	7 (0.14%)		19 (0.08%)
	WHO region	EURO	736 (41.26%)		1299 (36.90%)	1681 (36.00%)	1729 (36.11%)	1679 (33.78%)	1688 (32.59%)		8812 (35.37%)
		AMRO	722 (40.47%)		1180 (33.52%)	1408 (30.15%)	1387 (28.97%)	1367 (27.50%)	1356 (26.18%)		7420 (29.78%)
		WPRO	258 (14.46%)		702 (19.94%)	922 (19.74%)	937 (19.57%)	1002 (20.16%)	1032 (19.92%)		4853 (19.48%)
		EMRO	30 (1.68%)		173 (4.91%)	352 (7.54%)	413 (8.63%)	520 (10.46%)	632 (12.20%)		2120 (8.51%)
		SEARO	34 (1.91%)		151 (4.29%)	287 (6.15%)	296 (6.18%)	367 (7.38%)	432 (8.34%)		1567 (6.29%)
		AFRO	4 (0.22%)		15 (0.43%)	20 (0.43%)	26 (0.54%)	36 (0.72%)	40 (0.77%)		141 (0.57%)
	Official language	English	1028 (49.21%)		1640 (46.20%)	1942 (41.42%)	1901 (39.65%)	1883 (37.81%)	1849 (35.59%)		10,243 (40.49%)
		Chinese	60 (2.87%)		227 (6.39%)	343 (7.31%)	362 (7.55%)	411 (8.25%)	462 (8.89%)		1865 (7.37%)
		German	146 (6.99%)		258 (7.27%)	317 (6.76%)	322 (6.72%)	298 (5.98%)	283 (5.45%)		1624 (6.42%)
		Italian	80 (3.83%)		179 (5.04%)	264 (5.63%)	283 (5.90%)	281 (5.64%)	297 (5.72%)		1384 (5.47%)
		Japanese	77 (3.69%)		151 (4.25%)	185 (3.95%)	178 (3.71%)	178 (3.57%)	161 (3.10%)		930 (3.68%)
		Other	698 (33.41%)		1095 (30.85%)	1638 (34.93%)	1749 (36.48%)	1929 (38.73%)	2143 (41.25%)		9252 (36.57%)
Citations *median* (IQR)	World Bank	High	600 (395–955)		641 (409–1037)	778 (485–1284)	536 (340–888)	562 (353–937)	538 (340–904)		602 (378–1009)
		Upper-middle	645 (477–908)		681 (414–1030)	834 (552–1360)	584 (385–958)	620 (418–1006)	620 (406–1006)		656 (426–1072)
		Lower-middle	563 (407–740)		465 (312–716)	541 (373–867)	393 (276–636)	432 (290–687)	425 (285–674)		448 (297–704)
		Low	NA		157 (157–157)	1226 (930–1523)	247 (148–576)	662 (508–1410)	448 (266–858)		489 (246–1289)
	WHO region	EURO	600 (396–973)		684 (449–1089)	849 (534–1346)	573 (368–933)	600 (380–997)	572 (375–980)		643 (406–1071)
		AMRO	575 (388–918)		612 (379–992)	738 (457–1236)	506 (321–854)	530 (324–903)	502 (308–852)		568 (350–970)
		WPRO	668 (467–965)		665 (424–1089)	824 (543–1358)	580 (383–980)	607 (411–1014)	620 (393–992)		660 (423–1076)
		EMRO	514 (338–934)		432 (287–726)	556 (358–929)	398 (283–634)	478 (317–756)	455 (296–750)		470 (303–763)
		SEARO	596 (450–677)		525 (340–774)	595 (412–922)	430 (297–673)	459 (314–702)	465 (322–701)		489 (328–734)
		AFRO	320 (221–449)		409 (219–790)	574 (360–1003)	328 (172–617)	361 (207–842)	436 (207–854)		410 (210–761)
	Official language	English	576 (378–917)		622 (388–1024)	752 (461–1266)	517 (324–874)	536 (325–909)	512 (311–881)		579 (353–996)
		Chinese	691 (484–955)		807 (512–1246)	1008 (626–1546)	690 (438–1110)	705 (492–1106)	702 (472–1104)		752 (502–1194)
		German	590 (452–861)		633 (389–928)	798 (480–1272)	534 (328–851)	557 (352–898)	543 (324–882)		600 (371–968)
		Italian	707 (464–1064)		773 (490–1123)	934 (624–1521)	611 (428–1049)	701 (472–1176)	629 (430–1028)		724 (482–1171)
		Japanese	597 (472–898)		608 (402–914)	711 (466–1153)	486 (316–762)	477 (364–766)	433 (300–718)		548 (368–881)
		Other	542 (334–910)		615 (390–964)	713 (457–1149)	504 (322–807)	528 (349–844)	518 (339–828)		562 (359–913)

**Fig. 2:**
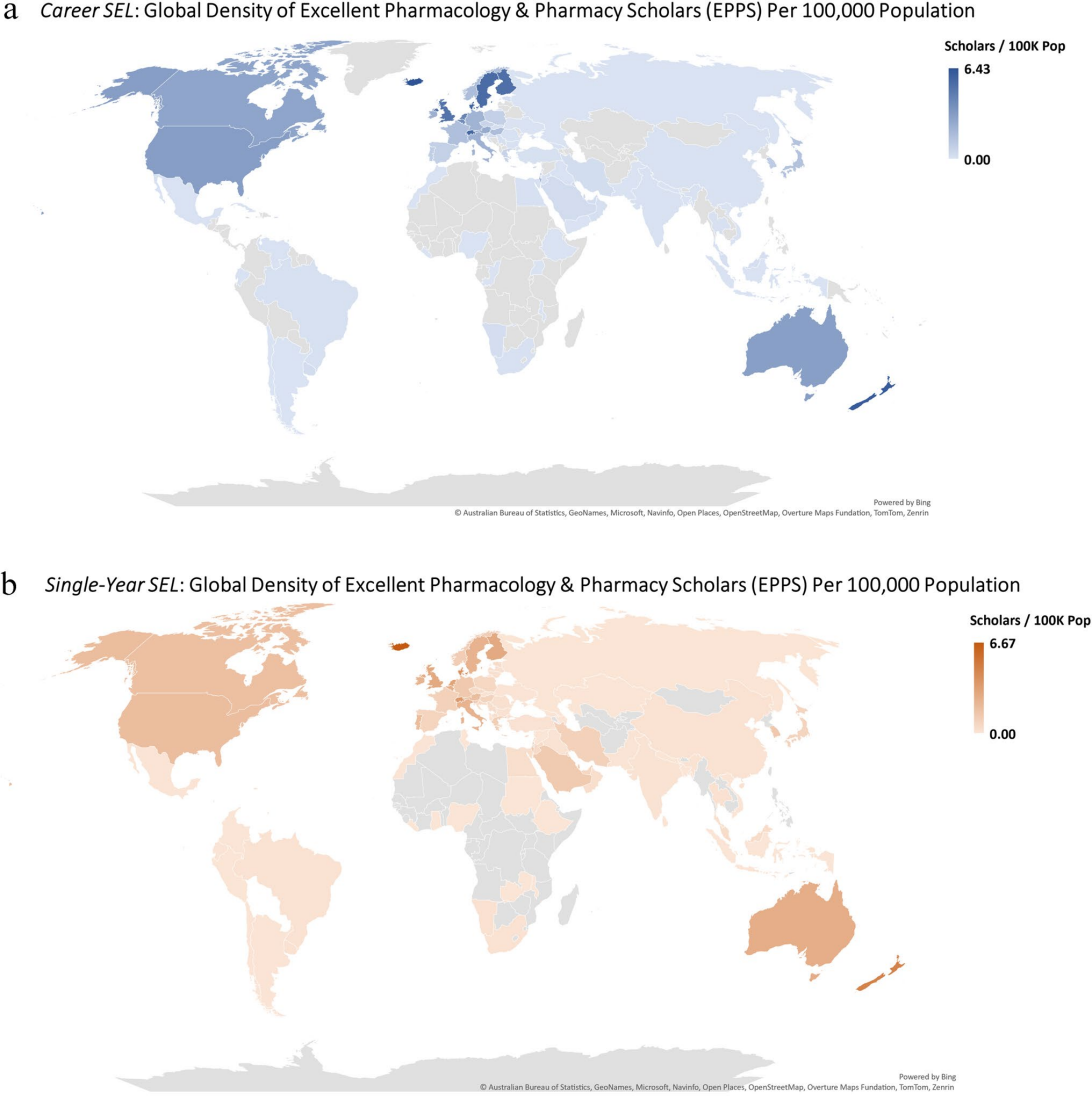
Global density of excellent pharmacology and pharmacy scholars (EPPS) in the Stanford–Elsevier Top 2% Lists per 100,000 Population (2017–2023): **a** *career-long* and **b** *single-year* lists

The global density of EPPS per 100,000 population was 0.456 in the *career-long* SEL and 0.365 in the *single-year* SEL. Substantial inequalities were evident across income groups, with high-income countries reaching 2.043 and 1.387, compared with 0.004 and 0.008 in low-income countries. Across WHO regions, EURO reported the highest densities (1.536 and 1.028), while AFRO had the lowest (0.018 and 0.027) (Table 2). By country, Iceland (6.43 and 6.67) had the highest density, followed by New Zealand (6.09 and 4.72) and Denmark (5.77 and 4.00) (Fig. 2).

**Table 2:** National-level analysis: population density of pharmacologic scholars and their citation counts in the *career-long* and *single-year* Stanford–Elsevier Lists (SEL) of top scientists worldwide (2017–2023), stratified by world bank classification (FY 2024) and official language (CIA World Factbook)

Variable	Outcome	*Career-long* SEL		*Single-year* SEL	
		**Scholars *per* 100 K Pop**	**Citations *per* 100 K Pop**	**Scholars *per* 100 K Pop**	**Citations *per* 100 K Pop**
World Bank	High-income	2.043	17,773.655	1.387	1211.732
	Upper-middle-income	0.052	378.498	0.13	116.323
	Lower-middle-income	0.032	158.338	0.084	49.632
	Low-income	0.004	13.828	0.008	7.68
WHO region	EURO	1.536	13,592.55	1.028	963.857
	AMRO	1.415	12,410.773	0.822	680.465
	WPRO	0.242	1941.509	0.265	234.277
	EMRO	0.092	465.048	0.297	210.512
	SEARO	0.030	161.94	0.077	47.361
	AFRO	0.018	58.611	0.027	16.138
Official language	English	2.128	18,381.186	1.333	1141.708
	German	2.472	20,797.977	1.596	1345.792
	Japanese	1.445	11,928.502	0.75	555.087
	Italian	2.197	22,081.633	2.346	2423.886
	Chinese	0.039	357.393	0.121	115.621
	Other	0.178	1444.004	0.205	166.497
Total	*N*/population size	0.456	3892.193	0.365	310.74

At the national level, EPPS counts correlated positively and moderately to strongly with health system attributes, human development measures, and budgetary policy indicators, while negative associations were observed with the Gender Inequality Index and most of its components. For disease burden, positive correlations were seen with DALYs attributed to COPD and cancers, whereas DALYs attributed to HIV/AIDS, diabetes mellitus, ischemic heart disease, and stroke correlated negatively. These patterns were also found in the bibliometric outcomes and were consistent across the *career-long* and *single-year* SEL (Table 3).

**Table 3:** National-level analyses: correlations between health system characteristics, gender equity, human development, budgetary policies, and disease burden, with the number of excellent pharmacologic scholars and their scholarly output metrics in the Stanford–Elsevier Lists (2017–2023)

Domain	Indicator	Scholars, *N*	Citations, *N*	Modified *H*-index	Composite score		Self-citation %
*Career-long* SEL							
Health system	Universal Health Coverage Index (UHC)	0.579**	0.625**	0.527**	0.372**		0.122
	No. of pharmacists per capita	0.484**	0.499**	0.406**	0.240*		0.131
	General government expenditure on health (%)	0.420**	0.443**	0.281*	0.175		− 0.158
Gender equity	Gender Inequality Index (GII)	− 0.525**	− 0.571**	− 0.435**	− 0.317**		− 0.062
	Maternal mortality per 100 K live births	− 0.510**	− 0.557**	− 0.431**	− 0.315**		− 0.116
	Adolescent birth rate (*per* 1 K women aged 15–19)	− 0.573**	− 0.618**	− 0.477**	− 0.314**		− 0.04
	Education gap 25 + (male − female)	− 0.146	− 0.153	− 0.216*	− 0.344**		− 0.097
	Employment gap 15 + (male − female)	− 0.247*	− 0.261*	− 0.335**	− 0.318**		0.319**
	Female share of parliamentary seats (%)	0.222*	0.235*	0.103	0.16		− 0.11
Human development	Human Development Index (HDI)	0.620**	0.664**	0.517**	0.419**		− 0.029
	Life expectancy at birth (years)	0.563**	0.625**	0.545**	0.410**		0
	Expected years of schooling	0.521**	0.552**	0.409**	0.299**		− 0.001
	Mean years of schooling	0.487**	0.516**	0.428**	0.399**		− 0.015
	Gross national income per capita (USD)	0.596**	0.638**	0.495**	0.424**		0.016
Budgetary policies	% GDP spent on research and development	0.740**	0.755**	0.316**	0.224*		− 0.172
	% GDP spent on health	0.379**	0.391**	0.346**	0.413**		− 0.056
	% GDP spent on education	0.126	0.135	0.207	0.091		− 0.079
Disease burden	DALYs attributed to COPD	0.382**	0.387**	0.205	0.115		− 0.086
	DALYs attributed to diabetes mellitus	− 0.084	− 0.099	− 0.142	− 0.122		0.177
	DALYs attributed to HIV/AIDS	− 0.396**	− 0.391**	− 0.257*	− 0.134		− 0.176
	DALYs attributed to ischemic heart disease	− 0.056	− 0.101	− 0.163	− 0.243*		0.333**
	DALYs attributed to neoplasms	0.333**	0.344**	0.271*	0.167		0.128
	DALYs attributed to stroke	− 0.133	− 0.203	− 0.274*	− 0.322**		0.198
*Single-year* SEL							
Health system	Universal Health Coverage Index (UHC)	0.479**	0.492**	0.394**		0.333**	− 0.017
	No. of pharmacists per capita	0.440**	0.461**	0.303**		0.276*	0.086
	General government expenditure on health (%)	0.384**	0.364**	0.318**		0.222*	− 0.12
Gender equity	Gender Inequality Index (GII)	− 0.423**	− 0.441**	− 0.439**		− 0.419**	0.015
	Maternal mortality per 100 K live births	− 0.392**	− 0.406**	− 0.367**		− 0.387**	− 0.014
	Adolescent birth rate (*per* 1 K women aged 15–19)	− 0.458**	− 0.461**	− 0.351**		− 0.260*	0.063
	Education gap 25 + (male − female)	− 0.015	− 0.036	− 0.212*		− 0.428**	0.098
	Employment gap 15 + (male − female)	− 0.065	− 0.108	− 0.182		− 0.272**	0.108
	Female share of parliamentary seats (%)	0.19	0.201	0.203		0.286**	− 0.083
Human development	Human Development Index (HDI)	0.501**	0.518**	0.445**		0.462**	− 0.036
	Life expectancy at birth (years)	0.495**	0.494**	0.403**		0.357**	− 0.103
	Expected years of schooling	0.448**	0.439**	0.378**		0.363**	− 0.027
	Mean years of schooling	0.353**	0.386**	0.395**		0.472**	0.015
	Gross national income per capita (USD)	0.453**	0.473**	0.402**		0.402**	− 0.026
Budgetary policies	% GDP spent on research and development	0.680**	0.683**	0.385**		0.321**	− 0.011
	% GDP spent on health	0.335**	0.296**	0.181		0.362**	− 0.078
	% GDP spent on education	0.175	0.186	0.280**		0.267**	− 0.164
Disease burden	DALYs attributed to COPD	0.371**	0.379**	0.133		0.092	− 0.101
	DALYs attributed to diabetes mellitus	− 0.01	− 0.055	− 0.065		− 0.069	− 0.043
	DALYs attributed to HIV/AIDS	− 0.258*	− 0.227*	− 0.06		− 0.075	− 0.006
	DALYs attributed to ischemic heart disease	− 0.098	− 0.089	− 0.133		− 0.047	0.174
	DALYs attributed to neoplasms	0.237*	0.256*	0.304**		0.332**	0.044
	DALYs attributed to stroke	− 0.175	− 0.172	− 0.257*		− 0.191	0.092

^**^Correlation is significant at the 0.01 level (two-tailed). *Correlation is significant at the 0.05 level (two-tailed)

### Institutional-level analyses of pharmacology and pharmacy research excellence

The top 20 institutions accounted for 12.7% of EPPS worldwide in the *career-long* SEL and 10.3% in the *single-year* SEL. While all leading institutions in the *career-long* dataset were located in high-income countries, the *single-year* SEL included institutions from lower-middle- and upper-middle-income countries, namely Tabriz University of Medical Sciences, China Pharmaceutical University, and the Chinese Academy of Medical Sciences (Table 4). The number of EPPS per institution correlated most strongly with the ARWU general ranking in the *single-year* SEL (0.704), followed by QS pharmacy academic reputation (0.662), indicating that broader institutional standing and field-specific prestige both influenced institutional productivity (Table 5).

**Table 4:** Institutional-level analysis: top 20 institutions hosting pharmacologic scholars in the Stanford–Elsevier Lists (SEL) of top scientists worldwide (2017–2023)

Rank	*Career-long* SEL			*Single-year* SEL		
	**University (acronym)**	**Country**	***N* (%)**	**University (acronym)**	**Country**	***N* (%)**
1	University of California (UC)	USA	403 (1.32%)	University of California (UC)	USA	265 (1.06%)
2	University College London (UCL)	GBR	277 (0.91%)	University College London (UCL)	GBR	172 (0.69%)
3	Karolinska Institute (KI)	SWE	239 (0.78%)	University of Toronto (UofT)	CAN	148 (0.59%)
4	University of Texas (UT)	USA	232 (0.76%)	University of North Carolina (UNC)	USA	144 (0.58%)
5	University of Toronto (UofT)	CAN	229 (0.75%)	Springer Nature (Springer)	GBR	140 (0.56%)
6	University of Washington (UW)	USA	204 (0.67%)	University of Texas (UT)	USA	138 (0.55%)
7	University of Nottingham (UoN)	GBR	196 (0.64%)	University of Florida (UF)	USA	133 (0.53%)
8	University of Michigan (UMich)	USA	194 (0.63%)	U.S. Food and Drug Administration (FDA)	USA	126 (0.51%)
9	University of Florida (UF)	USA	192 (0.63%)	Monash University (Monash)	AUS	121 (0.48%)
10	King’s College London (KCL)	GBR	188 (0.62%)	University of Michigan (UMich)	USA	119 (0.48%)
11	University of North Carolina (UNC)	USA	181 (0.59%)	China Pharmaceutical University (CPU)	CHN	112 (0.45%)
12	GlaxoSmithKline (GSK)	GBR	176 (0.58%)	University of Washington (UW)	USA	128 (0.51%)
13	Pfizer (Pfizer)	USA	166 (0.54%)	Tabriz University of Medical Sciences (TUOMS)	IRN	109 (0.44%)
14	University at Buffalo, SUNY (UB)	USA	162 (0.53%)	University of Nottingham (UoN)	GBR	107 (0.43%)
15	Merck & Co. (Merck)	USA	157 (0.51%)	King’s College London (KCL)	GBR	104 (0.42%)
16	University of Arizona (UArizona)	USA	153 (0.50%)	University of Copenhagen (UCPH)	DNK	104 (0.42%)
17	Virginia Commonwealth University (VCU)	USA	140 (0.46%)	Karolinska Institute (KI)	SWE	101 (0.40%)
18	University of Helsinki (UH)	FIN	133 (0.44%)	University of British Columbia (UBC)	CAN	99 (0.40%)
19	Harvard University (HU)	USA	132 (0.43%)	Chinese Academy of Medical Sciences (CAMS)	CHN	99 (0.40%)
20	University of Kentucky (UKY)	USA	128 (0.42%)	University of Naples Federico II (UNINA)	ITA	97 (0.39%)
Total			3882 (12.70%)			2566 (10.28%)

**Table 5:** Institutional-level analyses: correlations between QS, THE, and ARWU scores and the number of excellent pharmacologic scholars hosted by the top 20 institutions in the Stanford–Elsevier Lists (2017–2023)

Database	Indicator	*Career-long* SEL: rho	*Single-year* SEL: rho
Quacquarelli Symonds (QS)	Pharmacy (overall score)	0.204	0.508*
	Pharmacy (academic reputation)	0.500*	0.662**
	General (overall score)	0.429	0.458
	General (academic reputation)	0.608*	0.467
Times Higher Education (THE)	Medicine (overall score)	0.506*	0.374
	Medicine (research quality)	0.356	0.134
	General (overall score)	0.34	0.375
	General (research quality)	0.343	0.289
Academic Ranking of World Universities (ARWU)	Pharmacy (overall score)	0.017	− 0.22
	Pharmacy (research impact)	0.502*	− 0.08
	General (overall score)	0.224	0.704*
	General (per capita performance)	0.500*	0.284

^*^Correlation is significant at the 0.05 level (two-tailed). **Correlation is significant at the 0.01 level (two-tailed)

### Gender-based analyses of pharmacology and pharmacy research excellence

Females comprised 16.8% and 25.0% of EPPS in the *career-long* and *single-year* SEL, respectively. Across income strata, upper-middle-income countries showed the highest female representation (24.4% and 30.1%), exceeding that observed in other World Bank income groups (Table 6).

**Table 6:** Individual-level analysis: gender and academic age of pharmacologic scholars in the Stanford–Elsevier Lists (SEL) of top scientists worldwide (2017–2023)

Variable	Outcome	Female			Male			*p*		
		**Scholars: *N* (%)**	**Citations: median (IQR)**	**Academic age: median (IQR)**	**Scholars: *N* (%)**	**Citations: median (IQR)**	**Academic age: median (IQR)**	**Scholars**	**Citations**	**Age**
*Career-long* SEL										
Year	SEL 2017	363 (13.0%)	5673 (3946–8683)	32 (25–38)	2431 (87.0%)	6655 (4207.5–10,887)	36 (29–43)	**< 0.001**	**< 0.001**	** < 0.001**
	SEL 2018	351 (13.3%)	6355 (4398–9323.5)	32 (26–39)	2284 (86.7%)	7639 (4930–12,384.5)	38 (31–45)		**< 0.001**	**< 0.001**
	SEL 2019	582 (16.5%)	5114 (3186–7850)	30 (24–37)	2937 (83.5%)	6037 (3707–10,219)	36 (29–43)		**< 0.001**	**< 0.001**
	SEL 2020	787 (17.4%)	5136 (3203.5–8113.5)	31 (24–38)	3727 (82.6%)	5858 (3461–10,080.5)	37 (29–44)		**< 0.001**	**< 0.001**
	SEL 2021	817 (17.6%)	5244 (3163–8266)	32 (25–38)	3828 (82.4%)	5955 (3468–10,303.5)	37 (30–44)		** < 0.001**	**< 0.001**
	SEL 2022	875 (18.1%)	5530 (3386–8830)	32 (26–39)	3957 (81.9%)	6386 (3703–10,858)	38 (30–45)		**< 0.001**	**< 0.001**
	SEL 2023	949 (18.6%)	5769 (3494–9273)	32 (26–39)	4152 (81.4%)	6640 (3811.5–11,260.5)	38 (30–45)		**< 0.001**	**< 0.001**
World Bank	High	4203 (16.4%)	5628 (3544–8891.5)	32 (25–39)	21,414 (83.6%)	6498 (3889–10,970.2)	38 (30–44)	**< 0.001**	**< 0.001**	**< 0.001**
	Upper-middle	306 (24.4%)	4848.5 (3071.2–7475.8)	27 (22–32)	946 (75.6%)	6428 (3612.2–10,087.8)	28 (21–35)		**< 0.001**	0.290
	Lower-middle	133 (20.0%)	2911 (2227–4488)	28 (21–34)	531 (80.0%)	3874 (2601.5–6128)	28 (20.5–38)		**< 0.001**	0.379
	Low	0 (0.0%)	NA	NA	6 (100.0%)	3656 (2930.2–4174.8)	27 (19–29.8)		*NA*	*NA*
WHO region	EURO	1907 (17.0%)	5745 (3480–8695)	32 (26–39)	9332 (83.0%)	6851.5 (4096–11,400.5)	37 (31–44)	**0.017**	**< 0.001**	**< 0.001**
	AMRO	1852 (16.7%)	5679 (3606.5–9465.2)	33 (26–40)	9253 (83.3%)	6272 (3690–10,886)	38 (31–46)		** < 0.001**	**< 0.001**
	WPRO	676 (16.7%)	4959.5 (3365–7536)	27 (19–34)	3368 (83.3%)	6580 (4058.5–10,250.2)	35 (27–42)		**< 0.001**	**< 0.001**
	EMRO	89 (15.2%)	2987 (2155–4866)	23 (17–27)	497 (84.8%)	3547 (2325–5693)	24 (17–31)		0.076	0.095
	SEARO	109 (22.4%)	3799 (2588–5575)	30 (24–35)	378 (77.6%)	4958.5 (3078.8–7052.5)	26 (21–39)		**< 0.001**	0.409
	AFRO	9 (11.5%)	2259 (2036–4660)	26 (23–27)	69 (88.5%)	2965 (2245–3538)	31 (20–38)		0.568	0.471
Official language	English	2605 (17.3%)	5538 (3488–9060)	32 (25–39)	12,437 (82.7%)	6294 (3735–10,836)	38 (31–45)	**< 0.001**	** < 0.001**	**< 0.001**
	German	326 (14.2%)	5893.5 (3468.2–8227.5)	29.5 (24–36)	1962 (85.8%)	6922 (3773.5–11,256)	36 (29–43.8)		**< 0.001**	** < 0.001**
	Japanese	105 (6.1%)	4610 (3782–6195)	34 (30–42)	1625 (93.9%)	6601 (4213–9991)	39 (32–44)		**< 0.001**	**0.005**
	Italian	281 (23.2%)	7226 (5203–9696)	37 (29–41)	930 (76.8%)	7850.5 (5056.5–12,905.8)	39 (33–45)		**0.001**	**< 0.001**
	Chinese	137 (24.5%)	6676 (4869–9815)	27 (21–31)	423 (75.5%)	8246 (5358–11,873)	28 (22–34)		**0.002**	**0.035**
	Other	1270 (17.6%)	4750.5 (2953.5–7825.2)	31 (25–37)	5939 (82.4%)	6020 (3624.5–10,277)	35 (27–42)		** < 0.001**	**< 0.001**
Total		4724 (16.8%)	5481 (3411.5–8680.2)	31 (25–38)	23,316 (83.2%)	6391.5 (3821.8–10,787.2)	37 (30–44)		**< 0.001**	**< 0.001**
*Single-year* SEL										
Year	SEL 2017	332 (17.3%)	535.5 (358–785.8)	NA	1583 (82.7%)	600 (394–973.5)	NA	**< 0.001**	** < 0.001**	*NA*
	SEL 2019	786 (24.2%)	592.5 (385–902)	23 (17–30)	2465 (75.8%)	656 (409–1083)	30 (21–39)		**< 0.001**	**< 0.001**
	SEL 2020	1099 (25.4%)	713 (479.5–1152)	23 (16–30.5)	3232 (74.6%)	787 (480–1305)	29 (19–39)		**0.001**	**< 0.001**
	SEL 2021	1138 (25.7%)	493.5 (329.2–762.8)	23 (16–31)	3291 (74.3%)	544 (341–913)	29 (18–39)		**< 0.001**	**< 0.001**
	SEL 2022	1209 (26.1%)	513 (346–807)	23 (16–31)	3426 (73.9%)	571 (364–957)	28 (18–39)		**< 0.001**	**< 0.001**
	SEL 2023	1291 (26.6%)	486 (326.5–795.5)	22 (16–30.5)	3562 (73.4%)	555 (347.2–941)	27 (17–38.8)		**< 0.001**	**< 0.001**
World Bank	High	4330 (24.3%)	567 (370–903)	25 (18–32)	13,504 (75.7%)	616 (382–1051)	32 (23–41)	**< 0.001**	**< 0.001**	** < 0.001**
	Upper-middle	993 (30.1%)	557 (379–926)	18 (14–25)	2302 (69.9%)	708.5 (455–1129)	18 (13–25)		**< 0.001**	0.454
	Lower-middle	466 (24.0%)	382 (264.2–565.5)	16 (13–23)	1478 (76.0%)	468.5 (303–749.8)	17 (12–25)		**< 0.001**	0.259
	Low	1 (6.2%)	75 (75–75)	13 (13–13)	15 (93.8%)	489 (246–1380)	7 (3.5–12.5)		0.193	0.514
WHO region	EURO	2226 (27.3%)	566.5 (376.2–872.5)	26 (18–32)	5935 (72.7%)	684 (429–1169)	33 (24–41)	**< 0.001**	**< 0.001**	**< 0.001**
	AMRO	1608 (23.1%)	563 (363–960.2)	25 (19–34)	5344 (76.9%)	568.5 (346–973.2)	34 (24–43)		0.824	**< 0.001**
	WPRO	1151 (25.9%)	601 (395–969)	19 (15–26)	3290 (74.1%)	679 (435–1112)	24 (17–34)		**< 0.001**	** < 0.001**
	EMRO	483 (23.7%)	383 (254–593.5)	15 (11–19)	1559 (76.3%)	498 (320–800.5)	15 (11–22)		**< 0.001**	0.128
	SEARO	308 (22.4%)	449 (315.2–625.8)	19 (14–28)	1070 (77.6%)	506.5 (331.2–791.5)	18 (13–25)		**< 0.001**	0.293
	AFRO	14 (12.2%)	602 (370.2–984.8)	21 (17–24)	101 (87.8%)	311 (182–622)	15 (12–22.5)		**0.05**	**0.038**
Official language	English	2283 (24.0%)	563 (355.5–942.5)	25 (18–33)	7227 (76.0%)	586 (354–1010)	34 (24–43)	**< 0.001**	**0.021**	**< 0.001**
	German	290 (19.2%)	556 (375.5–893.8)	25 (18–30.5)	1220 (80.8%)	621 (374.8–1001.8)	32 (25–39)		0.087	** < 0.001**
	Japanese	57 (6.3%)	486 (356–754)	32 (26–38)	854 (93.7%)	546 (369.2–887.5)	35 (25–41)		0.155	0.159
	Italian	507 (38.5%)	606 (430.5–880.5)	26 (19–35)	810 (61.5%)	812 (509.2–1374.8)	33 (22–41)		**< 0.001**	**< 0.001**
	Chinese	499 (29.8%)	697 (476–1172.5)	18 (15–24)	1175 (70.2%)	814 (523–1249)	19 (15–25)		**0.003**	0.273
	Other	2219 (26.1%)	482 (317–760.5)	20 (14–29)	6273 (73.9%)	595 (378–980)	23 (15–33)		**< 0.001**	** < 0.001**
Total		5855 (25%)	549 (359–870.5)	23 (16–31)	17,559 (75%)	612 (382–1028)	28 (18–39)		**< 0.001**	**< 0.001**

Chi-squared (*χ*^*2*^) test, Fisher’s exact test, and Mann–Whitney (*U*) test were used with a significance level *p* ≤ 0.05

Values in bold refer to statistical significance (*p*. <0.05)

At the country level (restricted to countries with > 50 EPPS), the *career-long* SEL showed the largest female shares in Turkey (60.2%) and New Zealand (53.2%), whereas Malaysia (0%) and Saudi Arabia (1.0%) ranked lowest. In the *single-year* SEL, Norway (64.9%) and Turkey (59.8%) were highest, while Japan (6.3%) and Saudi Arabia (8.6%) were lowest (Tables S3–S4).

Gender-based comparisons indicated higher male performance on several metrics. Median citation counts were greater among males in both the *career-long* (6391.5 vs. 5481; *p* < 0.001) and *single-year* (612 vs. 549; *p* < 0.001) SEL. Median academic age was also higher in males (37 vs. 31 years, *p* < 0.001, and 28 vs. 23 years, *p* < 0.001, respectively) (Table 6). Core bibliometric outcomes showed the same pattern, with higher modified *H*-index values for males in the *career-long* (18.4 vs. 16.8, *p* < 0.001) and *single-year* (5.6 vs. 5.1, *p* < 0.001) SEL (Table S5).

### Age-based analyses of pharmacology and pharmacy research excellence

The median academic age of EPPS was 36 years (IQR 29–43) in the *career-long* SEL and 27 years (IQR 18–37) in the *single-year* SEL. In the *career-long* dataset, Hungary reported the longest academic age (45 years (37–54)), while New Zealand had the shortest (19 years (12–31)). In the *single-year* SEL, Israel reported the highest (39 years (26–46)), and Iraq the lowest (10 years (8–13)) (Tables S6–S7).

**Fig. 3:**
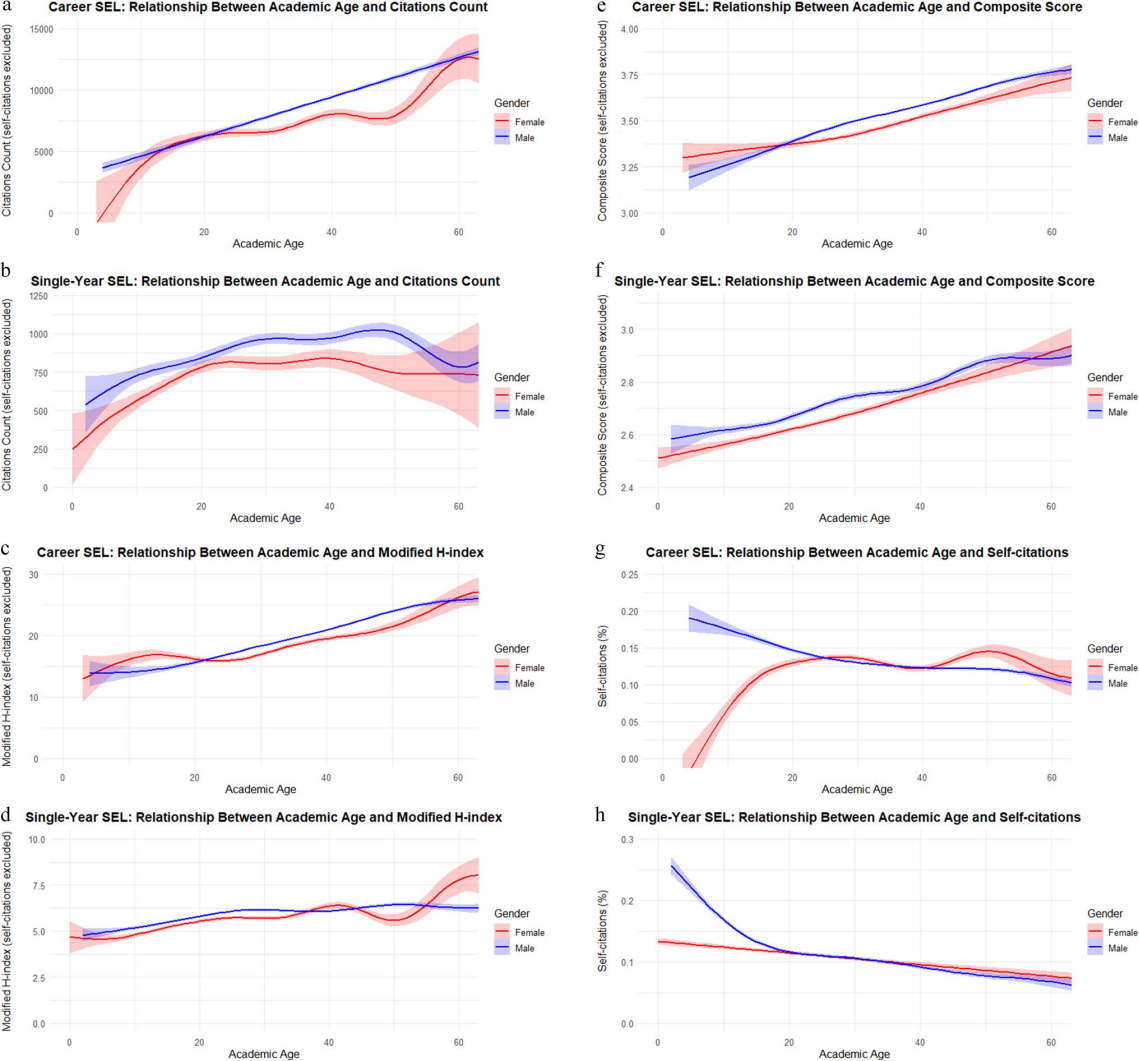
Gender-stratified associations between academic age and scholarly output metrics among excellent pharmacology and pharmacy scholars (EPPS) in the Stanford–Elsevier Top 2% Lists (2017–2023): **a**, **b** citation count; **c**, **d** composite score; **e**, **f** modified *H*-index; and **g**, **h** percentage of self-citations for *career-long* and *single-year* lists, respectively

Correlations with core bibliometric outcomes indicated that the modified *H*-index showed the strongest correlation coefficients in the *career-long* SEL (*ρ* = 0.330), while the composite score was strongest in the *single-year* SEL (*ρ* = 0.243). In both datasets, self-citations were negatively correlated with academic age (*ρ* = − 0.072 and − 0.223) (Fig. 3).

For secondary bibliometric outcomes, correlations were consistently stronger in the *single-year* SEL than in the *career-long* SEL. Notably, academic age correlated with the number of single-authored publications (*ρ* = 0.586 vs. 0.395) and with the number of single-, first-, and last-authored publications (*ρ* = 0.682 vs. 0.467) (Table S8).

### Disciplinary classifications

**Fig. 4:**
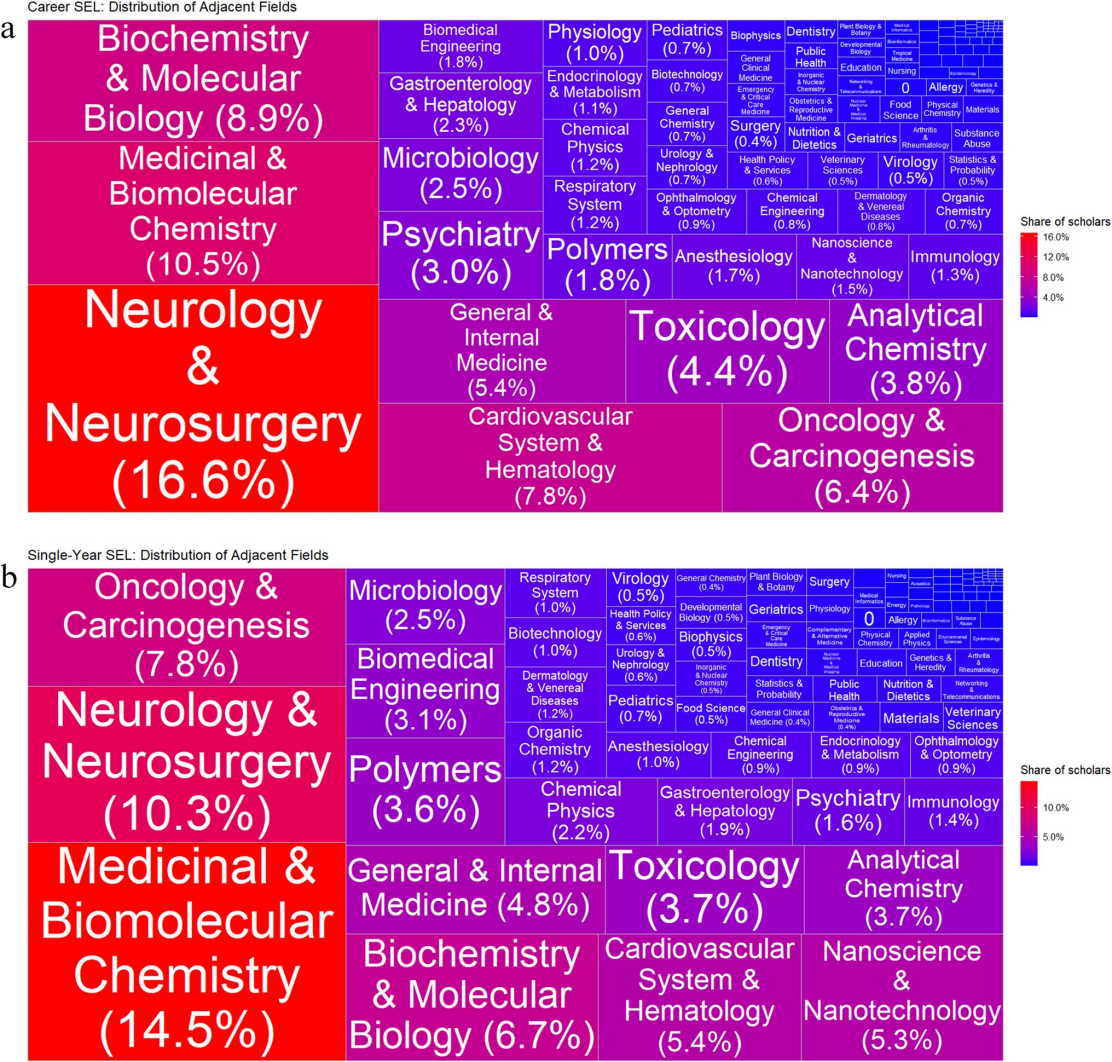
Treemap chart of adjacent sub-fields of excellent pharmacology and pharmacy scholars (EPPS) in the Stanford–Elsevier Top 2% Lists (2017–2023): **a** *career-long* and **b** *single-year* lists

The disciplinary classification of *pharmacology and pharmacy* was assigned as the primary subfield in 54.2% of EPPS records and as the secondary subfield in 45.8%. The adjacent subfields are presented in Fig. 4.

### Time-trend analyses of pharmacology and pharmacy research excellence

Between 2017 and 2023, the share of EPPS from high-income countries decreased substantially (− 7.1% and − 22.14% in the *career-long* and *single-year* SEL, respectively). Regionally, EURO (− 2.42% and − 8.67%) and AMRO − 7.2% and − 14.29%) declined, while other regions, particularly EMRO, expanded (+ 2.42% and + 12.20%) (Table 1). Female representation increased from 13.0 to 18.6% in the *career-long* SEL and from 17.3 to 26.6% in the *single-year* SEL (Table 6). Geographic coverage also widened, with 87 countries represented in the *career-long* SEL compared to 96 in the *single-year* SEL (Table S1–S2).

### Determinants of female representation among excellent pharmacologic scholars

Female group membership was associated with a younger academic age (*career-long* OR = 0.951; *single-year* OR = 0.965). Compared with high-income countries, women were more likely to be represented in upper-middle-income countries (OR = 1.648; 1.345). Gender inequality was positively associated with female membership in the *career-long* SEL but negatively associated in the *single-year* SEL (OR = 1.448; 0.742). The Human Development Index remained a consistent negative predictor (OR = 0.184; 0.678) (Table 7).

**Table 7:** Individual-level analysis: logistic regression models for female gender (group membership) among pharmacologic scholars in the Stanford–Elsevier Lists (SEL) of top scientists worldwide (2017–2023)

Group	Predictor	*Career-long* SEL		*Single-year* SEL	
		**OR (CI 95%)**	***p***	**OR (CI 95%)**	***p***
Individual	Academic age (*per year*)	0.951 (0.948–0.954)	** < 0.001**	0.965 (0.962–0.967)	** < 0.001**
World Bank	World Bank: low income vs. high income	0.000 (0.000–Inf)	0.902	0.208 (0.028–1.566)	0.127
	World Bank: lower-middle income vs. high income	1.276 (1.052–1.548)	**0.013**	0.983 (0.881–1.097)	0.763
	World Bank: upper-middle income vs. high income	1.648 (1.443–1.883)	** < 0.001**	1.345 (1.239–1.460)	** < 0.001**
WHO region	WHO region: AFRO vs. AMRO	0.652 (0.325–1.308)	0.228	0.461 (0.263–0.808)	**0.007**
	WHO region: EMRO vs. AMRO	0.895 (0.710–1.127)	0.345	1.030 (0.917–1.157)	0.623
	WHO region: EURO vs. AMRO	1.021 (0.952–1.095)	0.562	1.246 (1.158–1.342)	** < 0.001**
	WHO region: SEARO vs. AMRO	1.441 (1.158–1.793)	**0.001**	0.957 (0.833–1.099)	0.530
	WHO region: WPRO vs. AMRO	1.003 (0.911–1.104)	0.955	1.163 (1.066–1.269)	** < 0.001**
Language	English-speaking: No vs. Yes	0.930 (0.873–0.990)	**0.023**	1.094 (1.030–1.163)	**0.003**
Health system	Universal Health Coverage Index (UHC)	0.994 (0.984–1.003)	0.188	1.014 (1.008–1.020)	** < 0.001**
	General government expenditure on health (%)	0.986 (0.981–0.991)	** < 0.001**	0.987 (0.983–0.991)	** < 0.001**
	Number of pharmacists per capita	0.969 (0.961–0.976)	** < 0.001**	0.984 (0.977–0.992)	** < 0.001**
Gender equity	Gender Inequality Index (GII)	1.448 (1.031–2.035)	**0.033**	0.742 (0.588–0.935)	**0.012**
	Maternal mortality per 100 K live births	1.000 (0.999–1.001)	0.554	0.998 (0.997–0.999)	** < 0.001**
	Adolescent birth rate (*per* 1 K women aged 15–19)	1.006 (1.002–1.010)	**0.003**	1.001 (0.999–1.004)	0.314
	Education gap 25 + (male − female)	1.039 (1.031–1.048)	** < 0.001**	1.013 (1.007–1.018)	** < 0.001**
	Employment gap 15 + (male − female)	1.005 (1.001–1.008)	**0.022**	0.999 (0.997–1.001)	0.449
	Female share of parliamentary seats (%)	1.012 (1.008–1.016)	** < 0.001**	1.013 (1.010–1.016)	** < 0.001**
Human development	Human Development Index (HDI)	0.184 (0.106–0.321)	** < 0.001**	0.678 (0.477–0.963)	**0.030**
	Life expectancy at birth (years)	0.977 (0.966–0.988)	** < 0.001**	1.004 (0.995–1.012)	0.380
	Expected years of schooling	0.925 (0.908–0.942)	** < 0.001**	0.960 (0.948–0.972)	** < 0.001**
	Mean years of schooling	1.051 (1.029–1.073)	** < 0.001**	1.058 (1.041–1.076)	** < 0.001**
	Gross national income per capita (USD)	1.000 (1.000–1.000)	** < 0.001**	1.000 (1.000–1.000)	** < 0.001**
Budgetary policies	GDP spent on research and development (*per %*)	0.829 (0.804–0.856)	** < 0.001**	0.880 (0.858–0.901)	** < 0.001**
	GDP spent on education (*per %*)	0.979 (0.944–1.015)	0.243	0.955 (0.925–0.986)	**0.005**
	GDP spent on health (*per %*)	0.968 (0.960–0.976)	** < 0.001**	0.974 (0.967–0.980)	** < 0.001**

Values in bold refer to statistical significance (*p*. <0.05)

### Effects of academic age on bibliometric outcomes

Academic age emerged as a robust predictor of core bibliometric outcomes. On average, each additional year corresponded to an increase in citation counts (*career-long β* = 152.0; *single-year β* = 6.5), modified *H*-index (*β* = 0.246; 0.028), and composite score (*β* = 0.009; 0.006). The associations did not differ materially between male and female EPPS. Secondary bibliometric measures also displayed comparable positive relationships with academic age (Table 8).

**Table 8:** Individual-level analyses: linear regression models of scholarly output metrics based on academic age “predictor” of pharmacologic scholars in the Stanford–Elsevier Lists (2017–2023)

Scholarly output metric	Overall: *β* (95% CI); *p*	Female:* β* (95% CI); *p*	Male: *β* (95% CI); *p*
*Career-long* SEL			
Total citations^╪^	151.957 (142.801–161.113); < **0.001**	91.851 (75.244–108.458); < **0.001**	160.661 (149.138–172.183); < **0.001**
Modified *H*-index^╪^	0.246 (0.238–0.254); < **0.001**	0.176 (0.158–0.195); < **0.001**	0.261 (0.251–0.271); < **0.001**
Composite score^╪^	0.009 (0.009–0.010); < **0.001**	0.008 (0.007–0.009); < **0.001**	0.009 (0.009–0.010); < **0.001**
Self-citations (%)	− 0.001 (− 0.001 to − 0.001); < **0.001**	0.000 (0.000–0.001); < **0.001**	− 0.001 (− 0.001 to − 0.001); < **0.001**
Total papers	4.935 (4.779–5.091); < **0.001**	2.846 (2.537–3.156); < **0.001**	5.142 (4.951–5.333); < **0.001**
Single-authored papers (number)	0.568 (0.539–0.598); < **0.001**	0.233 (0.100–0.366); < **0.001**	0.628 (0.598–0.658); < **0.001**
Single-authored papers (citations)^╪^	11.764 (10.964–12.564); < **0.001**	8.568 (7.000–10.135); < **0.001**	12.964 (11.961–13.968); < **0.001**
Single- and first-authored papers (number)	1.385 (1.337–1.433); < **0.001**	0.812 (0.657–0.966); < **0.001**	1.455 (1.400–1.509); < **0.001**
Single- and first-authored papers (citations)^╪^	15.183 (13.222–17.144); < **0.001**	4.676 (0.749–8.602); **0.020**	17.465 (15.021–19.910); < **0.001**
Single-, first-, and last-authored papers (number)	4.060 (3.958–4.162); < **0.001**	2.244 (2.026–2.463); < **0.001**	4.264 (4.141–4.388); < **0.001**
Single-, first-, and last-authored papers (citations)^╪^	108.131 (102.845–113.417); < **0.001**	68.416 (59.711–77.120); < **0.001**	116.038 (109.368–122.707); < **0.001**
*Single-year* SEL			
Total citations^╪^	6.521 (5.522–7.520); < **0.001**	6.812 (4.723–8.901); < **0.001**	5.897 (4.643–7.151); < **0.001**
Modified *H*-index^╪^	0.028 (0.026–0.030); < **0.001**	0.038 (0.033–0.042); < **0.001**	0.025 (0.022–0.027); < **0.001**
Composite score^╪^	0.006 (0.006–0.007); < **0.001**	0.007 (0.006–0.007); < **0.001**	0.006 (0.006–0.007); < **0.001**
Self-citations (%)	− 0.002 (− 0.002 to − 0.002); < **0.001**	− 0.001 (− 0.001 to − 0.001); < **0.001**	− 0.002 (− 0.002 to − 0.002); < **0.001**
Total papers	6.642 (6.504–6.781); < **0.001**	4.744 (4.507–4.981); < **0.001**	6.776 (6.601–6.950); < **0.001**
Single-authored papers (number)	0.654 (0.626–0.683); < **0.001**	0.434 (0.331–0.537); < **0.001**	0.698 (0.671–0.725); < **0.001**
Single-authored papers (citations) ^╪^	0.780 (0.712–0.848); < **0.001**	0.409 (0.279–0.539); < **0.001**	0.914 (0.831–0.996); < **0.001**
Single- and first-authored papers (number)	1.565 (1.524–1.606); < **0.001**	1.098 (0.982–1.213); < **0.001**	1.620 (1.574–1.666); < **0.001**
Single- and first-authored papers (citations) ^╪^	− 0.316 (− 0.471 to − 0.161); < **0.001**	− 1.368 (− 1.663 to − 1.073); < **0.001**	− 0.102 (− 0.296 to 0.092); 0.303
Single-, first-, and last-authored papers (number)	4.678 (4.590–4.765); < **0.001**	3.136 (2.973–3.300); < **0.001**	4.829 (4.720–4.937); < **0.001**
Single-, first-, and last-authored papers (citations)^╪^	4.884 (4.496–5.272); < **0.001**	4.606 (3.929–5.284); < **0.001**	4.734 (4.237–5.230); < **0.001**

^╪^Self-citations were excluded

Values in bold refer to statistical significance (*p*. <0.05)

### Regression analyses of pharmacology and pharmacy research excellence

Two complementary regression strategies were adopted to assess predictors of *research excellence*. The first strategy assessed each national-level determinant in turn, adjusting for gender and academic age. The second strategy integrated both individual-level predictors and broader thematic indicators (World Bank income group, WHO region, language status, GII, HDI, and UHC) within a single multivariable framework.

In the first strategy, stronger health system performance and development indicators were positively related to citation counts. Specifically, higher UHC scores (adj. *β* = 153; 11), HDI values (adj. *β* = 7616; 438), and greater national investment in research and development (adj. *β* = 293; 5), education (adj. *β* = 236; 15.6), and health (adj. *β* = 70; 0.18) were all associated with increased citations. In contrast, greater gender inequality predicted fewer citations (adj. *β* = − 4898; − 437) (Table 9).

**Table 9:** Individual-level analysis: linear regression of scholarly outputs of pharmacologic scholars in the Stanford–Elsevier Lists (SEL) of top scientists worldwide (2017–2023)

		*Career–Long* SEL							
		Citation count		Modified *H*-index		Composite score (*C*)		% Self-citations	
Individual-level determinants		*β* (95% CI)	*p*	*β* (95% CI)	*p*	*β* (95% CI)	*p*	*β* (95% CI)	*p*
	Gender (male vs. female)	1999 (1714–2284)	**< 0.001**	2.379 (2.119–2.638)	** < 0.001**	0.104 (0.093–0.115)	** < 0.001**	− 0.002 (− 0.004 to − 0.000)	**0.048**
	Academic Age (*per* year)	152 (143–161)	**< 0.001**	0.246 (0.238–0.254)	**< 0.001**	0.009 (0.009–0.010)	**< 0.001**	− 0.001 (− 0.001 to − 0.001)	** < 0.001**
National-level determinants		Adj. β (95% CI)	*p*	Adj. β (95% CI)	*p*	Adj. β (95% CI)	*p*	Adj. β (95% CI)	*p*
World Bank	Low income vs. high income	− 3090 (− 10,289 to 4109)	0.400	− 3.548 (− 9.863 to 2.767)	0.271	− 0.051 (− 0.311 to 0.209)	0.698	− 0.036 (− 0.091 to 0.019)	0.196
	Lower-middle income vs. high income	− 2821 (− 3518 to − 2123)	**< 0.001**	− 0.558 (− 1.170 to 0.054)	0.074	− 0.159 (− 0.185 to − 0.134)	**< 0.001**	0.024 (0.019–0.030)	**< 0.001**
	Upper-middle income vs. high income	− 163 (− 680 to 354)	0.537	0.777 (0.323–1.231)	**< 0.001**	− 0.125 (− 0.144 to − 0.107)	** < 0.001**	0.041 (0.038–0.045)	**< 0.001**
WHO Region	EURO vs. AMRO	179 (− 57 to 415)	0.136	− 0.146 (− 0.353 to 0.061)	0.167	− 0.029 (− 0.037 to − 0.020)	**< 0.001**	0.021 (0.019–0.023)	** < 0.001**
	WPRO vs. AMRO	− 347 (− 673 to − 21)	**0.037**	0.379 (0.092–0.665)	**0.010**	− 0.106 (− 0.118 to − 0.094)	**< 0.001**	0.025 (0.023–0.028)	**< 0.001**
	EMRO vs. AMRO	− 2171 (− 2930 to − 1413)	**< 0.001**	− 0.226 (− 0.892 to 0.439)	0.506	− 0.165 (− 0.193 to − 0.138)	**< 0.001**	0.078 (0.072–0.084)	**< 0.001**
	AFRO vs. AMRO	− 4894 (− 6898 to − 2891)	**< 0.001**	− 3.066 (− 4.825 to − 1.308)	**< 0.001**	− 0.154 (− 0.226 to − 0.082)	**< 0.001**	0.003 (− 0.013 to 0.018)	0.743
	SEARO vs. AMRO	− 2382 (− 3202 to − 1563)	**< 0.001**	0.188 (− 0.531 to 0.907)	0.608	− 0.186 (− 0.215 to − 0.156)	**< 0.001**	0.022 (0.016–0.028)	**< 0.001**
Language	English-speaking: no vs. yes	− 93 (− 304 to 119)	0.390	− 0.436 (− 0.622 to − 0.251)	**< 0.001**	− 0.075 (− 0.082 to − 0.067)	**< 0.001**	0.033 (0.032–0.035)	**< 0.001**
Health System	Universal Health Coverage Index (UHC)	153 (119–187)	**< 0.001**	0.036 (0.007–0.066)	**0.016**	0.008 (0.007–0.009)	** < 0.001**	− 0.002 (− 0.002 to − 0.002)	**< 0.001**
	General Government Expenditure on Health (%)	21 (4–38)	**0.015**	− 0.001 (− 0.016 to 0.014)	0.916	0.003 (0.003–0.004)	**< 0.001**	− 0.002 (− 0.002 to − 0.002)	**< 0.001**
	Number of pharmacists per capita	− 72 (− 97 to − 48)	**< 0.001**	− 0.073 (− 0.095 to − 0.051)	**< 0.001**	− 0.003 (− 0.004 to − 0.002)	**< 0.001**	7.336e − 04 (5.236e − 04–9.436e − 04)	**< 0.001**
Gender Equity	Gender Inequality Index (*per n*)	− 4898 (− 6072 to − 3725)	** < 0.001**	− 1.160 (− 2.190 to − 0.131)	**0.027**	− 0.129 (− 0.171 to − 0.086)	**< 0.001**	0.016 (0.007–0.025)	**< 0.001**
	Maternal mortality rate (*per n*)	− 8 (− 11 to − 6)	**< 0.001**	− 0.004 (− 0.006 to − 0.001)	**0.002**	− 3.431e − 04 (− 4.461e − 04 to − 2.400e − 04)	**< 0.001**	1.227e − 05 (− 9.476e − 06 to 3.401e − 05)	0.269
	Adolescent birth rate (*per n*)	− 58 (− 72 to − 44)	**< 0.001**	− 0.024 (− 0.036 to − 0.011)	** < 0.001**	− 3.914e − 04 (− 8.904e − 04 to 1.076e − 04)	0.124	9.477e − 05 (− 1.045e − 05 to 2.000e − 04)	0.077
	Employment gap (male − female) (*per %*)	− 53 (− 67 to − 39)	**< 0.001**	− 0.012 (− 0.024 to 0.000)	0.053	− 0.005 (− 0.006 to − 0.005)	**< 0.001**	0.002 (0.002–0.002)	**< 0.001**
	Education gap (male − female) (*per %*)	− 22 (− 52 to 9)	0.172	0.018 (− 0.009 to 0.045)	0.189	− 0.011 (− 0.012 to − 0.010)	**< 0.001**	0.003 (0.003–0.003)	**< 0.001**
	Female share of parliamentary seats (*per %*)	39 (26–51)	**< 0.001**	0.030 (0.019–0.041)	**< 0.001**	0.003 (0.003–0.004)	** < 0.001**	− 7.484e − 04 (− 8.461e − 04 to − 6.508e − 04)	**< 0.001**
Human development	Human Development Index (*per n*)	7616 (5527–9706)	**< 0.001**	2.159 (0.326–3.992)	**0.021**	0.716 (0.640–0.791)	**< 0.001**	− 0.206 (− 0.222 to − 0.191)	**< 0.001**
	Gross national income per capita (*per USD*)	0.018 (0.011–0.025)	**< 0.001**	1.183e − 06 (− 4.551e − 06 to 6.917e − 06)	0.686	2.302e − 06 (2.066e − 06–2.537e − 06)	**< 0.001**	− 8.671e − 07 (− 9.160e − 07 to − 8.181e − 07)	**< 0.001**
	Expected years of schooling (*per year*)	130 (59–202)	**< 0.001**	0.178 (0.115–0.241)	**< 0.001**	0.008 (0.006–0.011)	**< 0.001**	− 0.002 (− 0.003 to − 0.002)	**< 0.001**
	Mean years of schooling (*per year*)	71 (3–139)	**0.042**	0.039 (− 0.021 to 0.099)	0.205	0.027 (0.024–0.029)	**< 0.001**	− 0.008 (− 0.009 to − 0.007)	**< 0.001**
	Life expectancy at birth (*per year*)	165 (127–204)	**< 0.001**	0.035 (0.001–0.069)	**0.044**	0.002 (0.001–0.003)	**0.005**	9.711e − 05 (− 1.991e − 04 to 3.933e − 04)	0.520
Budget. policies	GDP spent on research (*per %*)	293 (185–401)	**< 0.001**	0.065 (− 0.029 to 0.160)	0.176	0.022 (0.018–0.026)	**< 0.001**	− 0.012 (− 0.013 to − 0.011)	**< 0.001**
	GDP spent on education (*per %*)	236 (113–358)	**< 0.001**	0.125 (0.017–0.232)	**0.023**	0.034 (0.029–0.038)	**< 0.001**	− 0.014 (− 0.015 to − 0.013)	**< 0.001**
	GDP spent on health (*per %*)	70 (41–99)	**< 0.001**	− 0.006 (− 0.032 to 0.019)	0.620	0.011 (0.010–0.012)	**< 0.001**	− 0.004 (− 0.004 to − 0.004)	**< 0.001**
Disease burden	DALYs attributed to COPD	0.361 (0.071–0.651)	**0.015**	4.970e − 04 (2.432e − 04–7.509e − 04)	**< 0.001**	6.704e − 05 (5.655e − 05–7.752e − 05)	**< 0.001**	− 3.190e − 05 (− 3.408e − 05 to − 2.971e − 05)	**< 0.001**
	DALYs attributed to diabetes mellitus	− 0.469 (− 0.798 to − 0.140)	**0.005**	− 6.175e − 04 (− 9.055e − 04 to − 3.295e − 04)	**< 0.001**	1.582e − 05 (3.885e − 06–2.775e − 05)	**0.009**	− 1.298e − 05 (− 1.549e − 05 to − 1.047e − 05)	**< 0.001**
	DALYs attributed to HIV/AIDS	− 0.512 (− 0.780 to − 0.245)	**< 0.001**	− 3.131e − 04 (− 5.474e − 04 to − 7.871e − 05)	**0.009**	− 5.431e − 06 (− 1.514e − 05 to 4.276e − 06)	0.273	− 4.297e − 06 (− 6.345e − 06 to − 2.249e − 06)	** < 0.001**
	DALYs attributed to ischemic heart disease	− 0.673 (− 0.825 to − 0.521)	** < 0.001**	− 3.628e − 04 (− 4.960e − 04 to − 2.295e − 04)	**< 0.001**	4.492e − 06 (− 1.029e − 06 to 1.001e − 05)	0.111	4.464e − 06 (3.300e − 06–5.628e − 06)	**< 0.001**
	DALYs attributed to neoplasms	0.283 (0.175–0.390)	**< 0.001**	− 2.387e − 04 (− 3.331e − 04 to − 1.443e − 04)	**< 0.001**	8.227e − 06 (4.318e − 06–1.214e − 05)	**< 0.001**	4.525e − 06 (3.701e − 06–5.348e − 06)	**< 0.001**
	DALYs attributed to stroke	− 0.249 (− 0.464 to − 0.035)	**0.023**	− 3.734e − 04 (− 5.610e − 04 to − 1.858e − 04)	**< 0.001**	− 6.948e − 05 (− 7.721e − 05 to − 6.175e − 05)	**< 0.001**	2.351e − 05 (2.189e − 05–2.513e − 05)	**< 0.001**
		*Single-year* SEL							
		Citation count		Modified *H*-index		Composite score (*C*)		% Self-citations	
Individual-level determinants		*β* (95% CI)	*p*	*β (*95% CI)	*p*	*β* (95% CI)	*p*	*β* (95% CI)	*p*
	Gender (male vs. female)	147 (118–177)	**< 0.001**	0.391 (0.329–0.453)	**< 0.001**	0.093 (0.083–0.104)	**< 0.001**	0.000 (− 0.002 to 0.003)	0.812
	Academic age (*per year*)	7 (6–8)	**< 0.001**	0.028 (0.026–0.030)	** < 0.001**	0.006 (0.006–0.007)	**< 0.001**	− 0.002 (− 0.002 to − 0.002)	**< 0.001**
National-level determinants		Adj. *β* (95% CI)	*p*	Adj. *β* (95% CI)	*p*	Adj. *β* (95% CI)	*p*	Adj. *β* (95% CI)	*p*
World Bank	Low income vs. high income	241 (− 255 to 738)	0.341	− 1.540 (− 2.564 to − 0.517)	**0.003**	− 0.121 (− 0.278 to 0.037)	0.134	− 0.055 (− 0.097 to − 0.014)	**0.009**
	Lower-middle income vs. high income	− 239 (− 288 to − 189)	**< 0.001**	0.117 (0.015–0.220)	**0.025**	− 0.084 (− 0.100 to − 0.069)	**< 0.001**	0.015 (0.011–0.019)	**< 0.001**
	Upper-middle income vs. high income	83 (43–123)	**< 0.001**	0.321 (0.239–0.404)	**< 0.001**	− 0.072 (− 0.084 to − 0.059)	**< 0.001**	0.004 (0.001–0.008)	**0.012**
WHO region	EURO vs. AMRO	129 (95–163)	**< 0.001**	0.156 (0.085–0.226)	**< 0.001**	− 0.010 (− 0.021–0.001)	0.065	0.025 (0.022–0.028)	** < 0.001**
	WPRO vs. AMRO	83 (43–124)	**< 0.001**	0.277 (0.194–0.361)	**< 0.001**	− 0.072 (− 0.085 to − 0.059)	**< 0.001**	− 0.000 (− 0.003 to 0.003)	0.981
	EMRO vs. AMRO	− 63 (− 117 to − 9)	**0.022**	0.397 (0.286–0.508)	** < 0.001**	− 0.052 (− 0.069 to − 0.035)	**< 0.001**	0.053 (0.049–0.058)	**< 0.001**
	AFRO vs. AMRO	− 219 (− 409 to − 30)	**0.023**	− 0.316 (− 0.707 to 0.075)	0.113	− 0.094 (− 0.154 to − 0.034)	**0.002**	− 0.002 (− 0.017 to 0.014)	0.838
	SEARO vs. AMRO	− 168 (− 229 to − 107)	** < 0.001**	0.337 (0.211–0.463)	**< 0.001**	− 0.104 (− 0.123 to − 0.084)	**< 0.001**	0.012 (0.007–0.017)	**< 0.001**
Language	English-speaking: no vs. yes	24 (− 5 to 52)	0.100	0.067 (0.009–0.126)	**0.024**	− 0.073 (− 0.082 to − 0.064)	**< 0.001**	0.025 (0.023–0.028)	**< 0.001**
Health System	Universal Health Coverage Index (UHC)	11 (9–14)	**< 0.001**	− 0.003 (− 0.009 to 0.002)	0.194	0.003 (0.002–0.004)	**< 0.001**	− 0.001 (− 0.002 to − 0.001)	** < 0.001**
	General government expenditure on health (%)	2.026 (− 0.008 to 4.060)	0.051	− 0.006 (− 0.010 to − 0.001)	**0.008**	0.004 (0.003–0.004)	**< 0.001**	− 0.001 (− 0.001 to − 0.001)	**< 0.001**
	Number of pharmacists per capita	− 10 (− 13 to − 6)	**< 0.001**	− 0.026 (− 0.033 to − 0.019)	**< 0.001**	− 0.000 (− 0.001 to 0.001)	0.875	6.611e − 04 (3.536e − 04–9.685e − 04)	** < 0.001**
Gender equity	Gender Inequality Index (*per n*)	− 437 (− 543 to − 330)	**< 0.001**	0.430 (0.210–0.650)	**< 0.001**	− 0.095 (− 0.129 to − 0.061)	** < 0.001**	0.042 (0.033–0.051)	**< 0.001**
	Maternal mortality rate (*per n*)	− 0.951 (− 1.234 to − 0.668)	** < 0.001**	− 0.001 (− 0.001 to − 0.000)	**0.006**	− 3.368e − 04 (− 4.267e − 04 to − 2.469e − 04)	**< 0.001**	1.612e − 05 (− 7.467e − 06 to 3.971e − 05)	0.180
	Adolescent birth rate (*per n*)	− 5 (− 7 to − 4)	**< 0.001**	0.000 (− 0.002 to 0.003)	0.834	− 4.190e − 06 (− 4.297e − 04 to 4.213e − 04)	0.985	7.471e − 04 (6.360e − 04–8.582e − 04)	**< 0.001**
	Employment gap (male − female) (*per* %)	− 4 (− 5 to − 3)	**< 0.001**	0.006 (0.004–0.008)	**< 0.001**	− 0.002 (− 0.002 to − 0.002)	**< 0.001**	8.829e − 04 (7.938e − 04–9.720e − 04)	**< 0.001**
	Education gap (male − female) (*per %*)	− 2 (− 4 to 1)	0.247	0.018 (0.012–0.024)	**< 0.001**	− 0.007 (− 0.008 to − 0.006)	**< 0.001**	0.001 (0.001–0.001)	**< 0.001**
	Female share of parliamentary seats (*per %*)	6 (5–8)	**< 0.001**	0.002 (− 0.001 to 0.005)	0.262	0.003 (0.002–0.003)	**< 0.001**	− 2.444e − 04 (− 3.647e − 04 to − 1.240e − 04)	**< 0.001**
Human development	Human Development Index (*per n*)	438 (267–609)	**< 0.001**	− 0.631 (− 0.982 to − 0.280)	**< 0.001**	0.391 (0.337–0.445)	**< 0.001**	− 0.065 (− 0.080 to − 0.051)	**< 0.001**
	Gross national income per capita (*per* USD)	0.001 (− 0.000 to 0.001)	0.070	− 3.823e − 06 (− 5.148e − 06 to − 2.498e − 06)	** < 0.001**	1.455e − 06 (1.251e − 06–1.659e − 06)	**< 0.001**	− 3.224e − 07 (− 3.760e − 07 to − 2.689e − 07)	**< 0.001**
	Expected years of schooling (*per year*)	33 (26–41)	**< 0.001**	0.023 (0.008–0.039)	**0.003**	0.013 (0.011–0.015)	**< 0.001**	− 0.001 (− 0.002 to − 0.001)	**< 0.001**
	Mean years of schooling (*per year*)	− 3 (− 10 to 3)	0.276	− 0.028 (− 0.041 to − 0.015)	**< 0.001**	0.017 (0.015–0.019)	**< 0.001**	− 0.002 (− 0.003 to − 0.002)	**< 0.001**
	Life expectancy at birth (*per year*)	19 (15–23)	**< 0.001**	− 0.002 (− 0.010 to 0.006)	0.665	0.004 (0.003–0.005)	**< 0.001**	− 6.832e − 04 (− 9.990e − 04 to − 3.675e − 04)	**< 0.001**
Budget. Policies	GDP spent on research (*per* %)	5 (− 6 to 17)	0.358	− 0.059 (− 0.083 to − 0.035)	**< 0.001**	0.012 (0.008–0.015)	**< 0.001**	− 0.013 (− 0.013 to − 0.012)	**< 0.001**
	GDP spent on education (*per %*)	15.640 (0.682–30.599)	**0.040**	− 0.009 (− 0.039 to 0.022)	0.588	0.036 (0.031–0.040)	**< 0.001**	− 0.010 (− 0.011 to − 0.009)	**< 0.001**
	GDP spent on health (*per %*)	0.182 (− 3.147 to 3.511)	0.915	− 0.026 (− 0.033 to − 0.019)	**< 0.001**	0.008 (0.007–0.009)	**< 0.001**	− 0.002 (− 0.003 to − 0.002)	**< 0.001**
Disease burden	DALYs attributed to COPD	0.018 (− 0.014 to 0.049)	0.269	− 3.228e − 05 (− 9.635e − 05 to 3.178e − 05)	0.323	7.567e − 07 (− 9.148e − 06 to 1.066e − 05)	0.881	− 3.131e − 05 (− 3.387e − 05 to − 2.875e − 05)	**< 0.001**
	DALYs attributed to diabetes mellitus	− 0.170 (− 0.213 to − 0.126)	**< 0.001**	− 3.830e − 04 (− 4.726e − 04 to − 2.935e − 04)	** < 0.001**	5.004e − 07 (− 1.336e − 05 to 1.436e − 05)	0.944	− 1.172e − 05 (− 1.535e − 05 to − 8.088e − 06)	** < 0.001**
	DALYs attributed to HIV/AIDS	− 0.032 (− 0.056 to − 0.007)	**0.011**	− 9.597e − 06 (− 5.937e − 05 to 4.018e − 05)	0.705	− 2.355e − 06 (− 1.005e − 05 to 5.340e − 06)	0.549	− 3.329e − 06 (− 5.347e − 06 to − 1.312e − 06)	**0.001**
	DALYs attributed to ischemic heart disease	− 0.079 (− 0.097 to − 0.061)	**< 0.001**	− 6.808e − 05 (− 1.047e − 04 to − 3.146e − 05)	**< 0.001**	− 2.578e − 06 (− 8.242e − 06 to 3.085e − 06)	0.372	9.602e − 06 (8.122e − 06–1.108e − 05)	**< 0.001**
	DALYs attributed to neoplasms	0.045 (0.035–0.055)	**< 0.001**	− 7.550e − 05 (− 9.601e − 05 to − 5.499e − 05)	**< 0.001**	6.261e − 06 (3.088e − 06–9.435e − 06)	**< 0.001**	− 1.359e − 06 (− 2.192e − 06 to − 5.270e − 07)	**0.001**
	DALYs attributed to stroke	0.020 (0.003–0.038)	**0.025**	− 5.467e − 05 (− 9.134e − 05 to − 1.800e − 05)	**0.003**	− 4.388e − 05 (− 4.952e − 05 to − 3.824e − 05)	**< 0.001**	− 1.532e − 06 (− 3.019e − 06 to − 4.538e − 08)	**0.043**

Each national-level determinant is adjusted (Adj. *β*) for gender and academic age

Values in bold refer to statistical significance (*p*. <0.05)

In the second strategy, male gender (adj. *β* = 1283; 141) and academic age (adj. *β* = 142; 5) emerged as significant positive predictors, while gender inequality retained a negative effect (adj. *β* = –9689; − 75). These associations were similarly reflected when the modified *H*-index and composite score were analyzed as dependent variables (Table 10).

**Table 10:** Individual-level analysis: multivariable regression models of scholarly outputs of pharmacologic scholars in the Stanford–Elsevier Lists (SEL) of top scientists worldwide (2017–2023)

Career-long SEL	Citation count		Modified H-index		Composite score (C)		% Self-citations		
	**R**^**2**^** = 0.041**		**R**^**2**^** = 0.114**		**R**^**2**^** = 0.117**		**R**^**2**^** = 0.102**		
	**Adj. β (95% CI)**	**p**	**Adj. β (95% CI)**	**p**	**Adj. β (95% CI)**	**p**	**Adj. β (95% CI)**		**p**
Gender (male vs. female)	1283 (993–1573)	** < 0.001**	1.104 (0.850–1.358)	** < 0.001**	0.060 (0.049–0.070)	** < 0.001**	− 0.001 (− 0.003 to 0.002)		0.607
Academic age (per year)	142 (132–153)	** < 0.001**	0.255 (0.246–0.264)	** < 0.001**	0.008 (0.008–0.009)	** < 0.001**	− 0.000 (− 0.001 to − 0.000)		** < 0.001**
World Bank (low vs. high income)	2736 (− 5091 to 10,563)	0.493	2.835 (− 4.015 to 9.685)	0.417	0.154 (− 0.127 to 0.434)	0.283	− 0.204 (− 0.262 to − 0.147)		** < 0.001**
World Bank (lower-middle vs. high income)	164 (− 1934 to 2261)	0.879	1.584 (− 0.252 to 3.420)	0.091	0.014 (− 0.061 to 0.089)	0.712	− 0.109 (− 0.125 to − 0.094)		** < 0.001**
World Bank (upper-middle vs. high income)	1477 (404–2551)	**0.007**	4.076 (3.136–5.015)	** < 0.001**	0.010 (− 0.028 to 0.049)	0.599	− 0.046 (− 0.053 to − 0.038)		** < 0.001**
WHO region (AFRO vs. AMRO)	− 3424 (− 5995 to − 853)	**0.009**	− 2.072 (− 4.323 to 0.178)	0.071	− 0.004 (− 0.097 to 0.09)	0.925	− 0.098 (− 0.117 to − 0.079)		** < 0.001**
WHO region (EMRO vs. AMRO)	− 991 (− 2063 to 81)	0.070	1.006 (0.068–1.945)	**0.036**	− 0.034 (− 0.072 to 0.01)	0.087	0.012 (0.004–0.020)		**0.003**
WHO region (EURO vs. AMRO)	− 685 (− 1114 to − 255)	**0.002**	− 0.260 (− 0.636 to 0.116)	0.175	0.003 (− 0.012 to 0.019)	0.682	0.012 (0.009–0.015)		** < 0.001**
WHO region (SEARO vs. AMRO)	− 1198 (− 2700 to 303)	0.118	2.290 (0.976–3.604)	** < 0.001**	− 0.031 (− 0.085 to 0.02)	0.261	− 0.035 (− 0.046 to − 0.024)		** < 0.001**
WHO region (WPRO vs. AMRO)	− 1391 (− 1887 to − 894)	** < 0.001**	0.107 (− 0.327 to 0.542)	0.628	− 0.070 (− 0.088 to − 0.05)	** < 0.001**	0.014 (0.011–0.018)		** < 0.001**
English-speaking (yes vs. no)	199 (− 155 to 554)	0.270	1.027 (0.717–1.337)	** < 0.001**	0.076 (0.063–0.089)	** < 0.001**	− 0.032 (− 0.035 to − 0.029)		** < 0.001**
Gender Inequality Index (per n)	− 9689 (− 13,045 to − 6332)	** < 0.001**	− 7.564 (− 10.5 to − 4.6)	** < 0.001**	− 0.189 (− 0.309 to − 0.07)	**0.002**	0.120 (0.095–0.144)		** < 0.001**
Human Development Index (per n)	− 4921 (− 12,465 to 2623)	0.201	14.453 (7.851–21.056)	** < 0.001**	0.203 (− 0.067 to 0.473)	0.141	− 0.332 (− 0.387 to − 0.277)		** < 0.001**
Universal Health Coverage Index (UHC)	12 (− 87 to 110)	0.819	− 0.151 (− 0.238 to − 0.065)	** < 0.001**	0.000 (− 0.003 to 0.004)	0.924	− 0.001 (− 0.002 to − 0.001)		** < 0.001**
**Single-year SEL**	**Citation count**		**Modified H-index**		**Composite score (C)**		**% Self-citations**		
	**R**^**2**^** = 0.020**		**R**^**2**^** = 0.041**		**R**^**2**^** = 0.088**		**R**^**2**^** = 0.110**		
	**Adj. β (95% CI)**	**p**	**Adj. β (95% CI)**	**p**	**Adj. β (95% CI)**	**p**		**Adj. β (95% CI)**	**p**
Gender (male vs. female)	141 (109–173)	** < 0.001**	0.241 (0.176–0.307)	** < 0.001**	0.055 (0.045–0.065)	** < 0.001**		0.008 (0.005–0.011)	** < 0.001**
Academic age (per year)	5 (4–6)	** < 0.001**	0.031 (0.029–0.033)	** < 0.001**	0.005 (0.005–0.005)	** < 0.001**		− 0.001 (− 0.002 to − 0.001)	** < 0.001**
World Bank (low vs. high income)	273 (− 287 to 833)	0.339	− 0.540 (− 1.692 to 0.612)	0.358	0.002 (− 0.175 to 0.179)	0.983		− 0.108 (− 0.154 to − 0.063)	** < 0.001**
World Bank (lower-middle vs. high income)	− 287 (− 451 to − 124)	** < 0.001**	0.215 (− 0.121 to 0.552)	0.209	− 0.012 (− 0.064 to 0.04)	0.645		− 0.018 (− 0.032 to − 0.005)	**0.007**
World Bank (upper-middle vs. high income)	28 (− 89 to 145)	0.638	0.545 (0.305–0.784)	** < 0.001**	0.011 (− 0.026 to 0.047)	0.571		− 0.013 (− 0.023 to − 0.004)	**0.006**
WHO region (AFRO vs. AMRO)	− 320 (− 544 to − 96)	**0.005**	− 0.133 (− 0.594 to 0.327)	0.571	− 0.041 (− 0.112 to 0.03)	0.257		− 0.049 (− 0.067 to − 0.031)	** < 0.001**
WHO region (EMRO vs. AMRO)	− 8 (− 99 to 83)	0.863	0.436 (0.248–0.623)	** < 0.001**	0.005 (− 0.024 to 0.034)	0.737		0.011 (0.004–0.019)	**0.003**
WHO region (EURO vs. AMRO)	190 (132–248)	** < 0.001**	0.448 (0.330–0.567)	** < 0.001**	0.067 (0.049–0.086)	** < 0.001**		0.016 (0.011–0.020)	** < 0.001**
WHO region (SEARO vs. AMRO)	− 28 (− 141 to 85)	0.631	0.702 (0.470–0.935)	** < 0.001**	0.004 (− 0.032 to 0.040)	0.830		− 0.033 (–0.043 to − 0.024)	** < 0.001**
WHO region (WPRO vs. AMRO)	71 (5–137)	**0.035**	0.493 (0.357–0.629)	** < 0.001**	0.021 (− 0.000 to 0.041)	0.054		− 0.005 (–0.011 to − 0.000)	**0.047**
English-speaking (yes vs. no)	86 (38–134)	** < 0.001**	0.213 (0.115–0.312)	** < 0.001**	0.073 (0.058–0.089)	** < 0.001**		− 0.024 (–0.028 to − 0.020)	** < 0.001**
Gender Inequality Index (per n)	− 75 (− 447 to 296)	0.691	1.181 (0.417–1.946)	**0.002**	0.238 (0.121–0.356)	** < 0.001**		0.078 (0.048 to 0.109)	** < 0.001**
Human Development Index (per n)	− 1293 (− 2121 to − 465)	**0.002**	3.220 (1.517–4.923)	** < 0.001**	0.526 (0.264–0.787)	** < 0.001**		0.019 (–0.048 to 0.087)	0.575
Universal Health Coverage Index (UHC)	5 (− 3 to 13)	0.238	− 0.007 (− 0.024 to 0.009)	0.389	− 0.002 (− 0.005 to 0.00)	0.059		− 0.002 (–0.002 to − 0.001)	** < 0.001**

Values in bold refer to statistical significance (*p*. <0.05)

## Discussion

### Key findings

The present study analyzed 56,358 EPPS records, with 55.1% included in the *career-long* SEL and 44.9% in the *single-year* SEL. Data completeness was high (country 98.3%, institution 98.5%, gender 91.3%, academic age 96.3%), and EPPS counts rose steadily between 2017 and 2023. High-income countries predominated (*career-long* 93.0%; *single-year* 77.5%), though upper- and lower-middle-income countries expanded their shares in recent years. The USA contributed the largest proportion (34.9% *career-long*; 25.4% *single-year*), while English-speaking countries collectively accounted for 53.7% and 40.5%. EPPS density per 100,000 population was 0.456 (*career-long*) and 0.365 (*single-year*), with stark inequalities between HICs (2.043) and LICs (0.004); Iceland, New Zealand, and Denmark exhibited the highest densities.

Institutional concentration was also marked, with the top 20 institutions hosting 12.7% of *career-long* and 10.3% of *single-year* EPPS. Female representation remained limited at 16.8% (*career-long*) and 25.0% (*single-year*), although upper-middle-income countries reported higher shares (24.4% and 30.1%). Males demonstrated higher median citations, modified *H*-indices, and older academic age in both datasets (all *p* < 0.001). Academic age correlated positively with core bibliometric outcomes (e.g., modified *H*-index *ρ* = 0.330 *career-long*; composite score *ρ* = 0.243 *single-year*), with stronger authorship-role correlations in the *single-year* SEL. Over time (2017–2023), HIC dominance declined, EMRO contributions rose, female representation increased, and regression analyses confirmed academic age and male gender as independent positive predictors of excellence.

### Global disparities of pharmacology and pharmacy research excellence

Our analysis showed that EPPS were predominantly concentrated in high-income countries, with the USA contributing the largest share across both datasets. EPPS counts were strongly correlated with national economic indicators, suggesting that *research excellence* in this field remains closely tied to macroeconomic capacity. Comparable geographical concentration has been consistently reported in productivity-oriented bibliometric studies (Sweileh et al. 2018, 2019; AL-Aqeel et al. 2020; Mendes et al. 2019; Saeed et al. 2024). A bibliometric analysis of Scopus-indexed publications on *pharmacy education* (2000–2016) showed that the USA accounted for more than half of global output (53.6%), followed by the UK (6.2%), India (3.9%), Australia (3.7%), and Canada (3.7%) (Sweileh et al. 2018). Consistently, a Scopus-based analysis of *medication adherence* research (1900–2017) reported that the USA dominated global productivity (43.1%), followed by the UK (8.6%), Canada (4.7%), and Germany (3.9%) (Sweileh et al. 2019). Similarly, an analysis of *pharmaceutical care* literature in the Web of Science (WoS) (2002–2021) found that ten high-income countries generated 81.9% of global output, with the USA leading (33.6%), followed by Australia (8.1%) and the UK (7.2%) (Wang et al. 2022). In *pharmacy practice* literature, a bibliometric analysis of Mendes’ map of pharmacy journals (2009–2018) also demonstrated the dominance of high-income countries, with the USA contributing 45.7%, followed by France (7.2%), the UK (6.8%), Canada (6.8%), and Australia (5.8%) (AL-Aqeel et al. 2020; Mendes et al. 2019). More recently, a Scopus-based analysis of *telepharmacy* literature (1981–2023) showed that the USA accounted for half of global output (51.5%), ahead of Australia (4.8%), Canada (4.5%), and Spain (4.5%) (Saeed et al. 2024). The only notable exception to US dominance is *pharmacovigilance* literature, where a WoS-based analysis (1974–2021) showed France leading (24.5%), followed by the USA (17.2%), the UK (13.4%), and the Netherlands (10.7%) (Wang et al. 2021).

Evidence from a cross-country analysis (1996–2012) demonstrated that scientific publication activity and GDP growth are mutually reinforcing, with wealthier nations better positioned to sustain both high research productivity and subsequent economic returns (Dębski et al. 2018). A competitiveness-based analysis of 27 pharmaceutical-exporting countries (2000–2014) further showed that pharmaceutical exports and revealed comparative advantage were significant positive predictors of both GDP and GDP per capita, alongside human capital, capital stock, and productivity (Muratoğlu 2025). These findings highlight how national economic strength and industrial capacity in pharmaceuticals reinforce disparities in research productivity across countries (Muratoğlu 2025).

### English language hegemony in pharmacologic scholarship

EPPS affiliations were heavily concentrated in English-speaking settings, suggesting that linguistic context contributes to observed disparities in *research excellence*. The dominance of English in medical and pharmaceutical science is a relatively recent outcome of historical and geopolitical shifts, replacing earlier eras when Greek, Latin, French, and German played central roles (Henrik 2004; Gordin 2015). Today, more than 90% of natural science publications appear in English, even though it is the native language of only a small minority of the world’s population, creating systemic disadvantages for non-Anglophone scholars (Bahji et al. 2022). The academic imperative to “publish or perish” compels scholars to target high-impact journals, which are overwhelmingly English, making English less a choice than a prerequisite for career success (Di Bitetti and Ferreras 2016; Andersen and Hellman 2021). In pharmacology and pharmacy, a Scopus-based bibliometric analysis (2017–2021) of 38,624 publications found that 95.4% were published in English, with the USA leading global output and China’s rapid growth further reinforcing the centrality of English for international visibility (Kannan et al. 2022).

Empirical studies highlight how these linguistic dynamics exacerbate inequalities. Roha (2011) surveyed corresponding authors in international pharmacology journals and found that only 30.4% of publications originated from less-developed countries (LDCs), with most respondents citing editorial bias and poor writing skills as major obstacles (Rohra 2011). Such barriers reinforce the dominance of English, where visibility and impact often depend on costly editorial and publication services (Chen 2024). As a result, researchers from LDCs are disproportionately confined to lower-impact or non-indexed journals, while non-English medical journals continue to lag behind their English-language counterparts in impact factor trends (Vinther and Rosenberg 2012). Together, these factors give scholars from high-income and Anglophone countries a significant advantage in shaping the global *research excellence* landscape examined in the present study.

English language dominance, however, is a double-edged phenomenon. On the one hand, adopting a universal lingua franca facilitates science communication and accelerates innovation, particularly in high-demand fields such as pharmacology that are central to drug discovery. On the other hand, it creates a cascade of inequalities. Beyond *research excellence* metrics, its effects are evident in various domains: firstly, non-native English-speaking researchers face economic and professional burdens linked to translation, editing, and linguistic bias (Scientific publishing has a language problem 2023); secondly, citation practices in Anglophone regions systematically neglect non-English literature, narrowing the global evidence base (PhannD and Donovan PhannD 2009); thirdly, systematic reviews frequently exclude non-English studies, reducing inclusiveness and precision in evidence synthesis (Jüni et al. 2002; Jackson et al. 2019)]; and fourthly, grant funding success is increasingly tied to rhetorical mastery of English, amplifying inequities in access to resources and visibility (Millar et al. 2022; Connor and Mauranen 1999).

### Institutional elitism in pharmacologic scholarship

The present analysis revealed that EPPS are disproportionately concentrated within a small number of world-leading institutions, underscoring how *research excellence* is unevenly distributed not only across countries but also within them. At the national level, a Scopus-based bibliometric analysis of *pharmaceutical care* literature from Saudi Arabia (2002–2021) showed a sharp imbalance, with nearly half of the national output produced by just two universities: King Saud University (40.4%) and King Abdulaziz University (9.5%) (Alotaibi et al. 2022). At the regional level, a Scopus-based analysis of *pharmaceutical care* literature from the Arab world (1990–2020) found that the top five institutions accounted for 45.9% of output, led by Qatar University (13.6%), King Saud University (11.8%), and the University of Jordan (8.8%) (Sweileh 2021).

Globally, the *drug repositioning* literature indexed in WoS (1990–2020) was dominated by 20 institutions that together accounted for 19.4% of publications, led by the Chinese Academy of Sciences (1.8%), Case Western Reserve University (1.3%), and the National Institutes of Health (1.2%) (Sun et al. 2022). Similarly, the *pharmacovigilance* literature indexed in WoS (1974–2021) was concentrated in just five institutions, which collectively accounted for 14.4% of global output (Wang et al. 2021). The *pharmacy education* literature indexed in Scopus (2000–2016) was likewise dominated by five institutions producing 12.2% of global output (Sweileh et al. 2018), while another WoS-based analysis of *pharmaceutical care* literature (2002–2021) found that just three institutions accounted for 7.1% of global output (Wang et al. 2022).

Taken together, these findings highlight a persistent pattern of institutional elitism across national, regional, and global scales, whereby a small subset of universities and research centers capture a disproportionate share of pharmacological scholarship. This pattern aligns with the highly skewed, power-law distributions of scientific productivity described in classic bibliometric work, including Lotka’s law (Lotka 1926). Nonetheless, our analysis showed emerging diversification over time: the share of EPPS hosted by the top 20 institutions declined across successive SEL updates, and a greater number of countries and institutions appeared in the *single-year* lists compared with the *career-long* lists. These trends indicate that, although concentration remains a defining feature of the field, excellence is becoming more diffuse, with growing representation from middle-income countries and newer institutions.

### Gender disparities in pharmacologic research performance

The present analysis demonstrated that women were notably underrepresented among EPPS, with lower bibliometric outcomes and younger median academic ages than their male counterparts. This gender disparity is also reflected in pharmacologic research production, as a gender-specific analysis of *Naunyn–Schmiedeberg’s Archives of Pharmacology* (2000–2020) found that female authorship stagnated at around 30%, with women holding only about 15% of senior authorship positions despite political initiatives to increase participation (Zehetbauer et al. 2022). Likewise, another analysis of Canadian pharmacoepidemiology publications (2012–2017) found that women accounted for 36% of authorships compared with 29% in the broader citing literature, yet they remained underrepresented in senior positions, with only 14% serving as corresponding authors (Sketris et al. 2022). In Germany, an analysis of 1327 articles in the non-peer-reviewed magazine *Biospektrum* (1999–2021) found pharmacology underrepresented and women persistently underrepresented—particularly in senior roles—with female first authorships declining during the COVID-19 period and publications concentrated in just three institutions (Zöllner and Seifert 2024). In a matched case–control analysis of 46,235 invited commentaries from medical journals (2013–2017) using Scopus data, women had 21% lower odds of corresponding authorship than comparable men, even after adjustment for field, years active, publication count, and citation impact, with the gap widening at higher academic ages (Thomas et al. 2019). By contrast, a bibliometric analysis of leading pharmacy journals indexed in WoS (2007–2017) reported more gender-balanced authorship, with women representing 51.1% of first authors in research articles, 50.6% in review articles, 37.1% in editorials, and 42.1% in letters (Nguyen et al. 2021). The persistence of female underrepresentation reinforces homophily in pharmacologic research, narrowing the spectrum of questions addressed and risking the neglect of domains particularly relevant to women, thereby constraining the inclusiveness and robustness of scientific progress (Kozlowski et al. 2022; Heidari et al. 2016).

### Impact of academic age on pharmacologic scholarship

Academic age, according to the present analysis, was the only consistent positive predictor of EPPS counts and their bibliometric outcomes, indicating that research impact in pharmacology and pharmacy accumulates with sustained career duration. In an Italian study of 30,677 university researchers (2004–2008), research productivity followed a pyramidal structure, with full professors achieving the highest outputs, associates intermediate, and assistants the lowest. This hierarchy highlights how academic rank strongly mirrors cumulative advantages in research performance and visibility (Abramo et al. 2011). Another Italian analysis of 11,989 full professors (2006–2010) showed that research productivity declined steadily with age but increased with seniority in rank, with those promoted earlier sustaining higher performance over time and illustrating the cumulative advantage shaping long-term research outcomes (Abramo et al. 2016).

Zaorsky et al. (2020) conducted a systematic review and meta-analysis of 21 studies including 14,567 academic physicians that revealed a stepwise increase in research productivity by rank, with mean *H*-indices of 5.2 for assistant professors, 11.2 for associate professors, 20.8 for full professors, and 22.1 for department chairs, paralleled by mean *M*-indices of 0.53, 0.72, 0.99, and 1.16, respectively (Zaorsky et al. 2020). Performance curves of researchers in medical disciplines, including pharmacology, exhibit three distinct phases—initiation (25–35 years), maturation (35–50 years), and stabilization or decline (50 + years)—with professors consistently outperforming non-academic physicians and younger cohorts demonstrating higher productivity at comparable ages (Duclos et al. 2017).

### Strengths

Firstly, this study makes an innovative contribution by shifting the focus from mere research productivity to empirically defined excellence, operationalized through the SELs in both *career-long* and *single-year* dimensions. Secondly, it applies a socio-ecological framework that integrates national, institutional, and individual-level determinants, providing a nuanced understanding of multilevel influences on pharmacology and pharmacy *research excellence*. Thirdly, the analysis draws on a large dataset of 56,358 records with high completeness across key variables, ensuring a reliable assessment of global patterns and disparities. Fourthly, transparent and robust analytical strategies were employed, including non-parametric tests, multivariable regressions, and trend analyses, conducted in line with bibliometric reporting standards. Finally, the study adopts rigorous bibliometric operationalization, incorporating citation counts excluding self-citations, co-authorship-adjusted indices, composite scores, and harmonized institutional data to enhance accuracy and reproducibility.

### Limitations

The present analysis has some limitations; firstly, excellence was operationalized through citation-based indicators, which privilege indexed visibility and may not fully reflect societal or clinical impact despite efforts to mitigate biases. Secondly, gender assignment relied on probabilistic name-based inference with binary categories, which may lead to misclassification and exclude non-binary identities. Thirdly, academic age, measured as years since first indexed publication, is an imperfect proxy as it cannot account for career interruptions, part-time appointments, or alternative scholarly outputs. Fourthly, disciplinary classifications and reliance on Scopus coverage risk misclassifying researchers at field boundaries and under-representing contributions from LMICs and non-English literature. Fifthly, partial circularity may occur because of the inclusion of scientometric indicators in global university rankings, although these rankings also incorporate broader dimensions such as teaching environment, institutional resources, and internationalization, meaning that any overlap may inflate associations but does not invalidate the institutional patterns observed. Finally, an inherent limitation of the SEL methodology is the duplicate author profiles in the Scopus database, which fragment publication records and citations, reducing individual impact metrics; therefore, our analysis used author records rather than individual persons as the unit of analysis (Vasan et al. 2023).

### Implications

Firstly, future research should investigate EPPS career curves with attention to generational effects, explore causal pathways between national capacity and excellence, and assess editorial and peer-review practices for bias. Secondly, the concentration of excellence in high-income settings highlights the need to rebalance investment and incentives by strengthening capacity in underrepresented countries and institutions. Thirdly, the overwhelming dominance of English underscores the urgency of mitigating language-driven visibility gaps through multilingual abstracts, translation support, and equitable publication policies. Fourthly, the persistent underrepresentation of women calls for gender-equitable assessment frameworks, inclusive mentorship, and leadership opportunities to foster balanced scientific advancement. Finally, higher education institutions should encourage their affiliated academics to maintain accurate and up-to-date research profiles, including Scopus author records, to minimize duplication and ensure the integrity of bibliometric data (University of Bristol 2025; University of Leeds 2025).

## Conclusion

This study highlights how pharmacology and pharmacy *research excellence*, rather than simple productivity, is distributed and determined across global, institutional, and individual levels. It shows that excellence remains concentrated in high-income countries and a limited number of universities, while English-language dominance amplifies visibility disparities. At the individual level, gender imbalances persist, and academic age stands out as the strongest predictor of research impact. Together, these findings suggest that excellence in pharmacology is shaped by systemic disparities in economic capacity, institutional prestige, and linguistic visibility, with implications for funding priorities, collaboration strategies, and evaluation systems. Despite methodological limitations, this analysis establishes a foundation for future research into cohort-sensitive career trajectories and inclusive excellence frameworks.

## Supplementary Information

Below is the link to the electronic supplementary material.

Supplementary file1 (DOCX 176 KB)

## Data Availability

Publicly available datasets were analysed in this study. This data can be found at: —SEL Version 1 (July 2019); DOI: [10.17632/btchxktzyw.1](https:/elsevier.digitalcommonsdata.com/datasets/btchxktzyw/1)—SEL Version 2 (October 2020); DOI: [10.17632/btchxktzyw.2](https:/elsevier.digitalcommonsdata.com/datasets/btchxktzyw/2)—SEL Version 3 (October 2021); DOI: [10.17632/btchxktzyw.3](https:/elsevier.digitalcommonsdata.com/datasets/btchxktzyw/3)—SEL Version 4 (October 2022); DOI: [10.17632/btchxktzyw.4](https:/elsevier.digitalcommonsdata.com/datasets/btchxktzyw/4)—SEL Version 5 (November 2022); DOI: [10.17632/btchxktzyw.5](https:/elsevier.digitalcommonsdata.com/datasets/btchxktzyw/5)—SEL Version 6 (October 2023); DOI: [10.17632/btchxktzyw.6](https:/elsevier.digitalcommonsdata.com/datasets/btchxktzyw/6)—SEL Version 7 (September 2024); DOI: [10.17632/btchxktzyw.7](https:/elsevier.digitalcommonsdata.com/datasets/btchxktzyw/7).
