# Supplementary material for: National-, institutional-, and individual-level determinants of pharmacology and pharmacy research excellence: an analysis of stanford–elsevier lists of the top 2% scholars (2017–2023)
Source: Naunyn Schmiedebergs Arch Pharmacol. 2026 Jan 30;399(7):10129–61. doi: 10.1007/s00210-025-04949-4 (PMC13153008; doi:10.1007/s00210-025-04949-4)
Supplement: Supplementary file 1 — Supplementary file1 (DOCX 176 KB) [file 210_2025_4949_MOESM1_ESM.docx]

**Table S1.** National-level Analysis: Distribution of Pharmacologic Scholars in the *Career-Long* Stanford-Elsevier Lists (SEL) of Top 2% Scientists Worldwide (2017–2023)

| **#** | **Country** | **SEL 2017** | **SEL 2018** | **SEL 2019** | **SEL 2020** | **SEL 2021** | **SEL 2022** | **SEL 2023** | **Total ▼** | **Σ%** |
| --- | --- | --- | --- | --- | --- | --- | --- | --- | --- | --- |
| **1** | United States of America | 1009 (39.20%) | 1118 (40.22%) | 1428 (36.53%) | 1721 (34.11%) | 1759 (33.92%) | 1778 (33.10%) | 1824 (32.55%) | 10637 (34.91%) | 34.91% |
| **2** | United Kingdom | 353 (13.71%) | 375 (13.49%) | 457 (11.69%) | 566 (11.22%) | 562 (10.84%) | 573 (10.67%) | 593 (10.58%) | 3479 (11.42%) | 46.33% |
| **3** | Japan | 129 (5.01%) | 147 (5.29%) | 247 (6.32%) | 304 (6.03%) | 306 (5.90%) | 332 (6.18%) | 327 (5.84%) | 1792 (5.88%) | 52.21% |
| **4** | Germany | 155 (6.02%) | 168 (6.04%) | 237 (6.06%) | 298 (5.91%) | 301 (5.80%) | 304 (5.66%) | 320 (5.71%) | 1783 (5.85%) | 58.06% |
| **5** | Italy | 107 (4.16%) | 127 (4.57%) | 159 (4.07%) | 222 (4.40%) | 220 (4.24%) | 226 (4.21%) | 235 (4.19%) | 1296 (4.25%) | 62.31% |
| **6** | Canada | 122 (4.74%) | 107 (3.85%) | 159 (4.07%) | 184 (3.65%) | 193 (3.72%) | 193 (3.59%) | 200 (3.57%) | 1158 (3.80%) | 66.11% |
| **7** | France | 101 (3.92%) | 117 (4.21%) | 131 (3.35%) | 177 (3.51%) | 184 (3.55%) | 180 (3.35%) | 186 (3.32%) | 1076 (3.53%) | 69.65% |
| **8** | Australia | 80 (3.11%) | 75 (2.70%) | 107 (2.74%) | 141 (2.79%) | 136 (2.62%) | 141 (2.62%) | 147 (2.62%) | 827 (2.71%) | 72.36% |
| **9** | Netherlands | 67 (2.60%) | 67 (2.41%) | 98 (2.51%) | 126 (2.50%) | 132 (2.55%) | 135 (2.51%) | 141 (2.52%) | 766 (2.51%) | 74.87% |
| **10** | Sweden | 58 (2.25%) | 43 (1.55%) | 71 (1.82%) | 95 (1.88%) | 103 (1.99%) | 100 (1.86%) | 99 (1.77%) | 569 (1.87%) | 76.74% |
| **11** | China | 18 (0.70%) | 25 (0.90%) | 66 (1.69%) | 94 (1.86%) | 101 (1.95%) | 115 (2.14%) | 131 (2.34%) | 550 (1.81%) | 78.55% |
| **12** | India | 12 (0.47%) | 18 (0.65%) | 46 (1.18%) | 88 (1.74%) | 97 (1.87%) | 123 (2.29%) | 140 (2.50%) | 524 (1.72%) | 80.27% |
| **13** | South Korea | 18 (0.70%) | 18 (0.65%) | 54 (1.38%) | 95 (1.88%) | 100 (1.93%) | 110 (2.05%) | 121 (2.16%) | 516 (1.69%) | 81.96% |
| **14** | Switzerland | 49 (1.90%) | 42 (1.51%) | 68 (1.74%) | 82 (1.63%) | 84 (1.62%) | 88 (1.64%) | 89 (1.59%) | 502 (1.65%) | 83.61% |
| **15** | Belgium | 37 (1.44%) | 39 (1.40%) | 60 (1.53%) | 81 (1.61%) | 78 (1.50%) | 79 (1.47%) | 85 (1.52%) | 459 (1.51%) | 85.11% |
| **16** | Spain | 25 (0.97%) | 28 (1.01%) | 47 (1.20%) | 59 (1.17%) | 74 (1.43%) | 82 (1.53%) | 86 (1.53%) | 401 (1.32%) | 86.43% |
| **17** | Denmark | 22 (0.85%) | 23 (0.83%) | 41 (1.05%) | 61 (1.21%) | 63 (1.21%) | 66 (1.23%) | 69 (1.23%) | 345 (1.13%) | 87.56% |
| **18** | New Zealand | 17 (0.66%) | 25 (0.90%) | 40 (1.02%) | 57 (1.13%) | 61 (1.18%) | 62 (1.15%) | 63 (1.12%) | 325 (1.07%) | 88.63% |
| **19** | Poland | 12 (0.47%) | 20 (0.72%) | 36 (0.92%) | 59 (1.17%) | 57 (1.10%) | 63 (1.17%) | 66 (1.18%) | 313 (1.03%) | 89.66% |
| **20** | Finland | 25 (0.97%) | 27 (0.97%) | 38 (0.97%) | 49 (0.97%) | 50 (0.96%) | 49 (0.91%) | 54 (0.96%) | 292 (0.96%) | 90.61% |
| **21** | Taiwan | 18 (0.70%) | 17 (0.61%) | 22 (0.56%) | 43 (0.85%) | 43 (0.83%) | 44 (0.82%) | 47 (0.84%) | 234 (0.77%) | 91.38% |
| **22** | Austria | 20 (0.78%) | 19 (0.68%) | 31 (0.79%) | 41 (0.81%) | 39 (0.75%) | 39 (0.73%) | 41 (0.73%) | 230 (0.75%) | 92.14% |
| **23** | Iran | 6 (0.23%) | 3 (0.11%) | 15 (0.38%) | 42 (0.83%) | 47 (0.91%) | 54 (1.01%) | 62 (1.11%) | 229 (0.75%) | 92.89% |
| **24** | Brazil | 11 (0.43%) | 11 (0.40%) | 24 (0.61%) | 41 (0.81%) | 42 (0.81%) | 43 (0.80%) | 49 (0.87%) | 221 (0.73%) | 93.61% |
| **25** | Israel | 24 (0.93%) | 26 (0.94%) | 26 (0.67%) | 30 (0.59%) | 32 (0.62%) | 35 (0.65%) | 36 (0.64%) | 209 (0.69%) | 94.30% |
| **26** | Hungary | 11 (0.43%) | 12 (0.43%) | 18 (0.46%) | 27 (0.54%) | 31 (0.60%) | 30 (0.56%) | 30 (0.54%) | 159 (0.52%) | 94.82% |
| **27** | Ireland | 13 (0.51%) | 13 (0.47%) | 18 (0.46%) | 21 (0.42%) | 20 (0.39%) | 19 (0.35%) | 19 (0.34%) | 123 (0.40%) | 95.22% |
| **28** | Egypt | 1 (0.04%) | 1 (0.04%) | 10 (0.26%) | 22 (0.44%) | 23 (0.44%) | 29 (0.54%) | 33 (0.59%) | 119 (0.39%) | 95.62% |
| **29** | Saudi Arabia | 0 (0.00%) | 0 (0.00%) | 10 (0.26%) | 18 (0.36%) | 20 (0.39%) | 27 (0.50%) | 31 (0.55%) | 106 (0.35%) | 95.96% |
| **30** | Portugal | 6 (0.23%) | 7 (0.25%) | 12 (0.31%) | 16 (0.32%) | 18 (0.35%) | 19 (0.35%) | 21 (0.37%) | 99 (0.32%) | 96.29% |
| **31** | Singapore | 3 (0.12%) | 5 (0.18%) | 14 (0.36%) | 17 (0.34%) | 17 (0.33%) | 21 (0.39%) | 20 (0.36%) | 97 (0.32%) | 96.61% |
| **32** | Norway | 6 (0.23%) | 5 (0.18%) | 10 (0.26%) | 18 (0.36%) | 17 (0.33%) | 18 (0.34%) | 20 (0.36%) | 94 (0.31%) | 96.91% |
| **33** | Hong Kong | 8 (0.31%) | 10 (0.36%) | 13 (0.33%) | 13 (0.26%) | 14 (0.27%) | 19 (0.35%) | 14 (0.25%) | 91 (0.30%) | 97.21% |
| **34** | Türkiye | 4 (0.16%) | 2 (0.07%) | 12 (0.31%) | 12 (0.24%) | 17 (0.33%) | 21 (0.39%) | 21 (0.37%) | 89 (0.29%) | 97.51% |
| **35** | Greece | 5 (0.19%) | 4 (0.14%) | 8 (0.20%) | 14 (0.28%) | 12 (0.23%) | 12 (0.22%) | 18 (0.32%) | 73 (0.24%) | 97.75% |
| **36** | Czechia | 2 (0.08%) | 7 (0.25%) | 10 (0.26%) | 12 (0.24%) | 11 (0.21%) | 12 (0.22%) | 13 (0.23%) | 67 (0.22%) | 97.97% |
| **37** | Thailand | 2 (0.08%) | 1 (0.04%) | 6 (0.15%) | 8 (0.16%) | 12 (0.23%) | 13 (0.24%) | 16 (0.29%) | 58 (0.19%) | 98.16% |
| **38** | Malaysia | 0 (0.00%) | 0 (0.00%) | 3 (0.08%) | 10 (0.20%) | 13 (0.25%) | 13 (0.24%) | 17 (0.30%) | 56 (0.18%) | 98.34% |
| **39** | South Africa | 3 (0.12%) | 3 (0.11%) | 5 (0.13%) | 7 (0.14%) | 6 (0.12%) | 8 (0.15%) | 13 (0.23%) | 45 (0.15%) | 98.49% |
| **40** | Pakistan | 2 (0.08%) | 3 (0.11%) | 4 (0.10%) | 9 (0.18%) | 9 (0.17%) | 10 (0.19%) | 7 (0.12%) | 44 (0.14%) | 98.63% |
| **41** | Mexico | 2 (0.08%) | 2 (0.07%) | 5 (0.13%) | 8 (0.16%) | 8 (0.15%) | 7 (0.13%) | 7 (0.12%) | 39 (0.13%) | 98.76% |
| **42** | Nigeria | 1 (0.04%) | 1 (0.04%) | 2 (0.05%) | 6 (0.12%) | 6 (0.12%) | 7 (0.13%) | 10 (0.18%) | 33 (0.11%) | 98.87% |
| **43** | Iceland | 2 (0.08%) | 2 (0.07%) | 2 (0.05%) | 2 (0.04%) | 5 (0.10%) | 6 (0.11%) | 7 (0.12%) | 26 (0.09%) | 98.95% |
| **44** | United Arab Emirates | 2 (0.08%) | 1 (0.04%) | 3 (0.08%) | 2 (0.04%) | 3 (0.06%) | 5 (0.09%) | 8 (0.14%) | 24 (0.08%) | 99.03% |
| **45** | Kuwait | 1 (0.04%) | 0 (0.00%) | 2 (0.05%) | 4 (0.08%) | 6 (0.12%) | 5 (0.09%) | 5 (0.09%) | 23 (0.08%) | 99.11% |
| **46** | Russia | 0 (0.00%) | 3 (0.11%) | 5 (0.13%) | 3 (0.06%) | 4 (0.08%) | 3 (0.06%) | 3 (0.05%) | 21 (0.07%) | 99.18% |
| **47** | Macao | 0 (0.00%) | 0 (0.00%) | 4 (0.10%) | 2 (0.04%) | 4 (0.08%) | 2 (0.04%) | 3 (0.05%) | 15 (0.05%) | 99.23% |
| **48** | State of Palestine | 0 (0.00%) | 0 (0.00%) | 2 (0.05%) | 2 (0.04%) | 3 (0.06%) | 3 (0.06%) | 5 (0.09%) | 15 (0.05%) | 99.27% |
| **49** | Qatar | 0 (0.00%) | 2 (0.07%) | 2 (0.05%) | 2 (0.04%) | 2 (0.04%) | 2 (0.04%) | 4 (0.07%) | 14 (0.05%) | 99.32% |
| **50** | Jordan | 0 (0.00%) | 0 (0.00%) | 1 (0.03%) | 2 (0.04%) | 2 (0.04%) | 4 (0.07%) | 4 (0.07%) | 13 (0.04%) | 99.36% |
| **51** | Bangladesh | 0 (0.00%) | 0 (0.00%) | 0 (0.00%) | 1 (0.02%) | 3 (0.06%) | 3 (0.06%) | 4 (0.07%) | 11 (0.04%) | 99.40% |
| **52** | Croatia | 0 (0.00%) | 0 (0.00%) | 1 (0.03%) | 2 (0.04%) | 2 (0.04%) | 3 (0.06%) | 3 (0.05%) | 11 (0.04%) | 99.44% |
| **53** | Romania | 0 (0.00%) | 0 (0.00%) | 2 (0.05%) | 1 (0.02%) | 2 (0.04%) | 3 (0.06%) | 3 (0.05%) | 11 (0.04%) | 99.47% |
| **54** | Serbia | 0 (0.00%) | 0 (0.00%) | 0 (0.00%) | 3 (0.06%) | 3 (0.06%) | 2 (0.04%) | 2 (0.04%) | 10 (0.03%) | 99.50% |
| **55** | Slovakia | 1 (0.04%) | 1 (0.04%) | 0 (0.00%) | 2 (0.04%) | 2 (0.04%) | 2 (0.04%) | 2 (0.04%) | 10 (0.03%) | 99.54% |
| **56** | Bahrain | 1 (0.04%) | 1 (0.04%) | 1 (0.03%) | 1 (0.02%) | 1 (0.02%) | 2 (0.04%) | 2 (0.04%) | 9 (0.03%) | 99.57% |
| **57** | Iraq | 0 (0.00%) | 0 (0.00%) | 0 (0.00%) | 0 (0.00%) | 2 (0.04%) | 3 (0.06%) | 4 (0.07%) | 9 (0.03%) | 99.60% |
| **58** | Trinidad and Tobago | 0 (0.00%) | 0 (0.00%) | 1 (0.03%) | 2 (0.04%) | 2 (0.04%) | 2 (0.04%) | 2 (0.04%) | 9 (0.03%) | 99.63% |
| **59** | Uruguay | 1 (0.04%) | 0 (0.00%) | 0 (0.00%) | 2 (0.04%) | 2 (0.04%) | 2 (0.04%) | 2 (0.04%) | 9 (0.03%) | 99.66% |
| **60** | Oman | 1 (0.04%) | 1 (0.04%) | 1 (0.03%) | 1 (0.02%) | 1 (0.02%) | 1 (0.02%) | 2 (0.04%) | 8 (0.03%) | 99.68% |
| **61** | Slovenia | 0 (0.00%) | 0 (0.00%) | 0 (0.00%) | 1 (0.02%) | 2 (0.04%) | 2 (0.04%) | 3 (0.05%) | 8 (0.03%) | 99.71% |
| **62** | Cyprus | 0 (0.00%) | 1 (0.04%) | 1 (0.03%) | 1 (0.02%) | 1 (0.02%) | 2 (0.04%) | 1 (0.02%) | 7 (0.02%) | 99.73% |
| **63** | Namibia | 0 (0.00%) | 1 (0.04%) | 1 (0.03%) | 1 (0.02%) | 1 (0.02%) | 2 (0.04%) | 1 (0.02%) | 7 (0.02%) | 99.75% |
| **64** | Argentina | 0 (0.00%) | 2 (0.07%) | 0 (0.00%) | 1 (0.02%) | 1 (0.02%) | 1 (0.02%) | 1 (0.02%) | 6 (0.02%) | 99.77% |
| **65** | Bulgaria | 0 (0.00%) | 1 (0.04%) | 1 (0.03%) | 1 (0.02%) | 1 (0.02%) | 1 (0.02%) | 1 (0.02%) | 6 (0.02%) | 99.79% |
| **66** | Venezuela | 0 (0.00%) | 0 (0.00%) | 1 (0.03%) | 2 (0.04%) | 1 (0.02%) | 1 (0.02%) | 1 (0.02%) | 6 (0.02%) | 99.81% |
| **67** | Cuba | 0 (0.00%) | 0 (0.00%) | 1 (0.03%) | 1 (0.02%) | 1 (0.02%) | 1 (0.02%) | 1 (0.02%) | 5 (0.02%) | 99.83% |
| **68** | Ecuador | 0 (0.00%) | 0 (0.00%) | 1 (0.03%) | 1 (0.02%) | 1 (0.02%) | 1 (0.02%) | 1 (0.02%) | 5 (0.02%) | 99.85% |
| **69** | Indonesia | 0 (0.00%) | 0 (0.00%) | 1 (0.03%) | 1 (0.02%) | 1 (0.02%) | 1 (0.02%) | 1 (0.02%) | 5 (0.02%) | 99.86% |
| **70** | Puerto Rico | 0 (0.00%) | 0 (0.00%) | 0 (0.00%) | 1 (0.02%) | 1 (0.02%) | 2 (0.04%) | 1 (0.02%) | 5 (0.02%) | 99.88% |
| **71** | Malta | 0 (0.00%) | 0 (0.00%) | 1 (0.03%) | 0 (0.00%) | 1 (0.02%) | 1 (0.02%) | 1 (0.02%) | 4 (0.01%) | 99.89% |
| **72** | Malawi | 0 (0.00%) | 1 (0.04%) | 1 (0.03%) | 1 (0.02%) | 0 (0.00%) | 1 (0.02%) | 0 (0.00%) | 4 (0.01%) | 99.90% |
| **73** | Nepal | 0 (0.00%) | 0 (0.00%) | 0 (0.00%) | 1 (0.02%) | 1 (0.02%) | 1 (0.02%) | 1 (0.02%) | 4 (0.01%) | 99.92% |
| **74** | Vanuatu | 0 (0.00%) | 0 (0.00%) | 0 (0.00%) | 1 (0.02%) | 1 (0.02%) | 1 (0.02%) | 1 (0.02%) | 4 (0.01%) | 99.93% |
| **75** | Georgia | 0 (0.00%) | 0 (0.00%) | 1 (0.03%) | 1 (0.02%) | 0 (0.00%) | 0 (0.00%) | 1 (0.02%) | 3 (0.01%) | 99.94% |
| **76** | Luxembourg | 0 (0.00%) | 1 (0.04%) | 1 (0.03%) | 1 (0.02%) | 0 (0.00%) | 0 (0.00%) | 0 (0.00%) | 3 (0.01%) | 99.95% |
| **77** | Viet Nam | 0 (0.00%) | 0 (0.00%) | 0 (0.00%) | 1 (0.02%) | 1 (0.02%) | 1 (0.02%) | 0 (0.00%) | 3 (0.01%) | 99.96% |
| **78** | Estonia | 0 (0.00%) | 0 (0.00%) | 0 (0.00%) | 0 (0.00%) | 1 (0.02%) | 0 (0.00%) | 1 (0.02%) | 2 (0.01%) | 99.97% |
| **79** | Ethiopia | 0 (0.00%) | 0 (0.00%) | 1 (0.03%) | 0 (0.00%) | 1 (0.02%) | 0 (0.00%) | 0 (0.00%) | 2 (0.01%) | 99.97% |
| **80** | Chile | 0 (0.00%) | 1 (0.04%) | 0 (0.00%) | 0 (0.00%) | 0 (0.00%) | 0 (0.00%) | 0 (0.00%) | 1 (0.00%) | 99.98% |
| **81** | Congo | 0 (0.00%) | 0 (0.00%) | 1 (0.03%) | 0 (0.00%) | 0 (0.00%) | 0 (0.00%) | 0 (0.00%) | 1 (0.00%) | 99.98% |
| **82** | Lebanon | 1 (0.04%) | 0 (0.00%) | 0 (0.00%) | 0 (0.00%) | 0 (0.00%) | 0 (0.00%) | 0 (0.00%) | 1 (0.00%) | 99.98% |
| **83** | Liberia | 0 (0.00%) | 0 (0.00%) | 0 (0.00%) | 0 (0.00%) | 0 (0.00%) | 1 (0.02%) | 0 (0.00%) | 1 (0.00%) | 99.99% |
| **84** | Morocco | 0 (0.00%) | 0 (0.00%) | 0 (0.00%) | 0 (0.00%) | 0 (0.00%) | 0 (0.00%) | 1 (0.02%) | 1 (0.00%) | 99.99% |
| **85** | Uganda | 0 (0.00%) | 0 (0.00%) | 0 (0.00%) | 0 (0.00%) | 1 (0.02%) | 0 (0.00%) | 0 (0.00%) | 1 (0.00%) | 99.99% |
| **86** | Ukraine | 0 (0.00%) | 0 (0.00%) | 0 (0.00%) | 0 (0.00%) | 1 (0.02%) | 0 (0.00%) | 0 (0.00%) | 1 (0.00%) | 100.00% |
| **87** | Yemen | 0 (0.00%) | 0 (0.00%) | 1 (0.03%) | 0 (0.00%) | 0 (0.00%) | 0 (0.00%) | 0 (0.00%) | 1 (0.00%) | 100.00% |
|  | *Missed* |  |  |  |  |  |  |  |  |  |
|  | Total |  |  |  |  |  |  |  |  |  |

**Table S2.** National-level Analysis: Distribution of Pharmacologic Scholars in the *Single-Year* Stanford-Elsevier Lists (SEL) of Top 2% Scientists Worldwide (2017–2023)

| **#** | **Country** | **SEL 2017** | **SEL 2019** | **SEL 2020** | **SEL 2021** | **SEL 2022** | **SEL 2023** | **Total ▼** | **Σ%** |
| --- | --- | --- | --- | --- | --- | --- | --- | --- | --- |
| **1** | United States of America | 627 (35.15%) | 1023 (29.06%) | 1202 (25.74%) | 1179 (24.62%) | 1155 (23.23%) | 1137 (21.95%) | 6323 (25.38%) | 25.38% |
| **2** | United Kingdom | 230 (12.89%) | 305 (8.66%) | 371 (7.94%) | 355 (7.41%) | 356 (7.16%) | 341 (6.58%) | 1958 (7.86%) | 33.24% |
| **3** | China | 45 (2.52%) | 203 (5.77%) | 311 (6.66%) | 331 (6.91%) | 383 (7.70%) | 432 (8.34%) | 1705 (6.84%) | 40.08% |
| **4** | Italy | 80 (4.48%) | 179 (5.09%) | 264 (5.65%) | 283 (5.91%) | 281 (5.65%) | 297 (5.73%) | 1384 (5.56%) | 45.64% |
| **5** | India | 31 (1.74%) | 131 (3.72%) | 253 (5.42%) | 263 (5.49%) | 321 (6.46%) | 381 (7.36%) | 1380 (5.54%) | 51.18% |
| **6** | Germany | 105 (5.89%) | 179 (5.09%) | 222 (4.75%) | 224 (4.68%) | 203 (4.08%) | 192 (3.71%) | 1125 (4.52%) | 55.7% |
| **7** | Japan | 77 (4.32%) | 151 (4.29%) | 185 (3.96%) | 178 (3.72%) | 178 (3.58%) | 161 (3.11%) | 930 (3.73%) | 59.43% |
| **8** | Iran | 12 (0.67%) | 78 (2.22%) | 148 (3.17%) | 166 (3.47%) | 196 (3.94%) | 229 (4.42%) | 829 (3.33%) | 62.76% |
| **9** | Canada | 80 (4.48%) | 113 (3.21%) | 134 (2.87%) | 133 (2.78%) | 134 (2.70%) | 130 (2.51%) | 724 (2.91%) | 65.67% |
| **10** | Australia | 65 (3.64%) | 113 (3.21%) | 140 (3.00%) | 134 (2.80%) | 131 (2.64%) | 129 (2.49%) | 712 (2.86%) | 68.53% |
| **11** | South Korea | 30 (1.68%) | 108 (3.07%) | 131 (2.81%) | 137 (2.86%) | 151 (3.04%) | 142 (2.74%) | 699 (2.81%) | 71.34% |
| **12** | France | 50 (2.80%) | 93 (2.64%) | 122 (2.61%) | 127 (2.65%) | 116 (2.33%) | 117 (2.26%) | 625 (2.51%) | 73.85% |
| **13** | Netherlands | 50 (2.80%) | 89 (2.53%) | 102 (2.18%) | 108 (2.26%) | 103 (2.07%) | 101 (1.95%) | 553 (2.22%) | 76.07% |
| **14** | Saudi Arabia | 3 (0.17%) | 28 (0.80%) | 68 (1.46%) | 84 (1.75%) | 115 (2.31%) | 147 (2.84%) | 445 (1.79%) | 77.86% |
| **15** | Spain | 23 (1.29%) | 50 (1.42%) | 67 (1.43%) | 82 (1.71%) | 79 (1.59%) | 80 (1.54%) | 381 (1.53%) | 79.39% |
| **16** | Egypt | 8 (0.45%) | 27 (0.77%) | 58 (1.24%) | 69 (1.44%) | 82 (1.65%) | 115 (2.22%) | 359 (1.44%) | 80.83% |
| **17** | Switzerland | 26 (1.46%) | 53 (1.51%) | 61 (1.31%) | 63 (1.32%) | 65 (1.31%) | 60 (1.16%) | 328 (1.32%) | 82.15% |
| **18** | Belgium | 28 (1.57%) | 50 (1.42%) | 62 (1.33%) | 56 (1.17%) | 52 (1.05%) | 50 (0.97%) | 298 (1.20%) | 83.35% |
| **19** | Brazil | 11 (0.62%) | 32 (0.91%) | 54 (1.16%) | 57 (1.19%) | 59 (1.19%) | 69 (1.33%) | 282 (1.13%) | 84.48% |
| **20** | New Zealand | 9 (0.50%) | 45 (1.28%) | 48 (1.03%) | 47 (0.98%) | 50 (1.01%) | 53 (1.02%) | 252 (1.01%) | 85.49% |
| **21** | Sweden | 32 (1.79%) | 40 (1.14%) | 47 (1.01%) | 43 (0.90%) | 43 (0.87%) | 47 (0.91%) | 252 (1.01%) | 86.5% |
| **22** | Denmark | 16 (0.90%) | 35 (0.99%) | 47 (1.01%) | 48 (1.00%) | 47 (0.95%) | 46 (0.89%) | 239 (0.96%) | 87.46% |
| **23** | Poland | 10 (0.56%) | 35 (0.99%) | 51 (1.09%) | 47 (0.98%) | 43 (0.87%) | 49 (0.95%) | 235 (0.94%) | 88.4% |
| **24** | Portugal | 6 (0.34%) | 22 (0.62%) | 35 (0.75%) | 42 (0.88%) | 46 (0.93%) | 53 (1.02%) | 204 (0.82%) | 89.22% |
| **25** | Austria | 15 (0.84%) | 26 (0.74%) | 34 (0.73%) | 35 (0.73%) | 30 (0.60%) | 31 (0.60%) | 171 (0.69%) | 89.91% |
| **26** | Pakistan | 3 (0.17%) | 10 (0.28%) | 23 (0.49%) | 34 (0.71%) | 49 (0.99%) | 49 (0.95%) | 168 (0.67%) | 90.58% |
| **27** | Taiwan | 15 (0.84%) | 24 (0.68%) | 32 (0.69%) | 31 (0.65%) | 28 (0.56%) | 30 (0.58%) | 160 (0.64%) | 91.22% |
| **28** | Finland | 14 (0.78%) | 27 (0.77%) | 31 (0.66%) | 32 (0.67%) | 26 (0.52%) | 29 (0.56%) | 159 (0.64%) | 91.86% |
| **29** | Israel | 15 (0.84%) | 22 (0.62%) | 27 (0.58%) | 30 (0.63%) | 26 (0.52%) | 29 (0.56%) | 149 (0.60%) | 92.46% |
| **30** | Malaysia | 1 (0.06%) | 15 (0.43%) | 31 (0.66%) | 27 (0.56%) | 29 (0.58%) | 40 (0.77%) | 143 (0.57%) | 93.03% |
| **31** | Ireland | 10 (0.56%) | 19 (0.54%) | 24 (0.51%) | 26 (0.54%) | 22 (0.44%) | 24 (0.46%) | 125 (0.50%) | 93.53% |
| **32** | Thailand | 3 (0.17%) | 15 (0.43%) | 24 (0.51%) | 23 (0.48%) | 27 (0.54%) | 30 (0.58%) | 122 (0.49%) | 94.02% |
| **33** | Greece | 7 (0.39%) | 13 (0.37%) | 21 (0.45%) | 25 (0.52%) | 23 (0.46%) | 23 (0.44%) | 112 (0.45%) | 94.47% |
| **34** | Singapore | 7 (0.39%) | 22 (0.62%) | 19 (0.41%) | 20 (0.42%) | 21 (0.42%) | 21 (0.41%) | 110 (0.44%) | 94.91% |
| **35** | Türkiye | 3 (0.17%) | 10 (0.28%) | 18 (0.39%) | 21 (0.44%) | 25 (0.50%) | 23 (0.44%) | 100 (0.40%) | 95.31% |
| **36** | Hong Kong | 6 (0.34%) | 13 (0.37%) | 16 (0.34%) | 17 (0.36%) | 20 (0.40%) | 13 (0.25%) | 85 (0.34%) | 95.65% |
| **37** | South Africa | 4 (0.22%) | 13 (0.37%) | 14 (0.30%) | 16 (0.33%) | 18 (0.36%) | 20 (0.39%) | 85 (0.34%) | 95.99% |
| **38** | Hungary | 6 (0.34%) | 11 (0.31%) | 16 (0.34%) | 14 (0.29%) | 16 (0.32%) | 16 (0.31%) | 79 (0.32%) | 96.31% |
| **39** | United Arab Emirates | 1 (0.06%) | 3 (0.09%) | 11 (0.24%) | 13 (0.27%) | 20 (0.40%) | 24 (0.46%) | 72 (0.29%) | 96.6% |
| **40** | Norway | 2 (0.11%) | 8 (0.23%) | 12 (0.26%) | 10 (0.21%) | 14 (0.28%) | 17 (0.33%) | 63 (0.25%) | 96.85% |
| **41** | Jordan | 0 (0.00%) | 5 (0.14%) | 8 (0.17%) | 10 (0.21%) | 12 (0.24%) | 19 (0.37%) | 54 (0.22%) | 97.07% |
| **42** | Czechia | 5 (0.28%) | 10 (0.28%) | 12 (0.26%) | 10 (0.21%) | 9 (0.18%) | 6 (0.12%) | 52 (0.21%) | 97.28% |
| **43** | Iraq | 0 (0.00%) | 8 (0.23%) | 12 (0.26%) | 8 (0.17%) | 11 (0.22%) | 12 (0.23%) | 51 (0.20%) | 97.48% |
| **44** | Mexico | 2 (0.11%) | 9 (0.26%) | 11 (0.24%) | 7 (0.15%) | 8 (0.16%) | 7 (0.14%) | 44 (0.18%) | 97.66% |
| **45** | Macao | 3 (0.17%) | 6 (0.17%) | 5 (0.11%) | 10 (0.21%) | 8 (0.16%) | 7 (0.14%) | 39 (0.16%) | 97.82% |
| **46** | State of Palestine | 0 (0.00%) | 5 (0.14%) | 7 (0.15%) | 8 (0.17%) | 10 (0.20%) | 9 (0.17%) | 39 (0.16%) | 97.98% |
| **47** | Romania | 1 (0.06%) | 1 (0.03%) | 7 (0.15%) | 5 (0.10%) | 12 (0.24%) | 11 (0.21%) | 37 (0.15%) | 98.13% |
| **48** | Nigeria | 0 (0.00%) | 0 (0.00%) | 3 (0.06%) | 6 (0.13%) | 12 (0.24%) | 13 (0.25%) | 34 (0.14%) | 98.27% |
| **49** | Qatar | 0 (0.00%) | 4 (0.11%) | 4 (0.09%) | 5 (0.10%) | 7 (0.14%) | 9 (0.17%) | 29 (0.12%) | 98.39% |
| **50** | Indonesia | 0 (0.00%) | 1 (0.03%) | 4 (0.09%) | 4 (0.08%) | 8 (0.16%) | 11 (0.21%) | 28 (0.11%) | 98.5% |
| **51** | Iceland | 2 (0.11%) | 4 (0.11%) | 3 (0.06%) | 5 (0.10%) | 7 (0.14%) | 6 (0.12%) | 27 (0.11%) | 98.61% |
| **52** | Russia | 0 (0.00%) | 5 (0.14%) | 3 (0.06%) | 7 (0.15%) | 5 (0.10%) | 7 (0.14%) | 27 (0.11%) | 98.72% |
| **53** | Slovenia | 0 (0.00%) | 4 (0.11%) | 4 (0.09%) | 5 (0.10%) | 5 (0.10%) | 5 (0.10%) | 23 (0.09%) | 98.81% |
| **54** | Bangladesh | 0 (0.00%) | 2 (0.06%) | 3 (0.06%) | 3 (0.06%) | 7 (0.14%) | 7 (0.14%) | 22 (0.09%) | 98.9% |
| **55** | Serbia | 0 (0.00%) | 2 (0.06%) | 5 (0.11%) | 5 (0.10%) | 5 (0.10%) | 5 (0.10%) | 22 (0.09%) | 98.99% |
| **56** | Croatia | 0 (0.00%) | 2 (0.06%) | 2 (0.04%) | 5 (0.10%) | 5 (0.10%) | 5 (0.10%) | 19 (0.08%) | 99.07% |
| **57** | Kuwait | 0 (0.00%) | 1 (0.03%) | 4 (0.09%) | 4 (0.08%) | 5 (0.10%) | 4 (0.08%) | 18 (0.07%) | 99.14% |
| **58** | Argentina | 0 (0.00%) | 2 (0.06%) | 2 (0.04%) | 3 (0.06%) | 4 (0.08%) | 6 (0.12%) | 17 (0.07%) | 99.21% |
| **59** | Oman | 1 (0.06%) | 1 (0.03%) | 3 (0.06%) | 3 (0.06%) | 4 (0.08%) | 4 (0.08%) | 16 (0.06%) | 99.27% |
| **60** | Viet Nam | 0 (0.00%) | 2 (0.06%) | 3 (0.06%) | 4 (0.08%) | 3 (0.06%) | 4 (0.08%) | 16 (0.06%) | 99.33% |
| **61** | Lebanon | 1 (0.06%) | 2 (0.06%) | 2 (0.04%) | 3 (0.06%) | 3 (0.06%) | 3 (0.06%) | 14 (0.06%) | 99.39% |
| **62** | Bahrain | 1 (0.06%) | 1 (0.03%) | 2 (0.04%) | 2 (0.04%) | 3 (0.06%) | 3 (0.06%) | 12 (0.05%) | 99.44% |
| **63** | Ethiopia | 0 (0.00%) | 0 (0.00%) | 1 (0.02%) | 3 (0.06%) | 2 (0.04%) | 5 (0.10%) | 11 (0.04%) | 99.48% |
| **64** | Bulgaria | 0 (0.00%) | 1 (0.03%) | 2 (0.04%) | 2 (0.04%) | 2 (0.04%) | 2 (0.04%) | 9 (0.04%) | 99.52% |
| **65** | Sri Lanka | 0 (0.00%) | 2 (0.06%) | 2 (0.04%) | 2 (0.04%) | 2 (0.04%) | 1 (0.02%) | 9 (0.04%) | 99.56% |
| **66** | Morocco | 0 (0.00%) | 0 (0.00%) | 2 (0.04%) | 2 (0.04%) | 2 (0.04%) | 3 (0.06%) | 9 (0.04%) | 99.6% |
| **67** | Ukraine | 0 (0.00%) | 0 (0.00%) | 2 (0.04%) | 6 (0.13%) | 1 (0.02%) | 0 (0.00%) | 9 (0.04%) | 99.64% |
| **68** | Malta | 0 (0.00%) | 1 (0.03%) | 0 (0.00%) | 2 (0.04%) | 2 (0.04%) | 3 (0.06%) | 8 (0.03%) | 99.67% |
| **69** | Cyprus | 0 (0.00%) | 0 (0.00%) | 0 (0.00%) | 1 (0.02%) | 3 (0.06%) | 2 (0.04%) | 6 (0.02%) | 99.69% |
| **70** | Lithuania | 0 (0.00%) | 0 (0.00%) | 1 (0.02%) | 1 (0.02%) | 2 (0.04%) | 2 (0.04%) | 6 (0.02%) | 99.71% |
| **71** | Nepal | 0 (0.00%) | 0 (0.00%) | 1 (0.02%) | 1 (0.02%) | 2 (0.04%) | 2 (0.04%) | 6 (0.02%) | 99.73% |
| **72** | Peru | 0 (0.00%) | 0 (0.00%) | 0 (0.00%) | 2 (0.04%) | 2 (0.04%) | 2 (0.04%) | 6 (0.02%) | 99.75% |
| **73** | Chile | 1 (0.06%) | 0 (0.00%) | 1 (0.02%) | 1 (0.02%) | 1 (0.02%) | 1 (0.02%) | 5 (0.02%) | 99.77% |
| **74** | Ecuador | 0 (0.00%) | 1 (0.03%) | 1 (0.02%) | 1 (0.02%) | 1 (0.02%) | 1 (0.02%) | 5 (0.02%) | 99.79% |
| **75** | Estonia | 0 (0.00%) | 0 (0.00%) | 1 (0.02%) | 2 (0.04%) | 0 (0.00%) | 2 (0.04%) | 5 (0.02%) | 99.81% |
| **76** | North Macedonia | 0 (0.00%) | 1 (0.03%) | 1 (0.02%) | 1 (0.02%) | 1 (0.02%) | 1 (0.02%) | 5 (0.02%) | 99.83% |
| **77** | Namibia | 0 (0.00%) | 1 (0.03%) | 1 (0.02%) | 1 (0.02%) | 1 (0.02%) | 1 (0.02%) | 5 (0.02%) | 99.85% |
| **78** | Slovakia | 0 (0.00%) | 0 (0.00%) | 1 (0.02%) | 0 (0.00%) | 2 (0.04%) | 2 (0.04%) | 5 (0.02%) | 99.87% |
| **79** | Uruguay | 1 (0.06%) | 0 (0.00%) | 1 (0.02%) | 1 (0.02%) | 1 (0.02%) | 1 (0.02%) | 5 (0.02%) | 99.89% |
| **80** | Latvia | 0 (0.00%) | 0 (0.00%) | 1 (0.02%) | 1 (0.02%) | 1 (0.02%) | 1 (0.02%) | 4 (0.02%) | 99.91% |
| **81** | Colombia | 0 (0.00%) | 0 (0.00%) | 0 (0.00%) | 1 (0.02%) | 1 (0.02%) | 1 (0.02%) | 3 (0.01%) | 99.92% |
| **82** | Malawi | 0 (0.00%) | 1 (0.03%) | 1 (0.02%) | 0 (0.00%) | 1 (0.02%) | 0 (0.00%) | 3 (0.01%) | 99.93% |
| **83** | Puerto Rico | 0 (0.00%) | 0 (0.00%) | 1 (0.02%) | 1 (0.02%) | 1 (0.02%) | 0 (0.00%) | 3 (0.01%) | 99.94% |
| **84** | Syrian Arab Republic | 0 (0.00%) | 0 (0.00%) | 0 (0.00%) | 1 (0.02%) | 1 (0.02%) | 1 (0.02%) | 3 (0.01%) | 99.95% |
| **85** | Trinidad and Tobago | 0 (0.00%) | 0 (0.00%) | 1 (0.02%) | 1 (0.02%) | 0 (0.00%) | 1 (0.02%) | 3 (0.01%) | 99.96% |
| **86** | Brunei Darussalam | 0 (0.00%) | 0 (0.00%) | 1 (0.02%) | 1 (0.02%) | 0 (0.00%) | 0 (0.00%) | 2 (0.01%) | 99.97% |
| **87** | Georgia | 0 (0.00%) | 1 (0.03%) | 1 (0.02%) | 0 (0.00%) | 0 (0.00%) | 0 (0.00%) | 2 (0.01%) | 99.98% |
| **88** | Kazakhstan | 0 (0.00%) | 0 (0.00%) | 0 (0.00%) | 0 (0.00%) | 1 (0.02%) | 1 (0.02%) | 2 (0.01%) | 99.99% |
| **89** | Luxembourg | 0 (0.00%) | 1 (0.03%) | 1 (0.02%) | 0 (0.00%) | 0 (0.00%) | 0 (0.00%) | 2 (0.01%) | 100% |
| **90** | Albania | 0 (0.00%) | 0 (0.00%) | 0 (0.00%) | 0 (0.00%) | 0 (0.00%) | 1 (0.02%) | 1 (0.00%) | 100% |
| **91** | Azerbaijan | 0 (0.00%) | 0 (0.00%) | 0 (0.00%) | 0 (0.00%) | 0 (0.00%) | 1 (0.02%) | 1 (0.00%) | 100% |
| **92** | Ghana | 0 (0.00%) | 0 (0.00%) | 0 (0.00%) | 0 (0.00%) | 1 (0.02%) | 0 (0.00%) | 1 (0.00%) | 100% |
| **93** | Liberia | 0 (0.00%) | 0 (0.00%) | 0 (0.00%) | 0 (0.00%) | 1 (0.02%) | 0 (0.00%) | 1 (0.00%) | 100% |
| **94** | Sudan | 0 (0.00%) | 0 (0.00%) | 0 (0.00%) | 0 (0.00%) | 0 (0.00%) | 1 (0.02%) | 1 (0.00%) | 100% |
| **95** | Tunisia | 0 (0.00%) | 0 (0.00%) | 0 (0.00%) | 1 (0.02%) | 0 (0.00%) | 0 (0.00%) | 1 (0.00%) | 100% |
| **96** | Zambia | 0 (0.00%) | 0 (0.00%) | 0 (0.00%) | 0 (0.00%) | 0 (0.00%) | 1 (0.02%) | 1 (0.00%) | 100% |
|  | *Missed* |  |  |  |  |  |  |  |  |
|  | Total |  |  |  |  |  |  |  |  |

**Table S3.** Scholar-level Analysis: Gender Stratification of Pharmacologic Scholars in the *Career-Long* Stanford-Elsevier Lists (SEL) of Top 2% Scientists Worldwide (2017–2023)

| **Country** | **SEL 2017** | **SEL 2018** | **SEL 2019** | **SEL 2020** | **SEL 2021** | **SEL 2022** | **SEL 2023** | **Total ▼** |
| --- | --- | --- | --- | --- | --- | --- | --- | --- |
| USA | 136 / 824 (14.2%) | 151 / 918 (14.1%) | 197 / 1105 (15.1%) | 255 / 1309 (16.3%) | 264 / 1339 (16.5%) | 273 / 1342 (16.9%) | 284 / 1382 (17.0%) | 1560 / 8219 (16.0%) |
| GBR | 39 / 266 (12.8%) | 45 / 294 (13.3%) | 54 / 327 (14.2%) | 75 / 386 (16.3%) | 70 / 392 (15.2%) | 79 / 393 (16.7%) | 84 / 406 (17.1%) | 446 / 2464 (15.3%) |
| JPN | 6 / 121 (4.7%) | 6 / 136 (4.2%) | 19 / 217 (8.1%) | 17 / 276 (5.8%) | 17 / 279 (5.7%) | 20 / 299 (6.3%) | 20 / 297 (6.3%) | 105 / 1625 (6.1%) |
| DEU | 15 / 133 (10.1%) | 18 / 147 (10.9%) | 34 / 182 (15.7%) | 42 / 224 (15.8%) | 42 / 230 (15.4%) | 42 / 233 (15.3%) | 41 / 254 (13.9%) | 234 / 1403 (14.3%) |
| ITA | 13 / 92 (12.4%) | 16 / 107 (13.0%) | 35 / 114 (23.5%) | 52 / 151 (25.6%) | 50 / 153 (24.6%) | 53 / 156 (25.4%) | 62 / 157 (28.3%) | 281 / 930 (23.2%) |
| CAN | 24 / 93 (20.5%) | 17 / 86 (16.5%) | 30 / 109 (21.6%) | 41 / 126 (24.6%) | 42 / 132 (24.1%) | 43 / 129 (25.0%) | 43 / 138 (23.8%) | 240 / 813 (22.8%) |
| FRA | 12 / 82 (12.8%) | 11 / 101 (9.8%) | 17 / 96 (15.0%) | 26 / 128 (16.9%) | 30 / 135 (18.2%) | 31 / 131 (19.1%) | 29 / 138 (17.4%) | 156 / 811 (16.1%) |
| AUS | 12 / 64 (15.8%) | 13 / 58 (18.3%) | 17 / 81 (17.3%) | 29 / 101 (22.3%) | 27 / 100 (21.3%) | 30 / 99 (23.3%) | 36 / 104 (25.7%) | 164 / 607 (21.3%) |
| NLD | 7 / 51 (12.1%) | 7 / 58 (10.8%) | 11 / 78 (12.4%) | 14 / 100 (12.3%) | 14 / 102 (12.1%) | 14 / 112 (11.1%) | 17 / 113 (13.1%) | 84 / 614 (12.0%) |
| CHN | 6 / 12 (33.3%) | 4 / 18 (18.2%) | 22 / 43 (33.8%) | 24 / 65 (27.0%) | 23 / 74 (23.7%) | 26 / 86 (23.2%) | 31 / 98 (24.0%) | 136 / 396 (25.6%) |
| SWE | 5 / 53 (8.6%) | 5 / 37 (11.9%) | 12 / 52 (18.8%) | 16 / 66 (19.5%) | 17 / 79 (17.7%) | 15 / 80 (15.8%) | 15 / 79 (16.0%) | 85 / 446 (16.0%) |
| KOR | 2 / 16 (11.1%) | 2 / 16 (11.1%) | 10 / 42 (19.2%) | 16 / 75 (17.6%) | 16 / 78 (17.0%) | 20 / 85 (19.0%) | 23 / 93 (19.8%) | 89 / 405 (18.0%) |
| CHE | 2 / 44 (4.3%) | 5 / 35 (12.5%) | 7 / 55 (11.3%) | 7 / 65 (9.7%) | 10 / 62 (13.9%) | 9 / 72 (11.1%) | 9 / 73 (11.0%) | 49 / 406 (10.8%) |
| IND | 0 / 8 (0.0%) | 2 / 11 (15.4%) | 6 / 29 (17.1%) | 13 / 57 (18.6%) | 18 / 59 (23.4%) | 23 / 79 (22.5%) | 27 / 93 (22.5%) | 89 / 336 (20.9%) |
| BEL | 4 / 27 (12.9%) | 5 / 32 (13.5%) | 10 / 41 (19.6%) | 10 / 61 (14.1%) | 10 / 58 (14.7%) | 9 / 59 (13.2%) | 10 / 63 (13.7%) | 58 / 341 (14.5%) |
| ESP | 0 / 23 (0.0%) | 2 / 25 (7.4%) | 7 / 37 (15.9%) | 11 / 42 (20.8%) | 13 / 54 (19.4%) | 18 / 58 (23.7%) | 20 / 61 (24.7%) | 71 / 300 (19.1%) |
| DNK | 2 / 20 (9.1%) | 3 / 20 (13.0%) | 6 / 32 (15.8%) | 9 / 47 (16.1%) | 10 / 46 (17.9%) | 12 / 50 (19.4%) | 14 / 51 (21.5%) | 56 / 266 (17.4%) |
| POL | 1 / 9 (10.0%) | 3 / 17 (15.0%) | 10 / 23 (30.3%) | 18 / 36 (33.3%) | 19 / 35 (35.2%) | 21 / 36 (36.8%) | 26 / 37 (41.3%) | 98 / 193 (33.7%) |
| NZL | 6 / 11 (35.3%) | 14 / 11 (56.0%) | 21 / 13 (61.8%) | 26 / 23 (53.1%) | 27 / 25 (51.9%) | 27 / 25 (51.9%) | 29 / 24 (54.7%) | 150 / 132 (53.2%) |
| FIN | 1 / 23 (4.2%) | 2 / 23 (8.0%) | 1 / 34 (2.9%) | 1 / 42 (2.3%) | 2 / 44 (4.3%) | 3 / 41 (6.8%) | 4 / 44 (8.3%) | 14 / 251 (5.3%) |
| IRN | 1 / 5 (16.7%) | 1 / 2 (33.3%) | 2 / 13 (13.3%) | 8 / 34 (19.0%) | 9 / 38 (19.1%) | 8 / 44 (15.4%) | 12 / 49 (19.7%) | 41 / 185 (18.1%) |
| BRA | 0 / 10 (0.0%) | 0 / 11 (0.0%) | 3 / 20 (13.0%) | 10 / 28 (26.3%) | 9 / 28 (24.3%) | 11 / 28 (28.2%) | 12 / 32 (27.3%) | 45 / 157 (22.3%) |
| ISR | 3 / 21 (12.5%) | 4 / 22 (15.4%) | 5 / 19 (20.8%) | 5 / 24 (17.2%) | 5 / 26 (16.1%) | 5 / 29 (14.7%) | 6 / 28 (17.6%) | 33 / 169 (16.3%) |
| AUT | 1 / 19 (5.0%) | 1 / 18 (5.3%) | 7 / 18 (28.0%) | 8 / 28 (22.2%) | 7 / 23 (23.3%) | 8 / 24 (25.0%) | 11 / 23 (32.4%) | 43 / 153 (21.9%) |
| HUN | 2 / 8 (20.0%) | 2 / 9 (18.2%) | 3 / 14 (17.6%) | 4 / 22 (15.4%) | 6 / 23 (20.7%) | 6 / 22 (21.4%) | 6 / 22 (21.4%) | 29 / 120 (19.5%) |
| IRL | 2 / 10 (16.7%) | 1 / 11 (8.3%) | 3 / 13 (18.8%) | 4 / 17 (19.0%) | 4 / 16 (20.0%) | 4 / 15 (21.1%) | 4 / 14 (22.2%) | 22 / 96 (18.6%) |
| EGY | 0 / 1 (0.0%) | 0 / 1 (0.0%) | 0 / 10 (0.0%) | 3 / 18 (14.3%) | 4 / 18 (18.2%) | 7 / 22 (24.1%) | 9 / 24 (27.3%) | 23 / 94 (19.7%) |
| SAU | *NA* | *NA* | 1 / 8 (11.1%) | 0 / 18 (0.0%) | 0 / 19 (0.0%) | 0 / 26 (0.0%) | 0 / 30 (0.0%) | 1 / 101 (1.0%) |
| PRT | 2 / 4 (33.3%) | 2 / 5 (28.6%) | 4 / 7 (36.4%) | 5 / 10 (33.3%) | 6 / 12 (33.3%) | 6 / 12 (33.3%) | 7 / 13 (35.0%) | 32 / 63 (33.7%) |
| SGP | 0 / 3 (0.0%) | 0 / 5 (0.0%) | 3 / 11 (21.4%) | 3 / 13 (18.8%) | 4 / 12 (25.0%) | 4 / 16 (20.0%) | 4 / 16 (20.0%) | 18 / 76 (19.1%) |
| NOR | 1 / 5 (16.7%) | 1 / 4 (20.0%) | 2 / 7 (22.2%) | 5 / 13 (27.8%) | 4 / 12 (25.0%) | 5 / 11 (31.2%) | 5 / 14 (26.3%) | 23 / 66 (25.8%) |
| TUR | 2 / 2 (50.0%) | 2 / 0 (100.0%) | 6 / 6 (50.0%) | 7 / 4 (63.6%) | 9 / 7 (56.2%) | 12 / 7 (63.2%) | 12 / 7 (63.2%) | 50 / 33 (60.2%) |
| GRC | 1 / 4 (20.0%) | 1 / 3 (25.0%) | 0 / 7 (0.0%) | 1 / 11 (8.3%) | 2 / 9 (18.2%) | 2 / 10 (16.7%) | 2 / 16 (11.1%) | 9 / 60 (13.0%) |
| CZE | 1 / 1 (50.0%) | 1 / 6 (14.3%) | 1 / 9 (10.0%) | 2 / 10 (16.7%) | 2 / 9 (18.2%) | 1 / 11 (8.3%) | 1 / 12 (7.7%) | 9 / 58 (13.4%) |
| HKG | 2 / 5 (28.6%) | 1 / 7 (12.5%) | 2 / 5 (28.6%) | 1 / 8 (11.1%) | 2 / 5 (28.6%) | 3 / 8 (27.3%) | 2 / 7 (22.2%) | 13 / 45 (22.4%) |
| MYS | *NA* | *NA* | 0 / 3 (0.0%) | 0 / 9 (0.0%) | 0 / 11 (0.0%) | 0 / 10 (0.0%) | 0 / 14 (0.0%) | 0 / 47 (0.0%) |
| THA | 0 / 1 (0.0%) | 0 / 1 (0.0%) | 3 / 2 (60.0%) | 3 / 4 (42.9%) | 4 / 5 (44.4%) | 5 / 6 (45.5%) | 5 / 8 (38.5%) | 20 / 27 (42.6%) |
| PAK | 1 / 1 (50.0%) | 1 / 2 (33.3%) | 1 / 3 (25.0%) | 1 / 8 (11.1%) | 1 / 7 (12.5%) | 1 / 6 (14.3%) | 1 / 6 (14.3%) | 7 / 33 (17.5%) |
| ZAF | 0 / 3 (0.0%) | 0 / 3 (0.0%) | 0 / 5 (0.0%) | 1 / 4 (20.0%) | 0 / 4 (0.0%) | 1 / 6 (14.3%) | 1 / 9 (10.0%) | 3 / 34 (8.1%) |
| MEX | 1 / 1 (50.0%) | 1 / 1 (50.0%) | 1 / 3 (25.0%) | 1 / 6 (14.3%) | 1 / 6 (14.3%) | 1 / 6 (14.3%) | 1 / 5 (16.7%) | 7 / 28 (20.0%) |
| NGA | *NA* | 0 / 1 (0.0%) | 0 / 2 (0.0%) | 1 / 4 (20.0%) | 0 / 5 (0.0%) | 1 / 5 (16.7%) | 3 / 6 (33.3%) | 5 / 23 (17.9%) |
| TWN | 1 / 0 (100.0%) | 0 / 1 (0.0%) | 0 / 2 (0.0%) | 0 / 4 (0.0%) | 0 / 6 (0.0%) | 0 / 7 (0.0%) | 0 / 7 (0.0%) | 1 / 27 (3.6%) |
| ARE | 0 / 2 (0.0%) | 0 / 1 (0.0%) | 1 / 2 (33.3%) | 1 / 1 (50.0%) | 1 / 2 (33.3%) | 1 / 4 (20.0%) | 2 / 6 (25.0%) | 6 / 18 (25.0%) |
| ISL | 0 / 2 (0.0%) | 0 / 2 (0.0%) | 0 / 2 (0.0%) | 0 / 2 (0.0%) | 1 / 3 (25.0%) | 1 / 4 (20.0%) | 1 / 4 (20.0%) | 3 / 19 (13.6%) |
| RUS | *NA* | 0 / 3 (0.0%) | 0 / 4 (0.0%) | 0 / 3 (0.0%) | 0 / 3 (0.0%) | 0 / 3 (0.0%) | 0 / 3 (0.0%) | 0 / 19 (0.0%) |
| KWT | 0 / 1 (0.0%) | *NA* | 0 / 1 (0.0%) | 0 / 3 (0.0%) | 0 / 4 (0.0%) | 0 / 3 (0.0%) | 0 / 3 (0.0%) | 0 / 15 (0.0%) |
| PSE | *NA* | *NA* | 0 / 2 (0.0%) | 0 / 2 (0.0%) | 1 / 2 (33.3%) | 1 / 2 (33.3%) | 2 / 2 (50.0%) | 4 / 10 (28.6%) |
| QAT | *NA* | 0 / 2 (0.0%) | 0 / 2 (0.0%) | 0 / 2 (0.0%) | 0 / 2 (0.0%) | 0 / 2 (0.0%) | 0 / 4 (0.0%) | 0 / 14 (0.0%) |
| JOR | *NA* | *NA* | 0 / 1 (0.0%) | 1 / 1 (50.0%) | 1 / 0 (100.0%) | 1 / 3 (25.0%) | 1 / 3 (25.0%) | 4 / 8 (33.3%) |
| BGD | *NA* | *NA* | *NA* | 0 / 1 (0.0%) | 0 / 3 (0.0%) | 0 / 3 (0.0%) | 0 / 4 (0.0%) | 0 / 11 (0.0%) |
| HRV | *NA* | *NA* | 0 / 1 (0.0%) | 0 / 2 (0.0%) | 0 / 2 (0.0%) | 1 / 2 (33.3%) | 1 / 2 (33.3%) | 2 / 9 (18.2%) |
| ROU | *NA* | *NA* | 1 / 0 (100.0%) | 1 / 0 (100.0%) | 1 / 1 (50.0%) | 1 / 2 (33.3%) | 1 / 2 (33.3%) | 5 / 5 (50.0%) |
| SRB | *NA* | *NA* | *NA* | 0 / 3 (0.0%) | 0 / 3 (0.0%) | 0 / 2 (0.0%) | 0 / 2 (0.0%) | 0 / 10 (0.0%) |
| SVK | 0 / 1 (0.0%) | 0 / 1 (0.0%) | *NA* | 0 / 2 (0.0%) | 0 / 2 (0.0%) | 0 / 2 (0.0%) | 0 / 2 (0.0%) | 0 / 10 (0.0%) |
| BHR | 0 / 1 (0.0%) | 0 / 1 (0.0%) | 0 / 1 (0.0%) | 0 / 1 (0.0%) | 0 / 1 (0.0%) | 0 / 2 (0.0%) | 0 / 2 (0.0%) | 0 / 9 (0.0%) |
| IRQ | *NA* | *NA* | *NA* | *NA* | 1 / 1 (50.0%) | 1 / 2 (33.3%) | 1 / 3 (25.0%) | 3 / 6 (33.3%) |
| URY | 0 / 1 (0.0%) | *NA* | *NA* | 0 / 2 (0.0%) | 0 / 2 (0.0%) | 0 / 2 (0.0%) | 0 / 2 (0.0%) | 0 / 9 (0.0%) |
| SVN | *NA* | *NA* | *NA* | 1 / 0 (100.0%) | 2 / 0 (100.0%) | 2 / 0 (100.0%) | 2 / 1 (66.7%) | 7 / 1 (87.5%) |
| CYP | *NA* | 0 / 1 (0.0%) | 0 / 1 (0.0%) | 0 / 1 (0.0%) | 0 / 1 (0.0%) | 0 / 2 (0.0%) | 0 / 1 (0.0%) | 0 / 7 (0.0%) |
| NAM | *NA* | 0 / 1 (0.0%) | 0 / 1 (0.0%) | 0 / 1 (0.0%) | 0 / 1 (0.0%) | 1 / 1 (50.0%) | 0 / 1 (0.0%) | 1 / 6 (14.3%) |
| ARG | *NA* | 0 / 2 (0.0%) | *NA* | 0 / 1 (0.0%) | 0 / 1 (0.0%) | 0 / 1 (0.0%) | 0 / 1 (0.0%) | 0 / 6 (0.0%) |
| VEN | *NA* | *NA* | 0 / 1 (0.0%) | 0 / 2 (0.0%) | 0 / 1 (0.0%) | 0 / 1 (0.0%) | 0 / 1 (0.0%) | 0 / 6 (0.0%) |
| BGR | *NA* | *NA* | 1 / 0 (100.0%) | 1 / 0 (100.0%) | 1 / 0 (100.0%) | 1 / 0 (100.0%) | 1 / 0 (100.0%) | 5 / 0 (100.0%) |
| PRI | *NA* | *NA* | *NA* | 0 / 1 (0.0%) | 0 / 1 (0.0%) | 0 / 2 (0.0%) | 0 / 1 (0.0%) | 0 / 5 (0.0%) |
| TTO | *NA* | *NA* | 0 / 1 (0.0%) | 0 / 1 (0.0%) | 0 / 1 (0.0%) | 0 / 1 (0.0%) | 0 / 1 (0.0%) | 0 / 5 (0.0%) |
| ECU | *NA* | *NA* | 0 / 1 (0.0%) | 0 / 1 (0.0%) | 0 / 1 (0.0%) | *NA* | 0 / 1 (0.0%) | 0 / 4 (0.0%) |
| IDN | *NA* | *NA* | *NA* | 0 / 1 (0.0%) | 0 / 1 (0.0%) | 0 / 1 (0.0%) | 0 / 1 (0.0%) | 0 / 4 (0.0%) |
| MLT | *NA* | *NA* | 0 / 1 (0.0%) | *NA* | 0 / 1 (0.0%) | 0 / 1 (0.0%) | 0 / 1 (0.0%) | 0 / 4 (0.0%) |
| VUT | *NA* | *NA* | *NA* | 0 / 1 (0.0%) | 0 / 1 (0.0%) | 0 / 1 (0.0%) | 0 / 1 (0.0%) | 0 / 4 (0.0%) |
| GEO | *NA* | *NA* | 0 / 1 (0.0%) | 0 / 1 (0.0%) | *NA* | *NA* | 1 / 0 (100.0%) | 1 / 2 (33.3%) |
| VNM | *NA* | *NA* | *NA* | 0 / 1 (0.0%) | 0 / 1 (0.0%) | 0 / 1 (0.0%) | *NA* | 0 / 3 (0.0%) |
| EST | *NA* | *NA* | *NA* | *NA* | 1 / 0 (100.0%) | *NA* | 1 / 0 (100.0%) | 2 / 0 (100.0%) |
| LUX | *NA* | 0 / 1 (0.0%) | *NA* | 0 / 1 (0.0%) | *NA* | *NA* | *NA* | 0 / 2 (0.0%) |
| MWI | *NA* | 0 / 1 (0.0%) | 0 / 1 (0.0%) | *NA* | *NA* | *NA* | *NA* | 0 / 2 (0.0%) |
| CHL | *NA* | 0 / 1 (0.0%) | *NA* | *NA* | *NA* | *NA* | *NA* | 0 / 1 (0.0%) |
| COG | *NA* | *NA* | 0 / 1 (0.0%) | *NA* | *NA* | *NA* | *NA* | 0 / 1 (0.0%) |
| ETH | *NA* | *NA* | 0 / 1 (0.0%) | *NA* | *NA* | *NA* | *NA* | 0 / 1 (0.0%) |
| LBN | 0 / 1 (0.0%) | *NA* | *NA* | *NA* | *NA* | *NA* | *NA* | 0 / 1 (0.0%) |
| LBR | *NA* | *NA* | *NA* | *NA* | *NA* | 0 / 1 (0.0%) | *NA* | 0 / 1 (0.0%) |
| MAC | *NA* | *NA* | 0 / 1 (0.0%) | *NA* | *NA* | *NA* | *NA* | 0 / 1 (0.0%) |
| MAR | *NA* | *NA* | *NA* | *NA* | *NA* | *NA* | 0 / 1 (0.0%) | 0 / 1 (0.0%) |
| OMN | *NA* | *NA* | *NA* | *NA* | *NA* | *NA* | 0 / 1 (0.0%) | 0 / 1 (0.0%) |
| UGA | *NA* | *NA* | *NA* | *NA* | 0 / 1 (0.0%) | *NA* | *NA* | 0 / 1 (0.0%) |
| UKR | *NA* | *NA* | *NA* | *NA* | 0 / 1 (0.0%) | *NA* | *NA* | 0 / 1 (0.0%) |
| YEM | *NA* | *NA* | 0 / 1 (0.0%) | *NA* | *NA* | *NA* | *NA* | 0 / 1 (0.0%) |
| *NA* | 49 / 346 (12.4%) | 1 / 4 (20.0%) | 6 / 12 (33.3%) | 7 / 12 (36.8%) | 7 / 7 (50.0%) | 5 / 10 (33.3%) | 7 / 28 (20.0%) | 82 / 419 (16.4%) |
| **Total** | 314 / 2085 (13.1%) | 350 / 2280 (13.3%) | 576 / 2925 (16.5%) | 780 / 3715 (17.4%) | 810 / 3821 (17.5%) | 870 / 3947 (18.1%) | 942 / 4124 (18.6%) | 4642 / 22897 (16.9%) |

Female / Male (Female/Both Genders*100)

**Table S4.** Scholar-level Analysis: Gender Stratification of Pharmacologic Scholars in the *Single-Year* Stanford-Elsevier Lists (SEL) of Top 2% Scientists Worldwide (2017–2023)

| **Country** | **SEL 2017** | **SEL 2019** | **SEL 2020** | **SEL 2021** | **SEL 2022** | **SEL 2023** | **Total ▼** |
| --- | --- | --- | --- | --- | --- | --- | --- |
| USA | 104 / 495 (17.4%) | 213 / 737 (22.4%) | 256 / 867 (22.8%) | 242 / 862 (21.9%) | 239 / 847 (22.0%) | 240 / 825 (22.5%) | 1294 / 4633 (21.8%) |
| GBR | 35 / 167 (17.3%) | 48 / 227 (17.5%) | 65 / 266 (19.6%) | 71 / 250 (22.1%) | 79 / 245 (24.4%) | 75 / 232 (24.4%) | 373 / 1387 (21.2%) |
| CHN | 13 / 32 (28.9%) | 60 / 137 (30.5%) | 94 / 205 (31.4%) | 91 / 229 (28.4%) | 112 / 260 (30.1%) | 128 / 292 (30.5%) | 498 / 1155 (30.1%) |
| ITA | 16 / 63 (20.3%) | 61 / 110 (35.7%) | 92 / 154 (37.4%) | 111 / 161 (40.8%) | 108 / 159 (40.4%) | 119 / 163 (42.2%) | 507 / 810 (38.5%) |
| IND | 2 / 22 (8.3%) | 25 / 87 (22.3%) | 42 / 180 (18.9%) | 47 / 187 (20.1%) | 61 / 225 (21.3%) | 69 / 285 (19.5%) | 246 / 986 (20.0%) |
| DEU | 16 / 83 (16.2%) | 34 / 131 (20.6%) | 43 / 160 (21.2%) | 44 / 164 (21.2%) | 40 / 149 (21.2%) | 37 / 147 (20.1%) | 214 / 834 (20.4%) |
| JPN | 4 / 71 (5.3%) | 9 / 135 (6.2%) | 11 / 171 (6.0%) | 9 / 167 (5.1%) | 13 / 162 (7.4%) | 11 / 148 (6.9%) | 57 / 854 (6.3%) |
| IRN | 2 / 10 (16.7%) | 13 / 63 (17.1%) | 35 / 110 (24.1%) | 44 / 121 (26.7%) | 51 / 140 (26.7%) | 70 / 156 (31.0%) | 215 / 600 (26.4%) |
| CAN | 20 / 56 (26.3%) | 32 / 67 (32.3%) | 41 / 86 (32.3%) | 39 / 87 (31.0%) | 43 / 85 (33.6%) | 35 / 85 (29.2%) | 210 / 466 (31.1%) |
| KOR | 10 / 18 (35.7%) | 30 / 72 (29.4%) | 35 / 91 (27.8%) | 35 / 92 (27.6%) | 35 / 108 (24.5%) | 33 / 100 (24.8%) | 178 / 481 (27.0%) |
| AUS | 9 / 51 (15.0%) | 33 / 69 (32.4%) | 45 / 84 (34.9%) | 42 / 80 (34.4%) | 39 / 77 (33.6%) | 39 / 81 (32.5%) | 207 / 442 (31.9%) |
| FRA | 8 / 39 (17.0%) | 17 / 64 (21.0%) | 23 / 83 (21.7%) | 23 / 88 (20.7%) | 22 / 83 (21.0%) | 22 / 84 (20.8%) | 115 / 441 (20.7%) |
| NLD | 9 / 37 (19.6%) | 17 / 64 (21.0%) | 20 / 74 (21.3%) | 21 / 77 (21.4%) | 23 / 76 (23.2%) | 21 / 75 (21.9%) | 111 / 403 (21.6%) |
| SAU | 0 / 3 (0.0%) | 2 / 24 (7.7%) | 3 / 63 (4.5%) | 7 / 74 (8.6%) | 8 / 104 (7.1%) | 17 / 124 (12.1%) | 37 / 392 (8.6%) |
| ESP | 2 / 18 (10.0%) | 12 / 35 (25.5%) | 24 / 38 (38.7%) | 25 / 51 (32.9%) | 24 / 52 (31.6%) | 22 / 55 (28.6%) | 109 / 249 (30.4%) |
| EGY | 1 / 7 (12.5%) | 8 / 19 (29.6%) | 23 / 33 (41.1%) | 30 / 38 (44.1%) | 33 / 48 (40.7%) | 46 / 69 (40.0%) | 141 / 214 (39.7%) |
| CHE | 2 / 22 (8.3%) | 3 / 43 (6.5%) | 4 / 53 (7.0%) | 9 / 48 (15.8%) | 7 / 52 (11.9%) | 9 / 47 (16.1%) | 34 / 265 (11.4%) |
| BEL | 5 / 18 (21.7%) | 12 / 35 (25.5%) | 15 / 42 (26.3%) | 14 / 37 (27.5%) | 11 / 39 (22.0%) | 10 / 37 (21.3%) | 67 / 208 (24.4%) |
| BRA | 0 / 10 (0.0%) | 8 / 22 (26.7%) | 20 / 31 (39.2%) | 18 / 34 (34.6%) | 20 / 35 (36.4%) | 23 / 40 (36.5%) | 89 / 172 (34.1%) |
| SWE | 5 / 27 (15.6%) | 10 / 26 (27.8%) | 14 / 29 (32.6%) | 12 / 29 (29.3%) | 10 / 29 (25.6%) | 10 / 33 (23.3%) | 61 / 173 (26.1%) |
| NZL | 3 / 6 (33.3%) | 27 / 14 (65.9%) | 27 / 16 (62.8%) | 26 / 18 (59.1%) | 27 / 19 (58.7%) | 28 / 20 (58.3%) | 138 / 93 (59.7%) |
| POL | 1 / 8 (11.1%) | 13 / 19 (40.6%) | 22 / 27 (44.9%) | 17 / 29 (37.0%) | 15 / 25 (37.5%) | 23 / 25 (47.9%) | 91 / 133 (40.6%) |
| DNK | 2 / 13 (13.3%) | 5 / 27 (15.6%) | 9 / 33 (21.4%) | 11 / 31 (26.2%) | 13 / 31 (29.5%) | 15 / 27 (35.7%) | 55 / 162 (25.3%) |
| PRT | 2 / 4 (33.3%) | 7 / 13 (35.0%) | 13 / 21 (38.2%) | 19 / 20 (48.7%) | 25 / 18 (58.1%) | 30 / 20 (60.0%) | 96 / 96 (50.0%) |
| AUT | 1 / 14 (6.7%) | 8 / 17 (32.0%) | 9 / 25 (26.5%) | 8 / 23 (25.8%) | 8 / 21 (27.6%) | 8 / 21 (27.6%) | 42 / 121 (25.8%) |
| PAK | 0 / 3 (0.0%) | 2 / 7 (22.2%) | 3 / 20 (13.0%) | 3 / 28 (9.7%) | 6 / 38 (13.6%) | 8 / 40 (16.7%) | 22 / 136 (13.9%) |
| FIN | 1 / 13 (7.1%) | 5 / 22 (18.5%) | 4 / 25 (13.8%) | 8 / 23 (25.8%) | 6 / 19 (24.0%) | 9 / 19 (32.1%) | 33 / 121 (21.4%) |
| ISR | 3 / 12 (20.0%) | 5 / 17 (22.7%) | 6 / 21 (22.2%) | 6 / 24 (20.0%) | 6 / 20 (23.1%) | 6 / 22 (21.4%) | 32 / 116 (21.6%) |
| MYS | 0 / 1 (0.0%) | 1 / 11 (8.3%) | 6 / 22 (21.4%) | 4 / 21 (16.0%) | 4 / 24 (14.3%) | 7 / 30 (18.9%) | 22 / 109 (16.8%) |
| IRL | 1 / 9 (10.0%) | 6 / 12 (33.3%) | 6 / 18 (25.0%) | 8 / 18 (30.8%) | 8 / 14 (36.4%) | 8 / 16 (33.3%) | 37 / 87 (29.8%) |
| SGP | 1 / 6 (14.3%) | 3 / 18 (14.3%) | 4 / 15 (21.1%) | 4 / 14 (22.2%) | 5 / 15 (25.0%) | 4 / 17 (19.0%) | 21 / 85 (19.8%) |
| GRC | 1 / 5 (16.7%) | 1 / 10 (9.1%) | 4 / 14 (22.2%) | 4 / 20 (16.7%) | 5 / 18 (21.7%) | 6 / 16 (27.3%) | 21 / 83 (20.2%) |
| TUR | 1 / 2 (33.3%) | 7 / 3 (70.0%) | 12 / 5 (70.6%) | 12 / 9 (57.1%) | 13 / 11 (54.2%) | 13 / 9 (59.1%) | 58 / 39 (59.8%) |
| THA | 1 / 1 (50.0%) | 6 / 6 (50.0%) | 9 / 9 (50.0%) | 9 / 8 (52.9%) | 12 / 9 (57.1%) | 11 / 11 (50.0%) | 48 / 44 (52.2%) |
| HUN | 1 / 4 (20.0%) | 2 / 8 (20.0%) | 4 / 11 (26.7%) | 3 / 9 (25.0%) | 3 / 11 (21.4%) | 3 / 11 (21.4%) | 16 / 54 (22.9%) |
| ZAF | 1 / 2 (33.3%) | 2 / 9 (18.2%) | 2 / 8 (20.0%) | 1 / 11 (8.3%) | 2 / 12 (14.3%) | 3 / 12 (20.0%) | 11 / 54 (16.9%) |
| ARE | 0 / 1 (0.0%) | 1 / 2 (33.3%) | 3 / 7 (30.0%) | 3 / 8 (27.3%) | 5 / 12 (29.4%) | 6 / 14 (30.0%) | 18 / 44 (29.0%) |
| NOR | 2 / 0 (100.0%) | 5 / 2 (71.4%) | 7 / 5 (58.3%) | 5 / 3 (62.5%) | 9 / 4 (69.2%) | 9 / 6 (60.0%) | 37 / 20 (64.9%) |
| JOR | *NA* | 2 / 3 (40.0%) | 4 / 4 (50.0%) | 6 / 3 (66.7%) | 5 / 7 (41.7%) | 8 / 11 (42.1%) | 25 / 28 (47.2%) |
| CZE | 1 / 4 (20.0%) | 2 / 8 (20.0%) | 3 / 9 (25.0%) | 4 / 6 (40.0%) | 2 / 7 (22.2%) | 0 / 6 (0.0%) | 12 / 40 (23.1%) |
| IRQ | *NA* | 1 / 6 (14.3%) | 1 / 11 (8.3%) | 1 / 6 (14.3%) | 2 / 9 (18.2%) | 2 / 10 (16.7%) | 7 / 42 (14.3%) |
| HKG | 1 / 4 (20.0%) | 3 / 5 (37.5%) | 2 / 8 (20.0%) | 3 / 5 (37.5%) | 3 / 7 (30.0%) | 1 / 5 (16.7%) | 13 / 34 (27.7%) |
| MEX | 1 / 1 (50.0%) | 3 / 5 (37.5%) | 1 / 10 (9.1%) | 1 / 6 (14.3%) | 1 / 6 (14.3%) | 1 / 6 (14.3%) | 8 / 34 (19.0%) |
| ROU | 0 / 1 (0.0%) | 1 / 0 (100.0%) | 6 / 1 (85.7%) | 5 / 0 (100.0%) | 8 / 4 (66.7%) | 8 / 3 (72.7%) | 28 / 9 (75.7%) |
| PSE | *NA* | 2 / 3 (40.0%) | 2 / 4 (33.3%) | 3 / 4 (42.9%) | 3 / 6 (33.3%) | 2 / 6 (25.0%) | 12 / 23 (34.3%) |
| NGA | *NA* | *NA* | 1 / 1 (50.0%) | 0 / 6 (0.0%) | 1 / 10 (9.1%) | 1 / 12 (7.7%) | 3 / 29 (9.4%) |
| QAT | *NA* | 0 / 4 (0.0%) | 0 / 4 (0.0%) | 0 / 4 (0.0%) | 0 / 7 (0.0%) | 0 / 9 (0.0%) | 0 / 28 (0.0%) |
| ISL | 0 / 2 (0.0%) | 0 / 4 (0.0%) | 0 / 3 (0.0%) | 1 / 3 (25.0%) | 2 / 4 (33.3%) | 0 / 4 (0.0%) | 3 / 20 (13.0%) |
| SVN | *NA* | 4 / 0 (100.0%) | 4 / 0 (100.0%) | 4 / 1 (80.0%) | 3 / 2 (60.0%) | 4 / 1 (80.0%) | 19 / 4 (82.6%) |
| BGD | *NA* | 1 / 1 (50.0%) | 0 / 3 (0.0%) | 0 / 3 (0.0%) | 0 / 7 (0.0%) | 0 / 7 (0.0%) | 1 / 21 (4.5%) |
| SRB | *NA* | 0 / 2 (0.0%) | 1 / 4 (20.0%) | 2 / 3 (40.0%) | 3 / 2 (60.0%) | 3 / 2 (60.0%) | 9 / 13 (40.9%) |
| IDN | *NA* | *NA* | 2 / 2 (50.0%) | 2 / 2 (50.0%) | 3 / 3 (50.0%) | 2 / 5 (28.6%) | 9 / 12 (42.9%) |
| TWN | 1 / 1 (50.0%) | 0 / 1 (0.0%) | 0 / 3 (0.0%) | 0 / 4 (0.0%) | 0 / 5 (0.0%) | 0 / 6 (0.0%) | 1 / 20 (4.8%) |
| HRV | *NA* | 0 / 2 (0.0%) | 0 / 2 (0.0%) | 1 / 4 (20.0%) | 1 / 4 (20.0%) | 1 / 4 (20.0%) | 3 / 16 (15.8%) |
| RUS | *NA* | 2 / 2 (50.0%) | 1 / 2 (33.3%) | 1 / 2 (33.3%) | 1 / 2 (33.3%) | 1 / 4 (20.0%) | 6 / 12 (33.3%) |
| ARG | *NA* | 0 / 2 (0.0%) | 0 / 2 (0.0%) | 1 / 2 (33.3%) | 2 / 2 (50.0%) | 4 / 2 (66.7%) | 7 / 10 (41.2%) |
| VNM | *NA* | 1 / 1 (50.0%) | 1 / 2 (33.3%) | 2 / 2 (50.0%) | 1 / 2 (33.3%) | 2 / 2 (50.0%) | 7 / 9 (43.8%) |
| MAC | 1 / 0 (100.0%) | 1 / 2 (33.3%) | 1 / 1 (50.0%) | 1 / 1 (50.0%) | 3 / 1 (75.0%) | 2 / 1 (66.7%) | 9 / 6 (60.0%) |
| LBN | 0 / 1 (0.0%) | 1 / 1 (50.0%) | 1 / 1 (50.0%) | 1 / 2 (33.3%) | 1 / 2 (33.3%) | 1 / 2 (33.3%) | 5 / 9 (35.7%) |
| BHR | 0 / 1 (0.0%) | 0 / 1 (0.0%) | 0 / 2 (0.0%) | 0 / 2 (0.0%) | 0 / 3 (0.0%) | 0 / 3 (0.0%) | 0 / 12 (0.0%) |
| ETH | *NA* | *NA* | 0 / 1 (0.0%) | 0 / 2 (0.0%) | 0 / 2 (0.0%) | 0 / 5 (0.0%) | 0 / 10 (0.0%) |
| KWT | *NA* | 0 / 1 (0.0%) | 0 / 2 (0.0%) | 0 / 2 (0.0%) | 0 / 2 (0.0%) | 0 / 2 (0.0%) | 0 / 9 (0.0%) |
| LKA | *NA* | 1 / 1 (50.0%) | 1 / 1 (50.0%) | 1 / 1 (50.0%) | 1 / 1 (50.0%) | 0 / 1 (0.0%) | 4 / 5 (44.4%) |
| MAR | *NA* | *NA* | 0 / 2 (0.0%) | 0 / 2 (0.0%) | 0 / 2 (0.0%) | 0 / 3 (0.0%) | 0 / 9 (0.0%) |
| OMN | *NA* | *NA* | 0 / 2 (0.0%) | 0 / 2 (0.0%) | 0 / 2 (0.0%) | 0 / 3 (0.0%) | 0 / 9 (0.0%) |
| MLT | *NA* | 0 / 1 (0.0%) | *NA* | 1 / 1 (50.0%) | 0 / 2 (0.0%) | 1 / 2 (33.3%) | 2 / 6 (25.0%) |
| UKR | *NA* | *NA* | 1 / 1 (50.0%) | 4 / 1 (80.0%) | 1 / 0 (100.0%) | *NA* | 6 / 2 (75.0%) |
| BGR | *NA* | 1 / 0 (100.0%) | 2 / 0 (100.0%) | 1 / 0 (100.0%) | 2 / 0 (100.0%) | 1 / 0 (100.0%) | 7 / 0 (100.0%) |
| LTU | *NA* | *NA* | 1 / 0 (100.0%) | 1 / 0 (100.0%) | 2 / 0 (100.0%) | 2 / 0 (100.0%) | 6 / 0 (100.0%) |
| PER | *NA* | *NA* | *NA* | 0 / 2 (0.0%) | 0 / 2 (0.0%) | 0 / 2 (0.0%) | 0 / 6 (0.0%) |
| CHL | 0 / 1 (0.0%) | *NA* | 0 / 1 (0.0%) | 0 / 1 (0.0%) | 0 / 1 (0.0%) | 0 / 1 (0.0%) | 0 / 5 (0.0%) |
| EST | *NA* | *NA* | 0 / 1 (0.0%) | 1 / 1 (50.0%) | *NA* | 1 / 1 (50.0%) | 2 / 3 (40.0%) |
| MKD | *NA* | 1 / 0 (100.0%) | 1 / 0 (100.0%) | 1 / 0 (100.0%) | 1 / 0 (100.0%) | 1 / 0 (100.0%) | 5 / 0 (100.0%) |
| NAM | *NA* | 0 / 1 (0.0%) | 0 / 1 (0.0%) | 0 / 1 (0.0%) | 0 / 1 (0.0%) | 0 / 1 (0.0%) | 0 / 5 (0.0%) |
| SVK | *NA* | *NA* | 0 / 1 (0.0%) | *NA* | 2 / 0 (100.0%) | 2 / 0 (100.0%) | 4 / 1 (80.0%) |
| URY | 0 / 1 (0.0%) | *NA* | 0 / 1 (0.0%) | 0 / 1 (0.0%) | 0 / 1 (0.0%) | 0 / 1 (0.0%) | 0 / 5 (0.0%) |
| ECU | *NA* | 0 / 1 (0.0%) | 0 / 1 (0.0%) | 0 / 1 (0.0%) | *NA* | 0 / 1 (0.0%) | 0 / 4 (0.0%) |
| LVA | *NA* | *NA* | 1 / 0 (100.0%) | 1 / 0 (100.0%) | 1 / 0 (100.0%) | 1 / 0 (100.0%) | 4 / 0 (100.0%) |
| COL | *NA* | *NA* | *NA* | 0 / 1 (0.0%) | 0 / 1 (0.0%) | 0 / 1 (0.0%) | 0 / 3 (0.0%) |
| CYP | *NA* | *NA* | *NA* | *NA* | 0 / 2 (0.0%) | 0 / 1 (0.0%) | 0 / 3 (0.0%) |
| PRI | *NA* | *NA* | 0 / 1 (0.0%) | 0 / 1 (0.0%) | 0 / 1 (0.0%) | *NA* | 0 / 3 (0.0%) |
| SYR | *NA* | *NA* | *NA* | 0 / 1 (0.0%) | 0 / 1 (0.0%) | 0 / 1 (0.0%) | 0 / 3 (0.0%) |
| TTO | *NA* | *NA* | 0 / 1 (0.0%) | 0 / 1 (0.0%) | *NA* | 0 / 1 (0.0%) | 0 / 3 (0.0%) |
| BRN | *NA* | *NA* | 0 / 1 (0.0%) | 0 / 1 (0.0%) | *NA* | *NA* | 0 / 2 (0.0%) |
| GEO | *NA* | 0 / 1 (0.0%) | 0 / 1 (0.0%) | *NA* | *NA* | *NA* | 0 / 2 (0.0%) |
| NPL | *NA* | *NA* | *NA* | *NA* | 0 / 1 (0.0%) | 0 / 1 (0.0%) | 0 / 2 (0.0%) |
| ALB | *NA* | *NA* | *NA* | *NA* | *NA* | 1 / 0 (100.0%) | 1 / 0 (100.0%) |
| AZE | *NA* | *NA* | *NA* | *NA* | *NA* | 0 / 1 (0.0%) | 0 / 1 (0.0%) |
| GHA | *NA* | *NA* | *NA* | *NA* | 0 / 1 (0.0%) | *NA* | 0 / 1 (0.0%) |
| LBR | *NA* | *NA* | *NA* | *NA* | 0 / 1 (0.0%) | *NA* | 0 / 1 (0.0%) |
| LUX | *NA* | *NA* | 0 / 1 (0.0%) | *NA* | *NA* | *NA* | 0 / 1 (0.0%) |
| MWI | *NA* | 0 / 1 (0.0%) | *NA* | *NA* | *NA* | *NA* | 0 / 1 (0.0%) |
| SDN | *NA* | *NA* | *NA* | *NA* | *NA* | 1 / 0 (100.0%) | 1 / 0 (100.0%) |
| TUN | *NA* | *NA* | *NA* | 0 / 1 (0.0%) | *NA* | *NA* | 0 / 1 (0.0%) |
| *NA* | 42 / 213 (16.5%) | 6 / 21 (22.2%) | 6 / 11 (35.3%) | 3 / 3 (50.0%) | 4 / 4 (50.0%) | 4 / 8 (33.3%) | 65 / 260 (20.0%) |
| **Total** | 290 / 1370 (17.5%) | 780 / 2444 (24.2%) | 1093 / 3221 (25.3%) | 1135 / 3288 (25.7%) | 1205 / 3422 (26.0%) | 1287 / 3554 (26.6%) | 5790 / 17299 (25.1%) |

Female / Male (Female/Both Genders*100)

**Table S5.** Scholar-Level Analysis: Gender Stratification of Scholarly Output Metrics of Pharmacologic Scholars in the Stanford-Elsevier Lists (SEL) of Top 2% Scientists Worldwide (2017–2023)

| ***Career-Long*** | | | | | | | | | | | | |
| --- | --- | --- | --- | --- | --- | --- | --- | --- | --- | --- | --- | --- |
| **Variable** | **Outcome** | **Female** | | | **Male** | | | ***p.*** | | | | |
|  |  | **C-Score:**  **Median (IQR)** | **Modified H:**  **Median (IQR)** | **Self-citations:**  **Median (IQR)** | **C-Score:**  **Median (IQR)** | **Modified H:**  **Median (IQR)** | **Self-citations:**  **Median (IQR)** | **C-Score** | | **Modified H-index** | | **Self-citations** |
| **Year** | SEL 2017 | 3.6 (3.5–3.8) | 18.1 (15.1–21.9) | 0.1 (0.1–0.2) | 3.7 (3.5–3.9) | 19.2 (15.9–24.3) | 0.1 (0.1–0.2) | **<0.001** | | **<0.001** | | 0.156 |
|  | SEL 2018 | 3.5 (3.4–3.7) | 19 (15.9–22.9) | 0.1 (0.1–0.2) | 3.6 (3.4–3.8) | 20.8 (17–25.8) | 0.1 (0.1–0.2) | **<0.001** | | **<0.001** | | 0.502 |
|  | SEL 2019 | 3.5 (3.3–3.6) | 16.3 (13.5–20.6) | 0.1 (0.1–0.2) | 3.6 (3.4–3.8) | 18.2 (14.6–23.7) | 0.1 (0.1–0.2) | **<0.001** | | **<0.001** | | 0.149 |
|  | SEL 2020 | 3.4 (3.2–3.6) | 16.3 (12.9–20.5) | 0.1 (0.1–0.2) | 3.5 (3.3–3.7) | 17.8 (13.9–23.1) | 0.1 (0.1–0.2) | **<0.001** | | **<0.001** | | 0.076 |
|  | SEL 2021 | 3.4 (3.2–3.6) | 16.4 (12.9–20.6) | 0.1 (0.1–0.2) | 3.5 (3.2–3.7) | 17.8 (13.9–23.5) | 0.1 (0.1–0.2) | **<0.001** | | **<0.001** | | 0.190 |
|  | SEL 2022 | 3.3 (3.2–3.6) | 16.5 (12.9–20.9) | 0.1 (0.1–0.2) | 3.5 (3.2–3.7) | 18 (14.2–23.9) | 0.1 (0.1–0.2) | **<0.001** | | **<0.001** | | 0.393 |
|  | SEL 2023 | 3.3 (3.2–3.6) | 16.7 (13.2–21) | 0.1 (0.1–0.2) | 3.4 (3.2–3.7) | 18.1 (14.2–24) | 0.1 (0.1–0.2) | **<0.001** | | **<0.001** | | 0.351 |
| **World Bank** | High | 3.4 (3.2–3.6) | 16.9 (13.5–21.2) | 0.1 (0.1–0.2) | 3.5 (3.3–3.8) | 18.6 (14.7–24.1) | 0.1 (0.1–0.2) | **<0.001** | | **<0.001** | | **<0.001** |
|  | Upper-middle | 3.2 (3.1–3.4) | 16.6 (12.9–20.1) | 0.1 (0.1–0.2) | 3.3 (3.2–3.5) | 17.9 (14.6–22.2) | 0.2 (0.1–0.2) | **<0.001** | | **<0.001** | | **<0.001** |
|  | Lower-middle | 3.2 (3.1–3.3) | 15.5 (13.2–19.2) | 0.1 (0.1–0.1) | 3.3 (3.2–3.5) | 16.7 (12.9–20.3) | 0.1 (0.1–0.2) | **<0.001** | | 0.209 | | **0.013** |
|  | Low | *NA* | *NA* | *NA* | 3.4 (3.2–3.6) | 13.3 (10.6–15.7) | 0.1 (0.1–0.1) | *NA* | | *NA* | | *NA* |
| **WHO Region** | EURO | 3.4 (3.2–3.6) | 16.7 (13.3–20.9) | 0.1 (0.1–0.2) | 3.5 (3.3–3.8) | 18.7 (14.8–24) | 0.1 (0.1–0.2) | **<0.001** | | **<0.001** | | **<0.001** |
|  | AMRO | 3.5 (3.3–3.7) | 16.7 (13.1–21.6) | 0.1 (0.1–0.1) | 3.6 (3.3–3.8) | 18.6 (14.4–24.8) | 0.1 (0.1–0.1) | **<0.001** | | **<0.001** | | **<0.001** |
|  | WPRO | 3.3 (3.2–3.6) | 17.7 (14.4–21.8) | 0.1 (0.1–0.2) | 3.4 (3.2–3.6) | 18.1 (14.9–23) | 0.1 (0.1–0.2) | **<0.001** | | **<0.001** | | **<0.001** |
|  | EMRO | 3.2 (3.1–3.4) | 16.1 (13.3–18.9) | 0.1 (0.1–0.2) | 3.3 (3.2–3.5) | 15.1 (12.5–19) | 0.2 (0.1–0.2) | **0.035** | | 0.515 | | **0.008** |
|  | SEARO | 3.2 (3.2–3.3) | 15.9 (13.9–19.3) | 0.1 (0.1–0.1) | 3.3 (3.2–3.5) | 17.9 (14.1–21.9) | 0.1 (0.1–0.2) | **<0.001** | | **0.007** | | **0.046** |
|  | AFRO | 3.3 (3.2–3.5) | 14.2 (12.8–14.8) | 0.1 (0.1–0.2) | 3.4 (3.2–3.6) | 15 (11.8–17.9) | 0.1 (0.1–0.1) | 0.281 | | 0.434 | | **0.023** |
| **Official Language** | English | 3.5 (3.3–3.7) | 16.9 (13.5–21.6) | 0.1 (0.1–0.1) | 3.6 (3.3–3.8) | 18.7 (14.6–24.8) | 0.1 (0.1–0.1) | **<0.001** | | **<0.001** | | **<0.001** |
|  | German | 3.5 (3.3–3.7) | 15.8 (12.9–20.7) | 0.1 (0.1–0.2) | 3.5 (3.3–3.8) | 18.8 (14.8–25) | 0.1 (0.1–0.2) | **<0.001** | | **<0.001** | | 0.714 |
|  | Japanese | 3.3 (3.2–3.6) | 15.9 (13.8–21.9) | 0.1 (0.1–0.2) | 3.4 (3.3–3.6) | 17.8 (14.9–22.2) | 0.2 (0.1–0.2) | **<0.001** | | **0.038** | | 0.745 |
|  | Italian | 3.4 (3.2–3.6) | 17.7 (14.9–21.7) | 0.2 (0.1–0.2) | 3.6 (3.4–3.8) | 18.7 (15.4–22.9) | 0.1 (0.1–0.2) | **<0.001** | | **<0.001** | | **<0.001** |
|  | Chinese | 3.3 (3.2–3.5) | 18.3 (15.4–21.5) | 0.1 (0.1–0.2) | 3.3 (3.2–3.5) | 18.9 (15.7–23.8) | 0.1 (0.1–0.2) | 0.858 | | **0.006** | | **0.003** |
|  | Other | 3.3 (3.2–3.5) | 16.4 (13–20.1) | 0.1 (0.1–0.2) | 3.5 (3.2–3.7) | 18 (14.3–22.9) | 0.1 (0.1–0.2) | **<0.001** | | **<0.001** | | 0.312 |
| **Total** | | 3.4 (3.2–3.6) | 16.8 (13.5–21) | 0.1 (0.1–0.2) | 3.5 (3.3–3.8) | 18.4 (14.7–23.9) | 0.1 (0.1–0.2) | **<0.001** | | **<0.001** | | **0.002** |
| ***Single-Year*** | | | | | | | | | | | | |
| **Variable** | **Outcome** | **Female** | | | **Male** | | | | ***p.*** | | | |
|  |  | **C-Score:**  **Median (IQR)** | **Modified H:**  **Median (IQR)** | **Self-citations:**  **Median (IQR)** | **C-Score:**  **Median (IQR)** | **Modified H:**  **Median (IQR)** | **Self-citations:**  **Median (IQR)** | **C-Score** | | **Modified H-index** | | **Self-citations** |
| **Year** | SEL 2017 | 2.9 (2.8–3.1) | 5.5 (4.7–6.4) | 0.1 (0.1–0.2) | 2.9 (2.8–3.2) | 5.7 (4.8–6.8) | 0.1 (0.1–0.2) | **<0.001** | | **0.019** | | 0.557 |
|  | SEL 2019 | 2.7 (2.5–2.9) | 5.6 (4.8–6.8) | 0.1 (0.1–0.2) | 2.8 (2.6–3) | 5.9 (4.8–7) | 0.1 (0.1–0.2) | **<0.001** | | **<0.001** | | 0.149 |
|  | SEL 2020 | 2.7 (2.5–2.9) | 6 (5–7.5) | 0.1 (0.1–0.2) | 2.8 (2.6–3) | 6.2 (5–7.9) | 0.1 (0.1–0.2) | **<0.001** | | **<0.001** | | 0.218 |
|  | SEL 2021 | 2.6 (2.4–2.8) | 4.9 (4–6) | 0.1 (0.1–0.2) | 2.6 (2.4–2.9) | 5 (4.3–6.7) | 0.1 (0.1–0.2) | **<0.001** | | **<0.001** | | 0.345 |
|  | SEL 2022 | 2.5 (2.4–2.7) | 4.9 (4.1–6) | 0.1 (0–0.1) | 2.6 (2.4–2.9) | 5.1 (4.3–6.7) | 0.1 (0–0.1) | **<0.001** | | **<0.001** | | 0.167 |
|  | SEL 2023 | 2.5 (2.4–2.7) | 4.9 (4–5.9) | 0.1 (0–0.1) | 2.6 (2.4–2.8) | 5 (4.2–6.3) | 0.1 (0–0.1) | **<0.001** | | **<0.001** | | 0.788 |
| **World Bank** | High | 2.6 (2.5–2.9) | 5.1 (4.3–6.4) | 0.1 (0.1–0.2) | 2.7 (2.5–3) | 5.6 (4.6–6.9) | 0.1 (0–0.1) | **<0.001** | | **<0.001** | | **<0.001** |
|  | Upper-middle | 2.5 (2.4–2.7) | 5 (4.3–6.3) | 0.1 (0.1–0.1) | 2.6 (2.4–2.8) | 5.8 (4.8–6.9) | 0.1 (0.1–0.2) | **<0.001** | | **<0.001** | | **<0.001** |
|  | Lower-middle | 2.5 (2.4–2.7) | 5.1 (4.5–6.6) | 0.1 (0.1–0.2) | 2.5 (2.4–2.8) | 5.3 (4.3–6.8) | 0.1 (0.1–0.2) | **0.030** | | 0.377 | | **<0.001** |
|  | Low | 2 (2–2) | 2.3 (2.3–2.3) | 0.3 (0.3–0.3) | 2.6 (2.4–2.7) | 4.4 (3.1–4.9) | 0 (0–0.1) | 0.129 | | 0.386 | | 0.129 |
| **WHO Region** | EURO | 2.6 (2.5–2.8) | 5.1 (4.3–6.2) | 0.1 (0.1–0.2) | 2.7 (2.5–3) | 5.8 (4.8–7) | 0.1 (0.1–0.2) | **<0.001** | | **<0.001** | | **<0.001** |
|  | AMRO | 2.7 (2.5–2.9) | 5 (4.1–6.4) | 0.1 (0–0.1) | 2.7 (2.5–3) | 5.4 (4.4–6.9) | 0.1 (0–0.1) | **<0.001** | | **<0.001** | | **<0.001** |
|  | WPRO | 2.6 (2.4–2.8) | 5.4 (4.5–6.8) | 0.1 (0–0.1) | 2.6 (2.4–2.8) | 5.6 (4.6–6.9) | 0.1 (0.1–0.1) | **<0.001** | | **0.026** | | **<0.001** |
|  | EMRO | 2.5 (2.4–2.7) | 5 (4.3–6) | 0.1 (0.1–0.2) | 2.6 (2.4–2.8) | 5.3 (4.6–6.7) | 0.1 (0.1–0.2) | **<0.001** | | **<0.001** | | **<0.001** |
|  | SEARO | 2.5 (2.4–2.7) | 5.2 (4.5–6.8) | 0.1 (0–0.1) | 2.5 (2.4–2.7) | 5.6 (4.6–6.9) | 0.1 (0–0.2) | 0.198 | | 0.108 | | **<0.001** |
|  | AFRO | 2.6 (2.5–2.8) | 5.6 (5.3–6.3) | 0.1 (0–0.2) | 2.5 (2.4–2.8) | 4.8 (3.9–5.8) | 0.1 (0–0.1) | 0.236 | | **0.018** | | 0.402 |
| **Official Language** | English | 2.7 (2.5–2.9) | 5.1 (4.2–6.7) | 0.1 (0–0.1) | 2.8 (2.5–3) | 5.5 (4.5–6.9) | 0.1 (0–0.1) | **<0.001** | | **<0.001** | | **0.028** |
|  | German | 2.7 (2.5–2.9) | 5.3 (4.7–6.2) | 0.1 (0.1–0.2) | 2.7 (2.5–3) | 5.8 (4.7–7) | 0.1 (0.1–0.2) | **0.005** | | **0.005** | | **0.001** |
|  | Japanese | 2.6 (2.5–2.7) | 5.3 (4.2–6) | 0.1 (0.1–0.2) | 2.6 (2.5–2.8) | 5 (4.3–6) | 0.1 (0.1–0.2) | 0.193 | | 0.983 | | 0.894 |
|  | Italian | 2.6 (2.4–2.8) | 5 (4.3–6) | 0.1 (0.1–0.2) | 2.7 (2.5–3) | 5.8 (4.8–6.9) | 0.1 (0.1–0.2) | **<0.001** | | **<0.001** | | 0.299 |
|  | Chinese | 2.5 (2.4–2.7) | 5 (4.3–6.6) | 0.1 (0–0.1) | 2.5 (2.4–2.7) | 5.5 (4.5–6.9) | 0.1 (0.1–0.1) | **0.001** | | **0.005** | | **<0.001** |
|  | Other | 2.6 (2.4–2.8) | 5.1 (4.3–6.2) | 0.1 (0.1–0.2) | 2.6 (2.5–2.9) | 5.7 (4.7–6.9) | 0.1 (0.1–0.2) | **<0.001** | | **<0.001** | | **<0.001** |
| **Total** | | 2.6 (2.4–2.8) | 5.1 (4.3–6.4) | 0.1 (0.1–0.2) | 2.7 (2.5–2.9) | 5.6 (4.6–6.9) | 0.1 (0.1–0.1) | **<0.001** | | | **<0.001** | 0.638 |

Composite score (C-score) and modified *H*-index were with self-citations excluded. Mann-Whitney (*U*) test was used with a significance level *p.* ≤ 0.05.

**Table S6.** Scholar-level Analysis: Academic Age Stratification of Pharmacologic Scholars in the *Career-Long* Stanford-Elsevier Lists (SEL) of Top 2% Scientists Worldwide (2017–2023)

| **Country** | **SEL 2017** | **SEL 2018** | **SEL 2019** | **SEL 2020** | **SEL 2021** | **SEL 2022** | **SEL 2023** | **Total** |
| --- | --- | --- | --- | --- | --- | --- | --- | --- |
| USA | 37 (30–44) | 39 (31–45) | 36.5 (29–44) | 37 (30–44) | 38 (30–45) | 38 (31–46) | 38 (31–46) | 38 (30–45) |
| GBR | 36 (28–43) | 37 (30–44) | 36 (29–44) | 37 (29–44) | 37 (30–45) | 37 (30–45) | 37 (31–45) | 37 (30–44) |
| JPN | 34 (31–39) | 37 (32–43) | 37 (31.5–43) | 38 (32–44) | 39 (33–44) | 40 (33–46) | 40 (32–45) | 38 (32–44) |
| DEU | 35 (28–42) | 35 (28–44) | 35 (28–42) | 35 (29–43) | 36 (29–42) | 36.5 (29.8–43.2) | 36 (30–44) | 36 (29–43) |
| ITA | 39 (31–43) | 38 (32–43.5) | 38 (32–43) | 38 (32–44) | 38 (32.8–45) | 39 (33–45.8) | 40 (34–46) | 39 (33–45) |
| CAN | 34 (27.2–41) | 37 (31–43) | 37 (29–43) | 35 (27–43) | 36 (29–44) | 37 (29–44) | 37 (30–44.2) | 36 (29–43) |
| FRA | 37 (31–42) | 37 (31–42) | 37 (30–43.5) | 37 (31–42) | 37 (32–43) | 38 (33–44) | 38 (34–44) | 37 (32–44) |
| AUS | 34 (27.8–42.2) | 35 (29.5–43) | 35 (28.5–43) | 35 (27–42) | 36 (28.8–44) | 37 (30–44) | 36 (28–44) | 36 (28–43) |
| NLD | 36 (31–39.5) | 37 (31–40.5) | 35 (30–40) | 35 (30–41) | 36 (30–42) | 37 (31–43) | 36 (31–43) | 36 (30–41) |
| SWE | 40 (34–43) | 41 (35–45.5) | 38 (33.5–45) | 39 (30–45.5) | 40 (32.5–46) | 40 (31–45.2) | 41 (34.5–48.5) | 40 (33–45) |
| CHN | 27.5 (26–34) | 26 (21–29) | 27 (22.2–31) | 27.5 (22.2–32) | 28 (24–34) | 28 (21–32.5) | 28 (21–33) | 28 (22–33) |
| IND | 29.5 (22.8–41.2) | 35.5 (24–44.5) | 30.5 (21–35) | 29.5 (21.8–36.2) | 29 (23–37) | 29 (22–38) | 28 (21–40) | 29 (22–38) |
| KOR | 28.5 (21–32.8) | 27 (23.2–31.8) | 26.5 (22–32) | 26 (22–31.5) | 26 (22–32) | 27 (23–32) | 28 (24–32) | 27 (22–32) |
| CHE | 34 (30–42) | 38 (31–43.8) | 34.5 (27–42.2) | 33.5 (27–40) | 34 (25.8–40.2) | 36 (27.8–42) | 36 (28–42) | 35 (27–42) |
| BEL | 36 (28–42) | 37 (26–43) | 33.5 (28–41.2) | 34 (28–42) | 33.5 (28–41.8) | 35 (29–42) | 35 (28–42) | 35 (28–42) |
| ESP | 32 (25–38) | 33 (28.8–40.2) | 33 (29–38) | 34 (29–39) | 34.5 (30–39) | 36 (31–40.8) | 36.5 (32.2–41) | 35 (29–40) |
| DNK | 35.5 (31–40.5) | 35 (31–42) | 30 (26–41) | 32 (26–40) | 32 (26–39.5) | 32.5 (26–40) | 32 (26–39) | 32 (26–40) |
| NZL | 27 (22–33) | 19 (15–31) | 19 (14–27.8) | 19 (12–31) | 19 (12–32) | 18.5 (10–32.5) | 18 (10–30) | 19 (12–31) |
| POL | 40 (35–46.2) | 39 (33.5–45.2) | 40 (30.2–43.5) | 39 (29–46) | 40 (30–47) | 39 (29.5–46) | 40 (31–46.8) | 40 (30–46) |
| FIN | 38 (27–41) | 39 (31.5–43.5) | 37.5 (29–41.8) | 38 (28–43) | 39 (30.2–43) | 40 (31–44) | 39 (30.2–44) | 38.5 (29–43) |
| TWN | 30.5 (27–37.5) | 31 (28–36) | 33.5 (29.2–36) | 36 (30–39) | 35 (29.5–40.5) | 36 (31.8–42.2) | 36 (30–43) | 35 (29–39) |
| AUT | 38 (28.8–41.8) | 40 (29.5–44) | 31 (28–40) | 33 (28–43) | 34 (29–41) | 34 (29–41) | 34 (29–39) | 34 (29–41) |
| IRN | 23 (21.2–39.8) | 46 (34–46) | 26 (21.5–28) | 21 (14.2–25.8) | 22 (16.5–27.5) | 23.5 (17–27.8) | 22 (16.2–28) | 23 (17–28) |
| BRA | 41 (26–46.5) | 41 (27–51) | 32 (27–43) | 30 (24–39) | 30.5 (26.5–42.2) | 33 (27.5–42.5) | 31 (27–39) | 31 (26–43) |
| ISR | 38.5 (34–44) | 41 (35–43) | 40.5 (33.5–43) | 40.5 (35.5–44) | 42 (36.5–47.2) | 43 (37–49) | 42.5 (28.8–48) | 41 (35–46) |
| HUN | 41 (35–51.5) | 45 (40.8–50.8) | 42.5 (33.2–52) | 44 (38–54) | 45 (38–53.5) | 46 (34.8–54) | 46.5 (35.5–54.8) | 45 (37–54) |
| IRL | 34 (27–41) | 33 (25–42) | 30 (25.2–38) | 32 (25–39) | 33 (27.8–42.2) | 34 (28.5–40.5) | 33 (29.5–37.5) | 33 (26–40) |
| EGY | 46 (46–46) | 47 (47–47) | 24.5 (18.2–29) | 28 (22.2–31.8) | 27 (20.5–30.5) | 28 (22–33) | 25 (19–31) | 28 (19.5–32.5) |
| SAU | *NA* | *NA* | 28 (20.2–31.5) | 24.5 (18.2–31.8) | 22 (18.5–32.2) | 22 (17–32) | 21 (16–29) | 22 (17–32) |
| PRT | 31.5 (25.2–35.5) | 26 (21–32) | 27.5 (16.8–31) | 28.5 (17.8–32.8) | 29 (21–33) | 29 (25.5–33) | 31 (25–35) | 29 (20.5–34) |
| SGP | 34 (24–37.5) | 39 (16–42) | 34.5 (32–38.8) | 33 (26–38) | 36 (32–41) | 36 (25–42) | 35 (25.8–42) | 36 (26–41) |
| NOR | 44 (37–51) | 45 (33–47) | 38 (25.8–46) | 38.5 (32.8–46) | 39 (30–43) | 38 (27.8–45.8) | 40 (30–48.2) | 39 (30.2–47) |
| HKG | 25 (20.8–30.8) | 28 (25–35.5) | 29 (24–33) | 32 (27–41) | 31.5 (27.5–34.8) | 32 (26.5–38.5) | 31.5 (27.5–36.8) | 30 (25–36) |
| TUR | 28.5 (22–38) | 23 (22.5–23.5) | 31 (25.8–36.2) | 31 (26.2–32.2) | 31 (27–36) | 30 (26–36) | 31 (27–38) | 31 (25–36) |
| GRC | 25 (24–36) | 31.5 (29–36.8) | 31 (28.5–32.2) | 32.5 (27.2–35.8) | 32 (27.8–35) | 34.5 (29.8–38.2) | 35 (29.5–37.5) | 33 (28–36) |
| CZE | 41.5 (41.2–41.8) | 41 (34–44) | 29.5 (23–39.2) | 35.5 (23–43.2) | 35 (24–44) | 36.5 (25–43.5) | 37 (26–45) | 37 (26–44) |
| THA | 32 (26.5–37.5) | 22 (22–22) | 32.5 (25–35.5) | 29 (23.2–38.5) | 33.5 (26.5–38) | 34 (26–38) | 29 (24.8–37.2) | 31.5 (24.2–38) |
| MYS | *NA* | *NA* | 26 (23–28) | 19 (12.2–27) | 21 (16–28) | 22 (13–30) | 19 (15–25) | 21.5 (14.5–28.5) |
| ZAF | 18 (17–24) | 19 (19–25) | 20 (19–32) | 23 (20–28.5) | 22 (19.8–24.2) | 25 (22–29) | 25 (20–29) | 23 (19–30) |
| PAK | 30 (30–30) | 31 (30.5–36.5) | 26.5 (20.8–30.5) | 15 (12–33) | 16 (15–34) | 22 (14.8–32) | 20 (16–35) | 25 (14.8–34) |
| MEX | 29 (25.5–32.5) | 34 (32–36) | 34 (31–38) | 33.5 (31.2–40.8) | 33 (28–35.8) | 34 (27–38.5) | 35 (28–39.5) | 34 (29.5–39) |
| NGA | 29 (29–29) | 30 (30–30) | 33 (32–34) | 34 (26.2–37.2) | 37.5 (34–41.8) | 38 (30–44.5) | 36 (28.5–42) | 35 (29–43) |
| ISL | 37 (37–37) | 38 (38–38) | 39 (39–39) | 40 (40–40) | 33 (32–41) | 37.5 (31.5–42) | 36 (32.5–43) | 38 (33–41) |
| ARE | 46 (42.5–49.5) | 55 (55–55) | 29 (23–42) | 28.5 (27.8–29.2) | 27 (25–29) | 27 (20–28) | 23 (18–31.5) | 27.5 (21.5–32.2) |
| KWT | 29 (29–29) | *NA* | 35.5 (32.2–38.8) | 40 (37.8–40.2) | 40 (34–40.8) | 41 (40–41) | 41 (40–42) | 40 (33.5–41) |
| RUS | *NA* | 44 (36–45.5) | 41 (29–44) | 45 (37.5–46) | 28.5 (22.8–34.5) | 32 (29.5–38.5) | 33 (30.5–40.5) | 33 (28–45) |
| MAC | *NA* | *NA* | 26.5 (23–33) | 27 (26–28) | 18.5 (17.2–20.5) | 18 (17–19) | 21 (19–25) | 21 (17.5–26.5) |
| PSE | *NA* | *NA* | 16.5 (15.2–17.8) | 18 (16.5–19.5) | 16 (16–19) | 17 (15.5–20) | 18 (18–24) | 18 (15.5–21.5) |
| QAT | *NA* | 40 (35–45) | 40.5 (35.8–45.2) | 42 (37–47) | 42.5 (37.2–47.8) | 44 (39–49) | 30 (23.2–40) | 34.5 (31.2–51.5) |
| JOR | *NA* | *NA* | 25 (25–25) | 29.5 (27.8–31.2) | 30.5 (28.8–32.2) | 27 (23.5–31.2) | 28.5 (24.5–33) | 27 (26–33) |
| BGD | *NA* | *NA* | *NA* | 12 (12–12) | 14 (12.5–24) | 35 (23.5–35) | 27 (16.8–36) | 18 (12.5–35) |
| HRV | *NA* | *NA* | 44 (44–44) | 41.5 (38.8–44.2) | 42.5 (39.8–45.2) | 38 (31–43) | 39 (32–44.5) | 39 (36.5–47.5) |
| ROU | *NA* | *NA* | 37.5 (30.2–44.8) | 24 (24–24) | 26.5 (25.8–27.2) | 26 (19.5–36.5) | 27 (20.5–37) | 26 (23.5–37.5) |
| SRB | *NA* | *NA* | *NA* | 35 (32.5–35) | 35 (33–35.5) | 33.5 (32.8–34.2) | 34.5 (33.8–35.2) | 35 (32.2–35) |
| SVK | 41 (41–41) | 46 (46–46) | *NA* | 43.5 (40.8–46.2) | 47 (45.5–48.5) | 48 (46.5–49.5) | 48.5 (47.2–49.8) | 46 (44.2–49.8) |
| BHR | 15 (15–15) | 15 (15–15) | 17 (17–17) | 18 (18–18) | 19 (19–19) | 23.5 (21.8–25.2) | 27.5 (24.2–30.8) | 19 (17–21) |
| IRQ | *NA* | *NA* | *NA* | *NA* | 12.5 (11.2–13.8) | 11 (10.5–13.5) | 12.5 (11.5–13) | 12 (10–13) |
| TTO | *NA* | *NA* | 14 (14–14) | 20.5 (17.8–23.2) | 21 (18.5–23.5) | 21.5 (19.2–23.8) | 23.5 (20.8–26.2) | 18 (16–26) |
| URY | 51 (51–51) | *NA* | *NA* | 45 (40–50) | 45.5 (40.2–50.8) | 46 (40.5–51.5) | 49.5 (45.8–53.2) | 51 (35–56) |
| OMN | 39 (39–39) | 39 (39–39) | 41 (41–41) | 39 (39–39) | 43 (43–43) | 39 (39–39) | 32 (25.5–38.5) | 39 (39–41.5) |
| SVN | *NA* | *NA* | *NA* | 41 (41–41) | 32 (27–37) | 33 (28–38) | 28 (26–36) | 34.5 (23.8–42.2) |
| CYP | *NA* | 36 (36–36) | 34 (34–34) | 38 (38–38) | 39 (39–39) | 34.5 (34.2–34.8) | 41 (41–41) | 36 (34.5–38.5) |
| *NA*M | *NA* | 43 (43–43) | 44 (44–44) | 46 (46–46) | 46 (46–46) | 45 (43.5–46.5) | 48 (48–48) | 46 (43.5–47) |
| ARG | *NA* | 42 (38.5–45.5) | *NA* | 15 (15–15) | 16 (16–16) | 18 (18–18) | 18 (18–18) | 18 (16.5–30.8) |
| BGR | *NA* | 36 (36–36) | 31 (31–31) | 32 (32–32) | 33 (33–33) | 34 (34–34) | 35 (35–35) | 33.5 (32.2–34.8) |
| VEN | *NA* | *NA* | 49 (49–49) | 43 (40–46) | 39 (39–39) | 30 (30–30) | 30 (30–30) | 38 (31.8–46.5) |
| CUB | *NA* | *NA* | 20 (20–20) | 20 (20–20) | 20 (20–20) | 20 (20–20) | 20 (20–20) | 20 (20–20) |
| ECU | *NA* | *NA* | 16 (16–16) | 17 (17–17) | 18 (18–18) | 19 (19–19) | 20 (20–20) | 18 (17–19) |
| IDN | *NA* | *NA* | 11 (11–11) | 12 (12–12) | 13 (13–13) | 14 (14–14) | 15 (15–15) | 13 (12–14) |
| PRI | *NA* | *NA* | *NA* | 26 (26–26) | 27 (27–27) | 28 (28–28) | 29 (29–29) | 28 (27–28) |
| MLT | *NA* | *NA* | 46 (46–46) | *NA* | 48 (48–48) | 49 (49–49) | 50 (50–50) | 48.5 (47.5–49.2) |
| MWI | *NA* | 17 (17–17) | 17 (17–17) | 33 (33–33) | *NA* | 35 (35–35) | *NA* | 25 (17–33.5) |
| NPL | *NA* | *NA* | *NA* | 34 (34–34) | 38 (38–38) | 38 (38–38) | 38 (38–38) | 38 (37–38) |
| VUT | *NA* | *NA* | *NA* | 40 (40–40) | 40 (40–40) | 40 (40–40) | 40 (40–40) | 40 (40–40) |
| GEO | *NA* | *NA* | 41 (41–41) | 41 (41–41) | *NA* | *NA* | 25 (25–25) | 41 (33–41) |
| LUX | *NA* | 43 (43–43) | 43 (43–43) | 44 (44–44) | *NA* | *NA* | *NA* | 43 (43–43.5) |
| VNM | *NA* | *NA* | *NA* | 22 (22–22) | 23 (23–23) | 24 (24–24) | *NA* | 23 (22.5–23.5) |
| EST | *NA* | *NA* | *NA* | *NA* | 26 (26–26) | *NA* | 28 (28–28) | 27 (26.5–27.5) |
| ETH | *NA* | *NA* | 25 (25–25) | *NA* | 40 (40–40) | *NA* | *NA* | 32.5 (28.8–36.2) |
| CHL | *NA* | 49 (49–49) | *NA* | *NA* | *NA* | *NA* | *NA* | 49 (49–49) |
| COG | *NA* | *NA* | 12 (12–12) | *NA* | *NA* | *NA* | *NA* | 12 (12–12) |
| LBN | 33 (33–33) | *NA* | *NA* | *NA* | *NA* | *NA* | *NA* | 33 (33–33) |
| LBR | *NA* | *NA* | *NA* | *NA* | *NA* | 30 (30–30) | *NA* | 30 (30–30) |
| MAR | *NA* | *NA* | *NA* | *NA* | *NA* | *NA* | 31 (31–31) | 31 (31–31) |
| UGA | *NA* | *NA* | *NA* | *NA* | 47 (47–47) | *NA* | *NA* | 47 (47–47) |
| UKR | *NA* | *NA* | *NA* | *NA* | 24 (24–24) | *NA* | *NA* | 24 (24–24) |
| YEM | *NA* | *NA* | 29 (29–29) | *NA* | *NA* | *NA* | *NA* | 29 (29–29) |
| *NA* | 34 (27–41) | 34 (34–37) | 32.5 (28–37.8) | 39 (30–42) | 37.5 (30.8–39.8) | 39 (31.5–41.5) | 37 (23–47) | 34.5 (27–41) |
| **Total** | 36 (29–43) | 37 (30–44) | 35 (28–43) | 36 (28–43) | 36 (29–43) | 36 (29–44) | 36 (29–44) | 36 (29–43) |

Median (Interquartile Range)

**Table S7.** Scholar-level Analysis: Academic Age Stratification of Pharmacologic Scholars in the *Single-Year* Stanford-Elsevier Lists (SEL) of Top 2% Scientists Worldwide (2017–2023)

| **Country** | **SEL 2019** | **SEL 2020** | **SEL 2021** | **SEL 2022** | **SEL 2023** | **Total** |
| --- | --- | --- | --- | --- | --- | --- |
| USA | 31 (22–40) | 32 (22–41.8) | 32 (22–42) | 33 (23–43) | 33 (23–43) | 32 (22–42) |
| GBR | 33 (25–42) | 33 (24–43) | 33 (24–43) | 33 (24–43) | 33 (24–43) | 33 (24–43) |
| CHN | 19 (14–25) | 18 (14–25) | 19 (15–26) | 19 (15–25) | 19 (15–24) | 19 (15–25) |
| IND | 20 (13–28.5) | 17 (12–26) | 17 (12–25) | 17 (13–25) | 18 (14–26) | 18 (13–26) |
| ITA | 30 (22–38) | 31 (21.8–38.2) | 30 (21–38) | 30 (20–39) | 30 (20–39) | 30 (21–39) |
| DEU | 29 (23–36) | 30 (24–37) | 30.5 (23.8–38) | 31 (25–39) | 32 (25–40) | 31 (24–38) |
| JPN | 34 (24–39) | 33 (25–40) | 34 (25–41) | 35 (26–43) | 35 (26–42) | 34 (25–41) |
| IRN | 15.5 (11–22) | 14.5 (9–21) | 16 (10–21.8) | 15 (11–21) | 16 (11–20) | 15 (11–21) |
| KOR | 22 (17–27) | 23 (18–27) | 23 (19–28) | 24 (19–29) | 24 (18.2–29) | 23 (18–28) |
| AUS | 25 (18–35) | 25 (18.8–36) | 25 (19–36) | 27 (18–36.5) | 25 (18–37) | 25 (18–36) |
| CAN | 30 (24–40) | 31 (24–41) | 30 (23–39) | 30.5 (23.2–40) | 32 (22.2–41) | 31 (23–40) |
| FRA | 32 (24–40) | 32 (26–41) | 33 (25.5–41) | 34 (27–42.2) | 34 (26–41) | 33 (26–41) |
| NLD | 30 (22–34) | 31 (23–38) | 31 (23–38.2) | 32 (24–39) | 30 (24–37) | 30 (23–37) |
| SAU | 14 (11–18.2) | 14 (11–19.2) | 13.5 (11–19) | 14 (12–18.5) | 14 (11–18) | 14 (11–19) |
| ESP | 32 (28–35) | 33 (27–35.5) | 33 (28–37) | 33 (28–38) | 34 (28.8–39) | 33 (28–37) |
| EGY | 17 (12–23) | 16 (12–22) | 16 (13–23) | 16 (13–22.8) | 15 (12–20) | 16 (12–22) |
| CHE | 30 (22–38) | 29 (23–35) | 30 (24–39.5) | 31 (24–41) | 31.5 (25–38.8) | 30 (23–38) |
| BRA | 23.5 (18–31.5) | 22 (17–31) | 23 (19–32) | 24 (20–32.5) | 24 (19–31) | 23 (19–32) |
| BEL | 27 (19.2–33.5) | 27 (19–36.5) | 26.5 (20–33) | 28 (21–36.2) | 28.5 (20.2–35) | 27 (20–35) |
| NZL | 17 (8–25) | 16.5 (8–25.2) | 16 (8.5–26.5) | 16 (9–22.8) | 16 (10–23) | 16 (8–25) |
| POL | 28 (21–40) | 27 (22–39.5) | 26 (22–39) | 29 (24–40.5) | 28 (23–39) | 28 (22–40) |
| DNK | 26 (18.5–36) | 27 (18–36.5) | 26.5 (17.8–35.2) | 25 (16–33.5) | 27 (20.2–35.8) | 26 (18–36) |
| SWE | 32.5 (24–40.2) | 34 (24.5–44) | 39 (26–47) | 37 (26.5–49) | 37 (25–47.5) | 35 (25–45) |
| PRT | 22.5 (15.2–27.8) | 18 (13–28.5) | 18.5 (14.2–29) | 19.5 (14.2–29) | 21 (16–30) | 20 (14–29) |
| PAK | 14 (10.2–21.8) | 12 (10–15) | 14 (11.2–20) | 13 (12–21) | 14 (13–21) | 14 (12–20) |
| AUT | 27 (25–31.8) | 28 (23.2–33.8) | 29 (21.5–34) | 28.5 (19.5–34.8) | 29 (20–35.5) | 28.5 (21–34) |
| FIN | 28 (19–38) | 29 (20–41) | 28.5 (18.8–42) | 30 (20.5–42.5) | 30 (20–40) | 29 (19–41) |
| TWN | 29 (21.2–35) | 32.5 (26.8–36.2) | 32 (23.5–37) | 32 (20–38) | 31 (19.2–38.5) | 31 (21–37) |
| MYS | 14 (10.5–19.5) | 15 (11–23.5) | 16 (13.5–22.5) | 19 (15–24) | 16 (14–20.2) | 17 (13–22) |
| ISR | 38.5 (24.8–43.8) | 37 (25–43.5) | 38.5 (26–45.8) | 41.5 (29–47) | 40 (27–48) | 39 (26–46) |
| THA | 28 (21–33) | 23.5 (19–32) | 23 (18–33) | 24 (16.5–30) | 24 (16.2–30.2) | 24 (17–32.5) |
| IRL | 25 (17.5–31) | 24 (18.8–35.5) | 22.5 (15–34.8) | 23.5 (16.2–31.8) | 22.5 (15.8–30.5) | 23 (16–32) |
| GRC | 29 (20–32) | 29 (22–33) | 29 (23–34) | 30 (21.5–34.5) | 29 (21–34) | 29 (21–34) |
| SGP | 23 (16.2–36.2) | 23 (17.5–36.5) | 29.5 (19.5–39.2) | 27 (21–40) | 28 (21–41) | 26 (18.5–38.5) |
| TUR | 25.5 (20.8–30) | 25.5 (17.5–30.8) | 23 (18–31) | 26 (17–30) | 27 (16.5–32) | 25 (17–31) |
| ZAF | 20 (15–22) | 20 (15–23.8) | 20 (13.8–23.5) | 19.5 (14.2–24) | 18.5 (14–24.2) | 19 (14–24) |
| HKG | 29 (21–36) | 25 (17–38) | 23 (15–35) | 29 (18.8–38) | 29 (24–37) | 28 (18–37) |
| HUN | 40 (27.5–42.5) | 33 (25.5–43.2) | 32.5 (29.2–44.8) | 32.5 (27.5–45) | 32.5 (26.2–45.5) | 33 (28–44) |
| ARE | 17 (12.5–23) | 17 (15–24) | 18 (16–23) | 19 (16.5–23.2) | 18.5 (12–21.2) | 18 (13.5–23) |
| NOR | 26 (22–31.8) | 27.5 (23–35.2) | 29 (24.8–34.5) | 25 (17.2–33.2) | 26 (17–32) | 26 (20–34) |
| JOR | 16 (15–25) | 16.5 (11.8–19.2) | 17.5 (13–25.2) | 14 (11.8–18.2) | 15 (12.5–18.5) | 15 (12–22.2) |
| IRQ | 8 (6–9) | 9 (7–10.8) | 11 (10.8–12) | 12 (9.5–12.5) | 12 (10–13) | 10 (8–13) |
| CZE | 22 (18.8–35.5) | 25.5 (21.2–39.5) | 23.5 (11.8–35.2) | 25 (24–39) | 26 (25.2–34.2) | 25 (20.5–39) |
| MEX | 24 (20–32) | 29 (20.5–33.5) | 33 (20–33.5) | 25 (15–34.2) | 24 (16–31.5) | 26.5 (16–34) |
| PSE | 14 (14–14) | 15 (13–18.5) | 17 (16–22.2) | 17 (14.8–22) | 18 (14–24) | 16 (14–21) |
| MAC | 25.5 (17.5–27.5) | 16 (14–25) | 17 (15.5–18.8) | 16 (14.8–18.5) | 19 (16.5–22) | 17 (15–23.2) |
| ROU | 16 (16–16) | 15 (11.5–17) | 18 (18–20) | 17 (15.2–19.2) | 18 (13–20) | 17 (13.8–20) |
| NGA | *NA* | 21 (16.5–22.5) | 13.5 (12.2–23) | 15 (10.8–18.5) | 15 (13–21) | 15 (12.2–22.5) |
| QAT | 30.5 (26.2–35.8) | 31.5 (27.2–37) | 32 (17–32) | 26 (16.5–31) | 27 (18–29) | 28 (17–32) |
| IDN | 11 (11–11) | 14.5 (10.2–21.2) | 15 (10.5–21.5) | 12 (5.8–15.2) | 12 (9.5–17) | 12.5 (6.8–17.5) |
| RUS | 23 (21–24) | 22 (19.5–26) | 11 (3.5–24.5) | 24 (20–27) | 20 (6.5–26.5) | 22 (9.5–26.5) |
| ISL | 35.5 (31.2–39) | 40 (35.5–40) | 33 (32–41) | 42 (32–45.5) | 39.5 (34.5–43) | 39 (32–42) |
| SVN | 14.5 (12–21.2) | 15 (13–21.5) | 22 (15–26) | 19 (17–27) | 24 (18–28) | 18 (14.5–27.5) |
| BGD | 14.5 (9.8–19.2) | 12 (8.5–14) | 14 (12.5–15.5) | 18 (14.5–28) | 18 (11.5–20.5) | 16.5 (12–20.5) |
| SRB | 31.5 (30.2–32.8) | 30 (24–35) | 31 (25–35) | 17 (16–32) | 27 (17–33) | 29.5 (18.8–34.8) |
| HRV | 33 (27.5–38.5) | 41.5 (38.8–44.2) | 34 (24–37) | 24 (24–38) | 26 (22–39) | 34 (23.5–41.5) |
| KWT | 29 (29–29) | 35.5 (28–40.2) | 27 (21.5–34.2) | 33 (23–41) | 37.5 (31.5–41.8) | 32.5 (23.2–41) |
| ARG | 15.5 (14.8–16.2) | 16.5 (15.8–17.2) | 16 (13.5–17.5) | 17 (14.8–18.5) | 17.5 (12.5–20.2) | 17 (14–18) |
| VNM | 9.5 (8.8–10.2) | 12 (10.5–17) | 13 (11.5–16.2) | 13 (12–18.5) | 10 (8–12.2) | 12 (9.8–13.2) |
| OMN | 41 (41–41) | 16 (14–27.5) | 23 (18.5–33) | 22.5 (20.2–27.8) | 23.5 (21.2–30) | 23 (18.5–39) |
| LBN | 11 (10–12) | 12 (11–13) | 11 (11–13) | 12 (12–14) | 15 (14–16) | 13 (11–15) |
| BHR | 17 (17–17) | 13.5 (11.2–15.8) | 15 (13–17) | 20 (16–23.5) | 21 (17–27.5) | 18 (12.5–20.5) |
| ETH | *NA* | 4 (4–4) | 5 (4–22.5) | 3.5 (2.8–4.2) | 7 (3–14) | 5 (3–10.5) |
| BGR | 31 (31–31) | 26 (23–29) | 27 (24–30) | 28 (25–31) | 29 (26–32) | 31 (22–33) |
| LKA | 9 (9–9) | 9 (9–9) | 17 (17–17) | 17 (17–17) | 17 (17–17) | 17 (9–17) |
| MAR | *NA* | 17.5 (12.2–22.8) | 18 (12.5–23.5) | 19.5 (14.2–24.8) | 9 (9–20) | 9 (9–29) |
| UKR | *NA* | 7 (6–8) | 4.5 (4–19.2) | 2 (2–2) | *NA* | 5 (4–9) |
| MLT | 46 (46–46) | *NA* | 28.5 (18.8–38.2) | 35.5 (28.8–42.2) | 23 (17–36.5) | 34.5 (19.2–48.2) |
| CYP | *NA* | *NA* | 10 (10–10) | 34 (22.5–34.5) | 26.5 (19.2–33.8) | 23 (11.2–34.8) |
| LTU | *NA* | 19 (19–19) | 20 (20–20) | 21.5 (21.2–21.8) | 22.5 (22.2–22.8) | 21.5 (20.2–22) |
| NPL | *NA* | 34 (34–34) | 38 (38–38) | 24.5 (17.8–31.2) | 25 (18.5–31.5) | 36 (17.5–38) |
| PER | *NA* | *NA* | 17 (16–18) | 18 (17–19) | 19 (18–20) | 18 (16.2–19.8) |
| ECU | 16 (16–16) | 17 (17–17) | 18 (18–18) | 19 (19–19) | 20 (20–20) | 18 (17–19) |
| EST | *NA* | 33 (33–33) | 27.5 (26.8–28.2) | *NA* | 29.5 (28.8–30.2) | 29 (28–31) |
| MKD | 16 (16–16) | 16 (16–16) | 16 (16–16) | 16 (16–16) | 16 (16–16) | 16 (16–16) |
| *NA*M | 44 (44–44) | 46 (46–46) | 46 (46–46) | 48 (48–48) | 48 (48–48) | 46 (46–48) |
| SVK | *NA* | 38 (38–38) | *NA* | 34 (27.5–40.5) | 35 (28.5–41.5) | 38 (22–47) |
| CHL | *NA* | 15 (15–15) | 16 (16–16) | 13 (13–13) | 14 (14–14) | 14.5 (13.8–15.2) |
| LVA | *NA* | 23 (23–23) | 24 (24–24) | 25 (25–25) | 26 (26–26) | 24.5 (23.8–25.2) |
| URY | *NA* | 55 (55–55) | 56 (56–56) | 57 (57–57) | 57 (57–57) | 56.5 (55.8–57) |
| COL | *NA* | *NA* | 16 (16–16) | 17 (17–17) | 17 (17–17) | 17 (16.5–17) |
| MWI | 17 (17–17) | 33 (33–33) | *NA* | 35 (35–35) | *NA* | 33 (25–34) |
| PRI | *NA* | 26 (26–26) | 27 (27–27) | 28 (28–28) | *NA* | 27 (26.5–27.5) |
| SYR | *NA* | *NA* | 10 (10–10) | 11 (11–11) | 11 (11–11) | 11 (10.5–11) |
| TTO | *NA* | 15 (15–15) | 16 (16–16) | *NA* | 18 (18–18) | 16 (15.5–17) |
| BRN | *NA* | 11 (11–11) | 12 (12–12) | *NA* | *NA* | 11.5 (11.2–11.8) |
| GEO | 41 (41–41) | 41 (41–41) | *NA* | *NA* | *NA* | 41 (41–41) |
| KAZ | *NA* | *NA* | *NA* | 17 (17–17) | 18 (18–18) | 17.5 (17.2–17.8) |
| LUX | 43 (43–43) | 44 (44–44) | *NA* | *NA* | *NA* | 43.5 (43.2–43.8) |
| ALB | *NA* | *NA* | *NA* | *NA* | 12 (12–12) | 12 (12–12) |
| AZE | *NA* | *NA* | *NA* | *NA* | 49 (49–49) | 49 (49–49) |
| GHA | *NA* | *NA* | *NA* | 11 (11–11) | *NA* | 11 (11–11) |
| LBR | *NA* | *NA* | *NA* | 30 (30–30) | *NA* | 30 (30–30) |
| SDN | *NA* | *NA* | *NA* | *NA* | 13 (13–13) | 13 (13–13) |
| TUN | *NA* | *NA* | 16 (16–16) | *NA* | *NA* | 16 (16–16) |
| ZMB | *NA* | *NA* | *NA* | *NA* | 13 (13–13) | 13 (13–13) |
| *NA* | 34.5 (20.5–40.8) | 23 (15.5–39) | 11 (8.5–32.5) | 11 (8–35) | 22 (8.5–38.5) | 23.5 (13.2–40) |
| **Total** | 28 (19–37) | 27 (18–37) | 27 (18–37) | 27 (17–37) | 26 (17–37) | 27 (18–37) |

Median (Interquartile Range)

**Table S8.** Individual-level Analyses: Correlations Between Academic Age and Scholarly Output Metrics of Pharmacologic Scholars in the Stanford–Elsevier Lists (2017–2023)

| **Scholarly Output Metric** | ***Career-Long* SEL** | | | | | |
| --- | --- | --- | --- | --- | --- | --- |
|  | **Overall** | **Gender** | | **Language** | | |
|  |  | **Female** | **Male** | **Non-English Speaking** | **English Speaking** |  |
| Total Citations ^╪^ | 0.186** | 0.147** | 0.185** | 0.173** | 0.200** |  |
| Modified *H*-index ^╪^ | 0.330** | 0.290** | 0.335** | 0.296** | 0.356** |  |
| Composite Score ^╪^ | 0.277** | 0.233** | 0.273** | 0.257** | 0.279** |  |
| Self-citations (%) | -0.072** | 0.035* | -0.103** | -0.078** | -0.033** |  |
| Total Papers | 0.377** | 0.283** | 0.375** | 0.357** | 0.434** |  |
| Single-authored Papers (Number) | 0.395** | 0.233** | 0.405** | 0.399** | 0.375** |  |
| Single-authored Papers (Citations) ^╪^ | 0.213** | 0.167** | 0.209** | 0.181** | 0.215** |  |
| Single- and First-authored Papers (Number) | 0.410** | 0.285** | 0.412** | 0.410** | 0.409** |  |
| Single- and First-authored Papers (Citations) ^╪^ | -0.01 | -0.070** | 0.002 | -0.046** | 0.008 |  |
| Single-, First-, and Last-authored Papers (Number) | 0.467** | 0.345** | 0.471** | 0.455** | 0.495** |  |
| Single-, First-, and Last-authored Papers (Citations) ^╪^ | 0.249** | 0.214** | 0.252** | 0.237** | 0.256** |  |
| **Scholarly Output Metric** | ***Single-Year* SEL** | | | | | |
|  | **Overall** | **Gender** | | **Language** | | |
|  |  | **Female** | **Male** | **Non-English Speaking** | **English Speaking** |  |
| Total Citations ^╪^ | 0.117** | 0.178** | 0.092** | 0.168** | 0.067** |  |
| Modified *H*-index ^╪^ | 0.177** | 0.242** | 0.147** | 0.190** | 0.172** |  |
| Composite Score ^╪^ | 0.243** | 0.233** | 0.234** | 0.202** | 0.223** |  |
| Self-citations (%) | -0.223** | -0.080** | -0.269** | -0.184** | -0.194** |  |
| Total Papers | 0.621** | 0.572** | 0.616** | 0.637** | 0.636** |  |
| Single-authored Papers (Number) | 0.586** | 0.405** | 0.622** | 0.515** | 0.586** |  |
| Single-authored Papers (Citations) ^╪^ | 0.369** | 0.250** | 0.388** | 0.285** | 0.372** |  |
| Single- and First-authored Papers (Number) | 0.547** | 0.404** | 0.568** | 0.488** | 0.568** |  |
| Single- and First-authored Papers (Citations) ^╪^ | -0.146** | -0.208** | -0.123** | -0.204** | -0.120** |  |
| Single-, First-, and Last-authored Papers (Number) | 0.682** | 0.610** | 0.686** | 0.682** | 0.673** |  |
| Single-, First-, and Last-authored Papers (Citations) ^╪^ | 0.199** | 0.240** | 0.183** | 0.230** | 0.156** |  |

^╪^ Self-citations were excluded. ** Correlation is significant at the 0.01 level (2-tailed).
